# Analysis of the unexplored features of *rrs *(16S rDNA) of the Genus *Clostridium*

**DOI:** 10.1186/1471-2164-12-18

**Published:** 2011-01-11

**Authors:** Vipin Chandra Kalia, Tanmoy Mukherjee, Ashish Bhushan, Jayadev Joshi, Pratap Shankar, Nusrat Huma

**Affiliations:** 1 Microbial Biotechnology and Genomics, Institute of Genomics and Integrative Biology (IGIB), CSIR, Delhi University Campus, Mall Road, Delhi-110007, India

## Abstract

**Background:**

Bacterial taxonomy and phylogeny based on *rrs *(16S rDNA) sequencing is being vigorously pursued. In fact, it has been stated that novel biological findings are driven by comparison and integration of massive data sets. In spite of a large reservoir of *rrs *sequencing data of 1,237,963 entries, this analysis invariably needs supplementation with other genes. The need is to divide the genetic variability within a taxa or genus at their *rrs *phylogenetic boundaries and to discover those fundamental features, which will enable the bacteria to naturally fall within them. Within the large bacterial community, *Clostridium *represents a large genus of around 110 species of significant biotechnological and medical importance. Certain *Clostridium *strains produce some of the deadliest toxins, which cause heavy economic losses. We have targeted this genus because of its high genetic diversity, which does not allow accurate typing with the available molecular methods.

**Results:**

Seven hundred sixty five *rrs *sequences (> 1200 nucleotides, nts) belonging to 110 *Clostridium *species were analyzed. On the basis of 404 *rrs *sequences belonging to 15 *Clostridium *species, we have developed species specific: (i) phylogenetic framework, (ii) signatures (30 nts) and (iii) *in silico *restriction enzyme (14 Type II REs) digestion patterns. These tools allowed: (i) species level identification of 95 *Clostridium *sp. which are presently classified up to genus level, (ii) identification of 84 novel *Clostridium *spp. and (iii) potential reduction in the number of *Clostridium *species represented by small populations.

**Conclusions:**

This integrated approach is quite sensitive and can be easily extended as a molecular tool for diagnostic and taxonomic identification of any microbe of importance to food industries and health services. Since rapid and correct identification allows quicker diagnosis and consequently treatment as well, it is likely to lead to reduction in economic losses and mortality rates.

## Background

Bacterial identification becomes a challenge particularly in case they are either involved in an industrial process with heavy investments at risk or are a serious threat to human beings. Sequencing of the *rrs *(16S rDNA) of bacteria is vigorously pursued for correct identification and classification [1-3]. It has led to a large database of 1,237,963 entries http://rdp.cme.msu.edu/. The key questions which we are addressing: Whether there are certain latent and as yet un-explored features in the nucleotide sequences of the *rrs*, which can be used to define the phylogenetic limits of a particular genus or species? Are there certain unique patterns of nucleotide strings (length and order) and signatures within them, which can enable tracking the identity of an organism within the phylogenetic framework? In fact, it has been stated that novel biological findings are being driven by comparison and integration of massive data sets [4]. They also predict that numerous tools will be designed to use such large and well organized data. Comparative analyses of *rrs *sequences of *Clostridium *spp. can be exploited to develop molecular tools for defining the genetic variability and tracking the evolutionary events.

*Clostridium *represents a large bacterial genus of significant biotechnological as well as medical importance. The diversity of its economic importance varies from production of solvents - *Clostridium beijerinckii, C. acetobutylicum, C. saccharoperbutylacetonicum *and *C. sachharobutylicum *[5], biofuels such as ethanol and hydrogen - *Clostridium thermocellum *and *C. acetobutylicum *[6-9], indigo dye and flax retting to enzymes as therapeutic and cosmetic agents [10]. The potential of *Clostridium butyricum *to naturally produce 1,3-propanediol holds promise as base material for fiber - poly(propylene terapthalate) industry [11]. Cheese (Gonda and Grana) industry is invariably plagued by defects caused by *Clostridium tyrobutyricum*, *C. beijerinckii*, *C. butyricum *and *C. sporogenes *[12,13]. In contrast are the *Clostridium *strains with medical relevance particularly those with abilities to produce some of the deadliest toxins, leading to devastating clinical conditions [14-16]. Production of potent extracellular toxins by *Clostridium *spp. especially those due to *C. botulinum*, *C. perfringens*, *C. tetani *and *C. difficile *result in diseases such as botulism, gas gangrene, tetanus, pseudomembranous colitis and food-borne illness in man and animals [17-23]. To summarize, members of the *Clostridium *spp. are responsible for economic and medical problems [24].

Multiple methods have been employed for the identification of strains and/or samples of *Clostridium *and its species, such as PCR-ribotyping combined with DNA-DNA reassociation [25,26], Amplified fragment length polymorphism (AFLP) [17], Randomly amplified polymorphic DNA [27], Multilocus sequence typing [28], Restriction enzyme (RE) analysis [29,30] and Microarray [31].

### The heterogeneity of *Clostridium *species

#### *C. botulinum*

The heterogeneity of *C. botulinum *is evident from the variability of neurotoxins produced by them. Genetic studies reveal complex relationship between their taxonomy and toxin types, which needs a thorough re-examination. Certain non-toxigenic strains of *C. botulinum*, which have been genotypically shown to be authentic members of this species possess cryptic/silent genes or sequences coding for BoNT [32] but are functionally categorized as "non-producers". The complexity of the identification process is further enhanced by strains which phenotypically resemble *Clostridium baratii *and *C. butyricum *yet produce BoNT types F and E, respectively [33]. So these strains are referred to as *C. botulinum *in spite of the observations made on their DNA-DNA pairing hybridization, *rrs *sequencing and BoNT-encoding plasmids, etc. [16].

#### *C. acetobutylicum*

Different studies have provided evidences to support high genetic variability within *C. acetobutylicum *[34]. *C. acetobutylicum *is phylogenetically close to *C. felsineum *but DNA-DNA hybridization studies classify them as distinct species [34,35]. A genome wide comparison of *C. acetobutylicum *with *Bacillus subtilis *reveals significant local conservation of gene order [36]. Such high homology between proteins from other taxa emphasizes the potential of horizontal gene transfer, which leads to microbial evolution [16,36-38]. Interestingly, *B. subtilis *itself has been shown to be very heterogeneous [3,39]. Clostridial and *Bacillus *binary toxins share 80-85% identity within the *i *toxin family [40]. Phylogeny and evolutionary relationship within *Clostridium *are poorly understood even today [28].

### The needs of the genus *Clostridium*

*Clostridium *are phylogenetically extremely heterogeneous bacteria [35,41], with many non-spore formers grouped along with spore forming *Clostridium *spp. [42]. *Clostridium *contains both Gram-positive and Gram-negative species - *C. phytofermentans *shows a Gram-negative reaction in spite of possessing a Gram-positive cell wall ultrastructure [43]. In view of multiple hindrances encountered in accurately identifying these organisms it becomes difficult to establish a good co-relation between their pathogenic potential and disease manifestation [44]. The need is to have a reliable identification system [17] and to recognize distinct genetic-based clusters, which would help to establish a phenotype to genotype co-relation and lead to a clear taxonomic classification [45]. Since symptoms can be confusing, the need is to develop molecular genetics tools for improved diagnosis of *Clostridium *species [46]. Another, equaling challenging scenario is encountered due to their GC content, which varies from 24 mol% (*C. perfringens*) [47] to 58 mol% (*Clostridium barkeri*) [48]. Such a wide range of GC content is perhaps too great for a single genus [42] and may even demand re-classification.

A new dimension to establish evolutionary relationship among bacteria in general and specifically *Clostridium *species has been added by the availability of a large reservoir of *rrs *sequencing data http://rdp.cme.msu.edu/. Another important development has been the 1173 sequenced genomes http://www.ncbi.nlm.nih.gov/genomes/lproks.cgi including 224 Firmicutes consisting of 24 *Clostridium *spp. http://www.ncbi.nlm.nih.gov/genomes/lproks.cgi. All these put together are expected to prove helpful in redefining microbial taxonomy and phylogeny [3,4]. It may be remarked that studies based on *rrs *sequences need to be supplemented with information on other genes for authentic segregation of isolates [3]. Recent studies based on certain 'latent' features (unique signatures and *in silico *RE digestion patterns) of *rrs *alone could very clearly distinguish the two subgroups of *B. subtilis *[3], where as others have resorted to *gyrA *gene to elucidate this genetic variability [39]. It also proved instrumental in evaluating the biodiversity of *Stenotrophomonas *spp. [49]. The sequence of the 16S rDNA contains potential opportunities to characterize and differentiate species.

In this study, we have used *rrs *sequences to identify molecular markers, which may be sufficient enough to segregate *Clostridium *spp. Here, we have carried out comparative analyses of *rrs *sequences belonging to 15 *Clostridium *species as reference sets for generating species specific: (i) phylogenetic frameworks, (ii) signatures (30 nucleotides, nts) and (iii) *in silico *RE digestion patterns with 14 Type II REs. These tools were then used for identifying: (i) *Clostridium *sp. up to species level, (ii) identifying potential novel species, and (iii) tracking phylogenetic relationships among the other *Clostridium *species, which have fewer members at present.

## Results

The genus *Clostridium *has been reported (from RDP/NCBI sites: http://rdp.cme.msu.edu/; http://www.ncbi.nlm.nih.gov/ to consist of 110 species. For this study, the selected 765 *rrs *sequences (> 1200 nts) belonging to these 110 *Clostridium *species have been grouped into three categories (Table 1, Additional file 1: Table S1): (i) 15 *Clostridium *spp. with relatively higher number (8-128) of identified organisms, (ii) 94 *Clostridium *spp. (including 9 un-classified species and 5 species of *Eubacterium*) with relatively low frequency (1-15) of identified organisms and (iii) 179 organisms identified only up to genus level - *Clostridium *sp. Out of a total of 765 *rrs *sequences of different *Clostridium *species, we chose 404 for generating phylogenetic framework for the following 15 species belonging to the first group (Table 1): *C. botulinum *- 128, *C. perfringens *- 92, *C. butyricum *- 32, *C. acetobutylicum *- 24, *C. beijerinckii *- 23, *C. novyi *- 17, *C. kluyveri *- 14, *C. pasteurianum *- 13, *C. sporogenes *- 11, *C. colicanis *and *C. sardiniense *- 9 each, *C. baratii, C. chauvoei, C. subterminale *and *C. tetani *- 8 each. The species-specific phylogenetic framework developed here was used as a tool for (i) identifying 179 organisms *Clostridium *sp. up to genus level, and (ii) establishing the phylogenetic relationships among 182 organisms reported to be occur in low frequency (Additional file 1: Table S1).

**Table 1:** 16S rDNA sequences of *Clostridium *species which occurred with high frequency and number of sequences used in this study http://rdp.cme.msu.edu/

No.	Organism	No. of sequences
**1**.	*Clostridium botulinum*	128

**2**.	*C. perfringens*	92

**3**.	*C. butyricum*	32

**4**.	*C. acetobutylicum*	24

**5**.	*C. beijerinckii*	23

**6**.	*C. novyi*	17

**7**.	*C. kluyveri*	14

**8**.	*C. pasteurianum*	13

**9**.	*C. sporogenes*	11

**10**.	*C. colicanis*	9

**11**.	*C. sardiniense*	9

**12**.	*C. baratii*	8

**13**.	*C. chauvoei*	8

**14**.	*C. subterminale*	8

**15**.	*C. tetani*	8

**16.**	*Clostridium *spp. with low Frequency	182

**17**.	*Clostridium *sp.	179

Total		765

### Phylogenetic framework for *Clostridium *species

#### *C. botulinum*

Phylogenetic tree based on the *rrs *sequences of 128 strains of *C. botulinum *revealed their segregation into 4 major clusters (CBoI-CBoIV) (Additional file 2: Figure S1). These different clusters were represented by 3-83 strains. These four clusters may be representing 4 distinct lineages (I-IV) of *C. botulinum*, which have been segregated on the basis of neurotoxin (BoNTs) produced by them [20,50]. This segregation at this stage was supported by varied Bootstrap Value (BV): CBoI showed a BV in the range of 4-536 and exceptionally a value of 1000, indicating very high genetic variability among its members. Members of the cluster CBoII had BV in the range of 242-1000. A relatively low genetic heterogeneity was recorded among members of clusters CBoIII and CBoIV, where BV varied from 495-1000. In view of the high genetic variability among the different isolates, we selected four *rrs *sequences to represent CBoI, whereas two sequences each were selected for representing the rest three clusters: CBoII-CBoIV. In all, the 10 representative sequences including the type strains S000414699 and S000260209 were short listed. These *rrs *sequences were distributed all along the phylogenetic tree of *C. botulinum*. Further analyses based on nucleotide signatures and *in silico *RE digestion patterns supported the selection of these representatives.

#### *C. perfringens*

A phylogenetic tree based on the *rrs *sequences of 92 strains of *C. perfringens *showed 9 distinct clusters (CPeI-CPeIX) (Additional file 2: Figure S2) each consisting of between 3-20 strains. Four out of these 92 strains did not fell into any of these clusters. High genetic diversity among the different strains of *C. perfringens *was supported by low BV in each of the clusters. A total of ten representative sequences, including the one of the type strain (S000721508) were selected from 6 clusters (CPeI-CpeIV, CPeVI-CPeVII) for developing phylogenetic framework.

#### *C. butyricum*

The 32 different *rrs *sequences of *C. butyricum *were distributed largely on 6 different clusters (CBuI-CBuVI) (Additional file 2: Figure S3) of the phylogenetic tree. These different clusters were represented by 2-8 strains each. The low BV in different clades is a clear reflection on the high genetic heterogeneity within this *Clostridium *species. In order to represent the genetic heterogeneity of this *Clostridium *species in the phylogenetic framework, 3 *rrs *sequences were short listed (including one type strain (S000116309) from different clades - CBuII, CBuV and CBuVI.

#### *C. acetobutylicum*

The 24 strains of *C. acetobutylicum *were very heterogeneously distributed on their *rrs *sequence based phylogenetic tree. The two major clades (CAcI-CAcII) (Additional file 2: Figure S4) consisted of 9 and 5 sequences each, with a BV in the range of 393-1000 and 338-698, respectively. The rest 10 sequences were segregated into 9 different branches with a BV ranging from 7-369 and exceptionally it was 1000. The selection of five *rrs *sequences to develop a phylogenetic frame work was done in order to cover maximum range of the genetic diversity. Two of the selected strains (S000437209 and S000000299) represented the extreme ends of the phylogenetic tree, whereas the type strain S000628106 was located almost in its middle. A support to high genetic variability within *C. acetobutylicum *has been reported [34], with high potential for horizontal gene transfers [36].

#### *C. beijerinckii*

The 23 sequences of *rrs *of *C. beijerinckii *further supported the genetic heterogeneity within *Clostridium*. Four distinct clusters (CBeI-CBeIV) (Additional file 2: Figure S5) each containing 3-8 strains were represented by highly variable BVs: 197-1000, 95-135, 86-824, and 160-1000, respectively. Three sequences were selected to represent the diversity of the evolutionary tree: 2 from CBeI and the type strain (S000014607) from CBeIV.

#### *C. novyi*

Phylogenetic tree based on the *rrs *sequences from 17 strains of *C. novyi *showed 4 distinct clusters (CNoI-CNoIV) (Additional file 2: Figure S6) with 1-6 strains each. Only one strain (S000750034) was on an extreme end of these clusters. Four strains, including the type strain (S000016169) spanning the whole phylogenetic tree were taken into consideration while developing the framework.

#### *C. kluyveri*

A phylogenetic tree based on the *rrs *sequences of 14 strains of *C. kluyveri *formed 4 distinct clusters (CKlI-CKlIV) (Additional file 2: Figure S7) each containing at least 2 strains and a maximum of 6 strains. Two of the 14 strains, present almost at the extreme ends of the phylogenetic tree were selected for further use as framework sequences.

#### *C. pasteurianum*

The low genetic diversity among the 13 strains of *C. pasteurianum *was evident in their *rrs *sequence based phylogenetic tree. It showed a major cluster (CPaI) (Additional file 2: Figure S8) (containing 4 strains), whereas the rest of the sequences were distributed on independent branches with a maximum of two in a group. A total of three strains, including the type strain (S001792892), were selected for framework sequences.

#### *C. sporogenes*

Phylogenetic tree based on the *rrs *sequences from 11 strains of *C. sporogenes *formed a single group (Additional file 2: Figure S9). Two strains, including type strain (S000260539) and another from the extreme end of the tree were selected for framework sequences were taken to represent maximum diversity within this species.

#### *C. colicanis*

Phylogenetic tree based on the *rrs *sequences from 9 strains of *C. colicanis *showed no clear cut grouping as evident by the low BV (Additional file 2: Figure S10). Two strains, including type strain (S000366397), representing the two extreme ends of the phylogenetic tree were selected as framework sequences.

#### *C. sardiniense*

High genetic diversity in the phylogenetic tree based on the *rrs *sequences from 9 strains of *C. sardiniense *was supported by low BV in most of the branches (Additional file 2: Figure S11). Three strains, including type strain (S000539075), were selected as framework sequences to represent maximum genetic diversity within the species.

#### *C. baratii*

Phylogenetic tree based on the *rrs *sequences from 8 strains of *C. baratii *showed a single major group with high BV among most of the strains (Additional file 2: Figure S12). Three strains, including type strain (S000009597), were selected for framework sequences.

#### *C. chauvoei*

Phylogenetic tree based on the *rrs *sequences from 8 strains of *C. chauvoei *showed no clear cut grouping as reflected by the wide range of BV, which varied from 246-1000 (Additional file 2: Figure S13). Two strains, including type strain (S000437764), one from either end of the tree were selected for developing the phylogenetic framework.

#### *C. subterminale*

The genetic diversity within the *rrs *sequences of 8 strains of *C. subterminale *was quite low, as evident from the phylogenetic tree (CSuI-CSuII) (Additional file 2: Figure S14). Two representatives, one from each cluster, including the type strain (S000389872) were found to be sufficient for developing the phylogenetic frame work.

#### *C. tetani*

Phylogenetic tree based on the *rrs *sequences from 8 strains of *C. tetani *species showed three small groups (CTeI-CTeIII) (Additional file 2: Figure S15). Two strains, including type strain (S000260778), one from each end of the tree were selected for framework sequences to ensure inclusion of maximum diversity within the species in the study.

**Figure 1:**
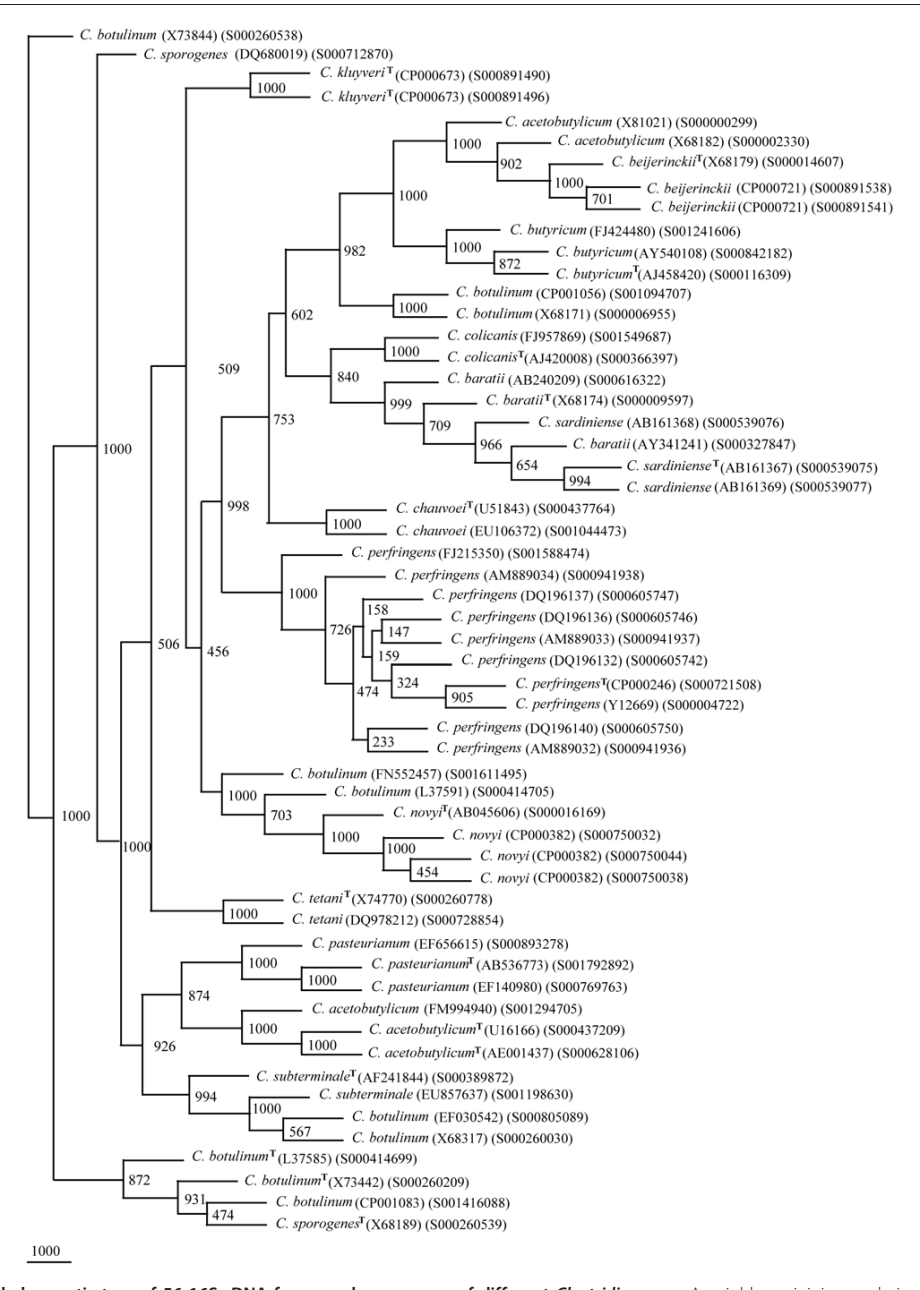
**Phylogenetic tree of 56 16S rDNA framework sequences of different *Clostridium *spp**. A neighbor - joining analysis with Jukes-Cantor correction and bootstrap support was performed on the *rrs *sequences belonging to *C. acetobutylicum*, *C. baratii, C. beijerinckii*, *Clostridium botulinum*, *C. butyricum*, *C. chauvoei, C. colicanis, C. kluyveri*, *C. novyi*, *C. pasteurianum*, *C. perfringens*, *C. sardiniense*, *C. sporogenes*, *C. subterminale*, and *C. tetani*. Bootstrap values are given at nodes. Values in parentheses are accession numbers (RDP and NCBI) (http://rdp.cme.msu.edu/ and http://www.ncbi.nlm.nih.gov/).

A total of 56 *rrs *sequences were short listed to represent the genetic diversity in 15 different *Clostridium *species (Table 2). A reference phylogenetic tree based on framework sequences (Figure 1) shows clear cut segregation of *rrs *sequences into separate clades, each consisting of members of *C. butyricum*, *C. chauvoei*, *C. colicanis*, *C. kluyveri*, *C. perfringens *and *C. tetani*. In contrast, *C. acetobutylicum *was found to be segregated into two groups (i) one close to *C. pasteurianum *(BV 874) and (ii) another showing high similarity with *C. beijerinckii *and *C. butyricum*. High heterogeneity was observed between *C. baratii *and *C. sardiniense*. The most heterogeneous group was represented by the strains of *C. botulinum*. Four different associations were recorded between *C. botulinum *and (i) *C. sporogenes*, (ii) *C. subterminale*, (iii) *C. novyi *and (iv) a group of *C. acetobutylicum *and *C. butyricum *The most interesting feature of the reference phylogenetic framework tree were (i) completely independent placing of *C. chauvoei, C. kluyveri, C. tetani *and (ii) close relationships among (a) *C. acetobutylicum*, *C. beijerinckii*, *C. botulinum *and *C. butyricum *(BV 982-1000), (b) *C. baratii*, *C. colicanis *and *C. sardiniense *(BV 840-1000), (c) *C. botulinum *and *C. novyi *(BV 703-1000), and (d) *C. acetobutylicum*, *C. botulinum, C. pasteurianum *and *C. subterminale *(BV 874-926).

**Table 2:** Accession numbers of 16S rDNA sequences of *Clostridium *species used for generating phylogenetic framework (http://www.ncbi.nlm.nih.gov/ and http://rdp.cme.msu.edu/)

Organism	Reference sequence(s)
*Clostridium acetobutylicum*	U16166(T)^a^, AE001437(T), FM994940, X68182, X81021

*C. baratii*	X68174(T), AB240209, AY341241

*C. beijerinckii*	X68179(T), CP000721 (S000891541)^b^, CP000721 (S000891538)

*C. botulinum*	L37585(T), X73442(T), EF030542, L37591, CP001056, X73844, FN552457, X68171, X68317, CP001083

*C. butyricum*	AJ458420(T), FJ424480, AY540108

*C. chauvoei*	U51843(T), EU106372

*C. colicanis*	AJ420008(T), FJ957869

*C. kluyveri*	CP000673(T) (S000891496), CP000673(T) (S000891490)

*C. novyi*	AB045606(T), CP000382 (S000750038), CP000382 (S000750032), CP000382 (S000750044)

*C. pasteurianum*	EF656615, AB536773, EF140980

*C. perfringens*	CP000246(T), DQ196140, AM889033, AM889032, DQ196137, DQ196132, DQ196136, Y12669, AM889034, FJ215350

*C. sardiniense*	AB161367(T), AB161368, AB161369

*C. sporogenes*	X68189(T), DQ680019

*C. subterminale*	AF241844(T), EU857637

*C. tetani*	X74770(T), DQ978212

**Total**	**56 strains**

^a^Type strain

^b^NCBI sequence entries with multiple accession numbers in RDP data bases have been mentioned within the parentheses

### Validation of species-specific phylogenetic framework sequences

**Figure 2:**
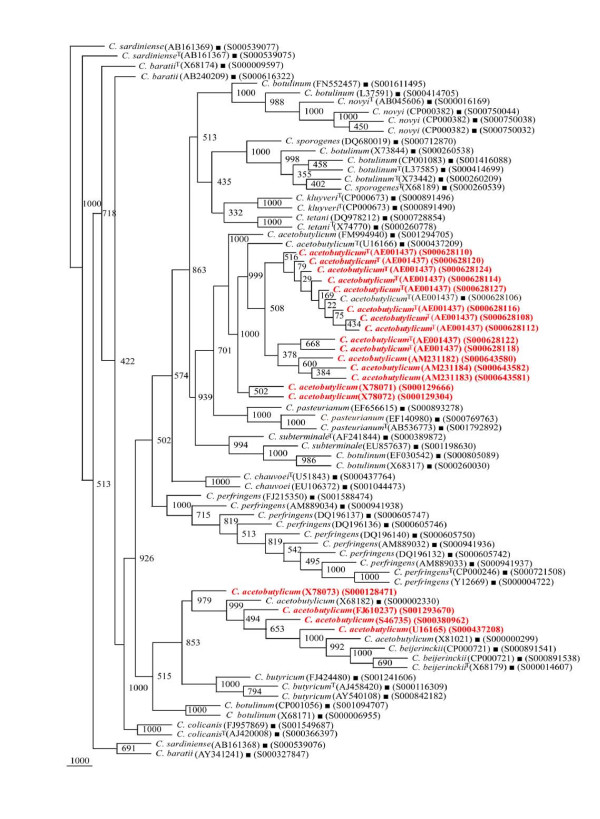
**Phylogenetic tree of 16S rDNA of *Clostridium acetobutylicum *and Framework sequences**. A neighbor - joining analysis with Jukes-Cantor correction and bootstrap support was performed on the *rrs *sequences of *C. acetobutylicum *- 24 (shown in red, except those used as framework sequences) along with 56 of phylogenetic framework (Figure 1). Bootstrap values are given at nodes. Sequences marked by filled square are the ones considered as framework in the study whereas type strains are indicated by 'T' as superscript. Values in parentheses are accession numbers (RDP and NCBI) (http://rdp.cme.msu.edu/ and http://www.ncbi.nlm.nih.gov/).

**Figure 3:**
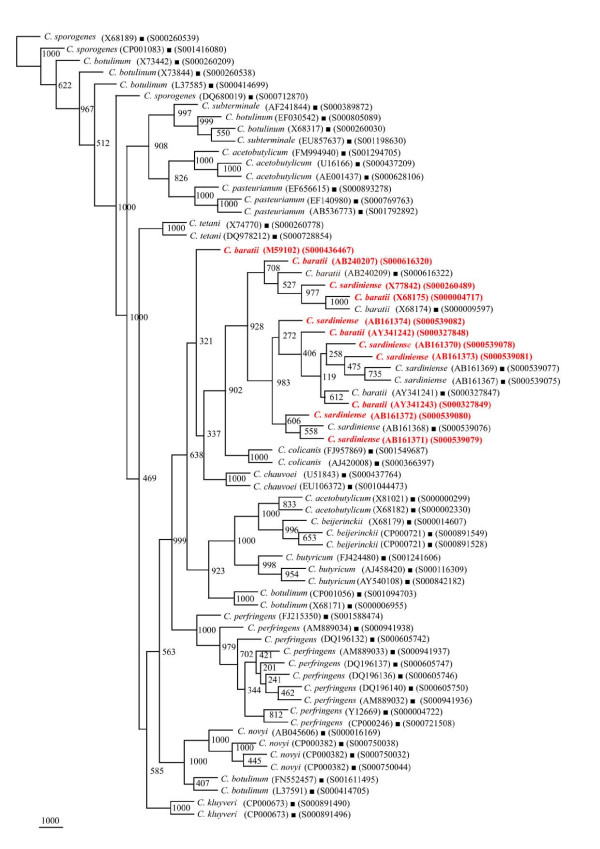
**Phylogenetic tree of 16S rDNA of *Clostridium baratii, C. sardiniense *and Framework sequences**. A neighbor - joining analysis with Jukes-Cantor correction and bootstrap support was performed on the *rrs *sequences of *C. baratii *- 8, *C. sardiniense *- 9 (shown in red, except those used as framework sequences) along with 56 of phylogenetic framework (Figure 1). Bootstrap values are given at nodes. Sequences marked by filled square are the ones considered as framework in the study whereas type strains are indicated by 'T' as superscript. Values in parentheses are accession numbers (RDP and NCBI) (http://rdp.cme.msu.edu/ and http://www.ncbi.nlm.nih.gov/).

**Figure 4:**
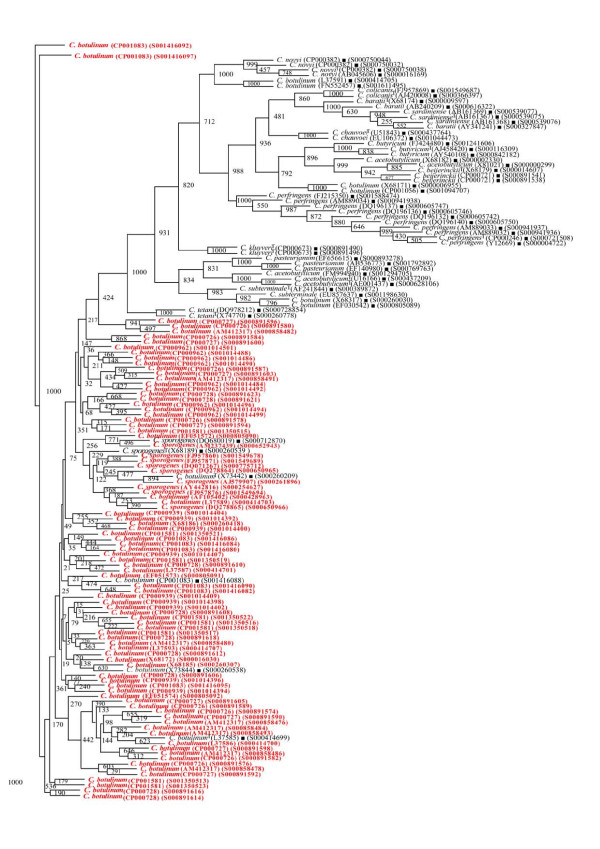
**Phylogenetic tree of 16S rDNA of *Clostridium botulinum, C. sporogenes *and Framework sequences**. A neighbor - joining analysis with Jukes-Cantor correction and bootstrap support was performed on the *rrs *sequences of *C. botulinum *- 83, *C. sporogenes *- 11 (shown in red, except those used as framework sequences) along with 56 of phylogenetic framework (Figure 1). Bootstrap values are given at nodes. Sequences marked by filled square are the ones considered as framework in the study whereas type strains are indicated by 'T' as superscript. Values in parentheses are accession numbers (RDP and NCBI) (http://rdp.cme.msu.edu/ and http://www.ncbi.nlm.nih.gov/).

**Figure 5:**
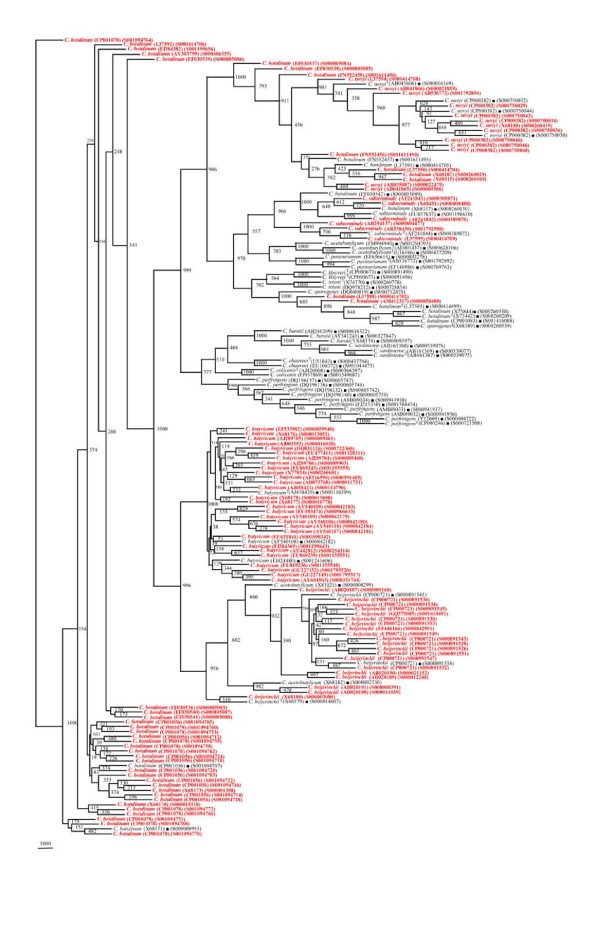
**Phylogenetic tree of 16S rDNA of 5 *Clostridium *species and Framework sequences**. A neighbor - joining analysis with Jukes-Cantor correction and bootstrap support was performed on the *rrs *sequences of *C. botulinum *- 45, *C. butyricum *- 32, *C. novyi*, - 17, *C. beijerinckii *- 23, *C. subterminale *- 8 (shown in red, except those used as framework sequences) along with 56 of phylogenetic framework (Figure 1). Bootstrap values are given at nodes. Sequences marked by filled square are the ones considered as framework in the study whereas type strains are indicated by 'T' as superscript. Values in parentheses are accession numbers (RDP and NCBI) (http://rdp.cme.msu.edu/ and http://www.ncbi.nlm.nih.gov/).

**Figure 6:**
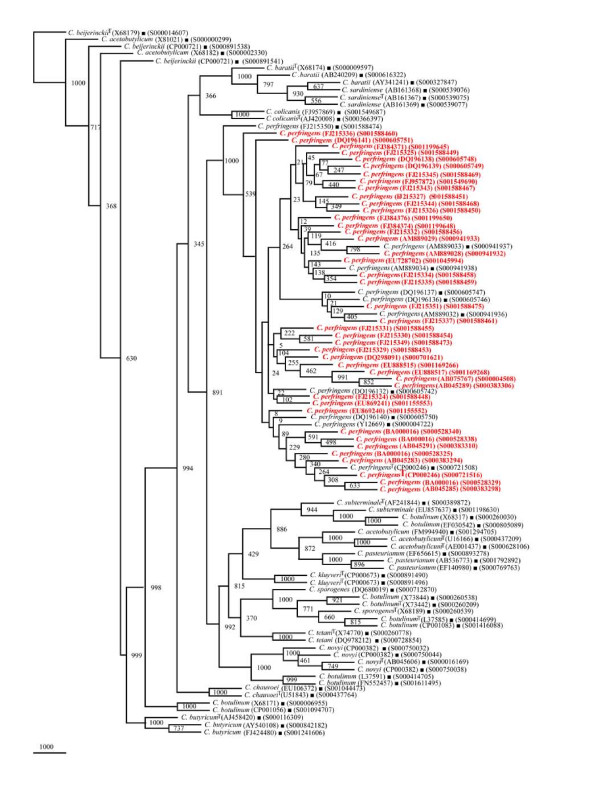
**Phylogenetic tree of 16S rDNA of *Clostridium perfringens *and Framework sequences**. A neighbor - joining analysis with Jukes-Cantor correction and bootstrap support was performed on the *rrs *sequences of *C. perfringens *- 46 (shown in red, except those used as framework sequences) along with 56 of phylogenetic framework (Figure 1). Bootstrap values are given at nodes. Sequences marked by filled square are the ones considered as framework in the study whereas type strains are indicated by 'T' as superscript. Values in parentheses are accession numbers (RDP and NCBI) (http://rdp.cme.msu.edu/ and http://www.ncbi.nlm.nih.gov/). Note: Out of a total of 92 *C. perfringens rrs *sequences, 46 have been presented here to achieve clarity of presentation. The rest 46 *rrs *sequences have been presented in Figure 7.

**Figure 7:**
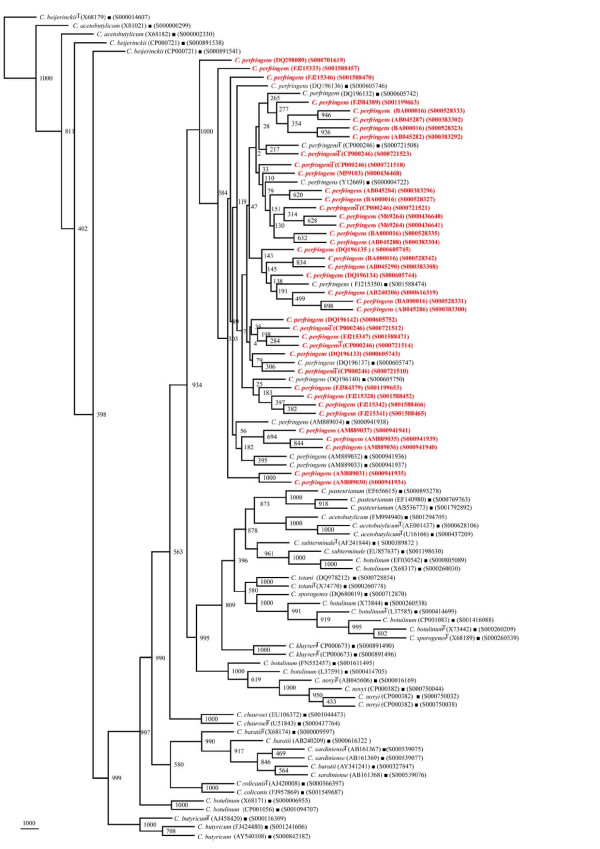
**Phylogenetic tree of 16S rDNA of rest *Clostridium perfringens *and Framework sequences**. A neighbor - joining analysis with Jukes-Cantor correction and bootstrap support was performed on the *rrs *sequences of *C. perfringens *- 46 (shown in red, except those used as framework sequences) along with 56 of phylogenetic framework (Figure 1). Bootstrap values are given at nodes. Sequences marked by filled square are the ones considered as framework in the study whereas type strains are indicated by 'T' as superscript. Values in parentheses are accession numbers (RDP and NCBI) (http://rdp.cme.msu.edu/ and http://www.ncbi.nlm.nih.gov/). Note: Out of a total of 92 *C. perfringens rrs *sequences, 46 have been presented here to achieve clarity of presentation. The first 46 *rrs *sequences have been presented in Figure 6.

**Figure 8:**
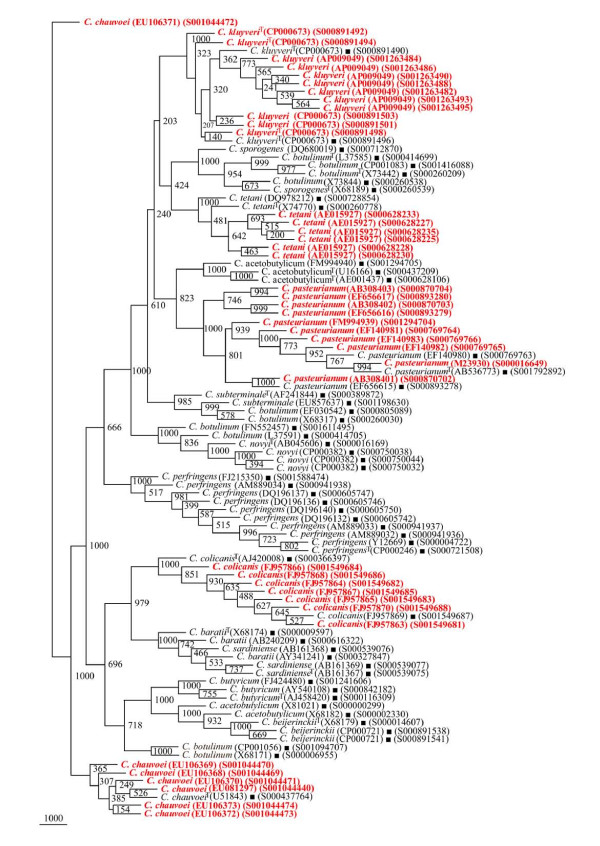
**Phylogenetic tree of 16S rDNA of 5 different *Clostridium *species and Framework sequences**. A neighbor - joining analysis with Jukes-Cantor correction and bootstrap support was performed on the *rrs *sequences of *Clostridium kluyveri *- 14, *C. pasteurianum *- 13, *C. colicanis *- 9, *C. chauvoei *and *C. tetani *- 8 each (shown in red, except those used as framework sequences) along with 56 of phylogenetic framework (Figure 1). Bootstrap values are given at nodes. Sequences marked by filled square are the ones considered as framework in the study whereas type strains are indicated by 'T' as superscript. Values in parentheses are accession numbers (RDP and NCBI) (http://rdp.cme.msu.edu/ and http://www.ncbi.nlm.nih.gov/).

In our quest to validate the phylogenetic framework sequences, different phylogenetic trees were drawn on the basis of 404 *rrs *sequences belonging to 15 different *Clostridium *species along with 56 reference sequences. Seven different phylogenetic trees (Figures 2, 3, 4, 5, 6, 7 and 8) very clearly showed that the *Clostridium *isolates segregated well within their respective phylogenetic framework sequences except *C. acetobutylicum *(Figure 2). In the case of *C. acetobutylicum *eighteen *rrs *sequences segregated well with the type strain (S000628106) and 2 framework sequences and the rest six (including two frame work sequences) formed an independent group, which was quite close to *C. beijerinckii *along with *C. butyricum*.

#### (i) Phylogenetic relationships of *Clostridium *sp

**Figure 9:**
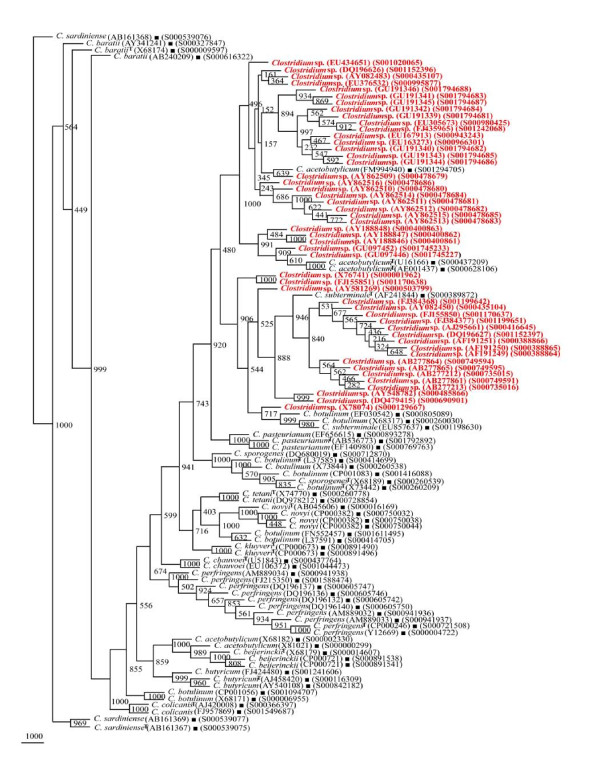
**Phylogenetic tree of 16S rDNA of *Clostridium *sp. and Framework sequences**. A neighbor - joining analysis with Jukes-Cantor correction and bootstrap support was performed on the *rrs *sequences of *Clostridium *sp. - 49 (the different isolates could be segregated as *C. acetobutylicum *- 29 and *C. subterminale *- 20) along with 56 of phylogenetic framework (Figure 1). Bootstrap values are given at nodes. Sequences marked by filled square are the ones considered as framework in the study whereas type strains are indicated by 'T' as superscript. Values in parentheses are accession numbers (RDP and NCBI) (http://rdp.cme.msu.edu/ and http://www.ncbi.nlm.nih.gov/). Note: Out of a total of 95 *Clostridium *sp. *rrs *sequences, 49 have been presented here to achieve clarity of presentation and are shown in red. The rest 46 *rrs *sequences have been presented in Figure 10.

**Figure 10:**
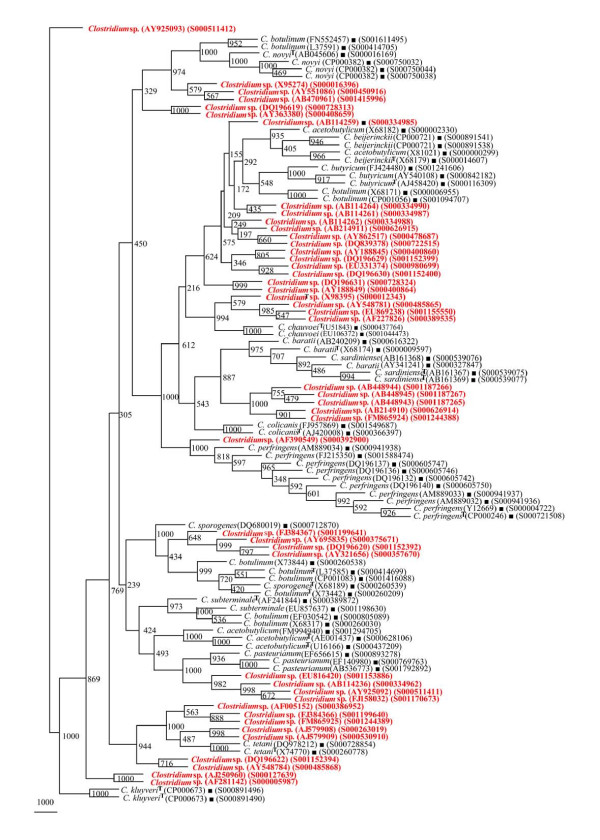
**Phylogenetic tree of 16S rDNA of rest *Clostridium *sp. and Framework sequences**. A neighbor - joining analysis with Jukes-Cantor correction and bootstrap support was performed on the *rrs *sequences of *Clostridium *sp. - 46 (the different that isolates could be segregated as *C. tetani *- 7, *C. baratii *- 5, *C. chauvoei *and *C. pasteurianum *- 4 each, *C. kluyveri *- 3, *C. perfringens *- 1, *C. acetobutylicum*/*C. beijerinckii *- 13 and *C. botulinum*/*C. novyi/C. sporogenes *- 9) along with 56 of phylogenetic framework (Figure 1). Bootstrap values are given at nodes. Sequences marked by filled square are the ones considered as framework in the study whereas type strains are indicated by 'T' as superscript. Values in parentheses are accession numbers (RDP and NCBI) (http://rdp.cme.msu.edu/ and http://www.ncbi.nlm.nih.gov/). Note: Out of a total of 95 *Clostridium *sp. *rrs *sequences, 46 have been presented here to achieve clarity of presentation and are shown in red. The first 49 *rrs *sequences have been presented in Figure 9.

**Figure 11:**
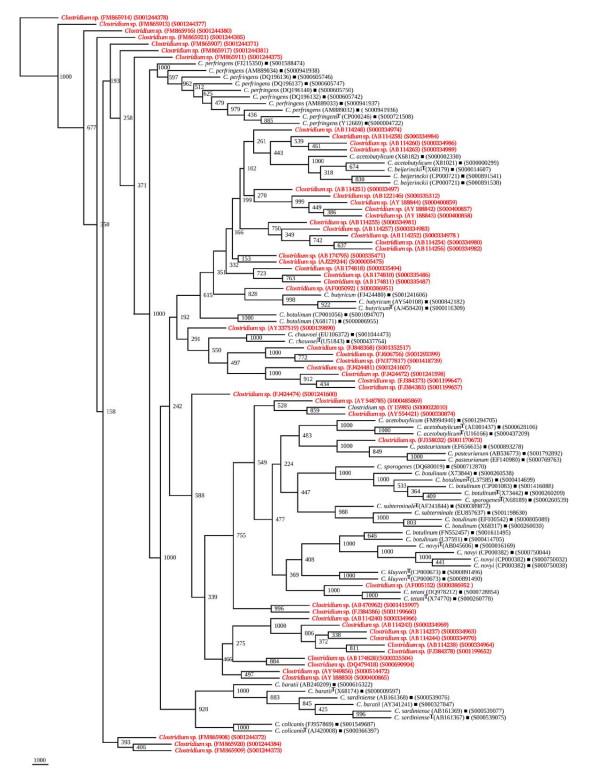
**Phylogenetic tree of 16S rDNA of novel *Clostridium *sp. and Framework sequences**. A neighbor - joining analysis with Jukes-Cantor correction and bootstrap support was performed on the *rrs *sequences of novel *Clostridium *sp. - 84 (shown in red) (as these could not be segregated among the 15 *Clostridium *sp. known to occur at high frequency and rest of the *Clostridium *spp. known to occur with low frequency (Additional file 6: Figures S18 and S19) along with 56 of phylogenetic framework (Figure 1). Bootstrap values are given at nodes. Sequences marked by filled square are the ones considered as framework in the study whereas type strains are indicated by 'T' as superscript. Values in parentheses are accession numbers (RDP and NCBI) (http://rdp.cme.msu.edu/ and http://www.ncbi.nlm.nih.gov/).

The phylogenetic framework of *rrs *sequences described above was used to check if 179 isolates identified as *Clostridium *sp. can be classified among these *Clostridium *species reported to occur with high frequency (Figures 9, 10 and 11). Out of these 179 *Clostridium *sp., 95 were found to segregate among 15 *Clostridium *species with a high BV (Figures 9 and 10, Table 3): *C. acetobutylicum *- 29, *C. subterminale *- 20 (Figure 9), *C. tetani *- 7, *C. baratii *and *C. botulinum *- 5 each, *C. sporogenes/C. botulinum *and *C. chauvoei *- 4 each, *C. pasteurianum *and *C. kluyveri *- 3 each, *C. botulinum*/*C. butyricum *- 2 and *C. perfringens *- 1 (Figure 10). A unique case of 11 *Clostridium *sp. was their association with *C. acetobutylicum *and *C. beijerinckii *albeit with low BV of 350-460 (Figure 10). However, subsequent analyses (signatures and *in silico *RE digestion pattern) revealed that these 11 *Clostridium *species can be classified as *C. beijerinckii*. Incidentally, none of the *Clostridium *sp. was found to belong to *C. sardiniense*, *C. novyi *and *C. colicanis*. A group of 84 *rrs *sequences of *Clostridium *sp. could not be grouped among the 15 known *Clostridium *species (Figure 11). Since these 84 isolates have the potential to be classified as novel *Clostridium *species, these were also checked for their phylogenetic relationships with *Clostridium *spp. with low frequency of occurrence.

**Table 3:** Accession numbers of 16S rDNA sequences of *Clostridium *spp. identified up to species level. http://rdp.cme.msu.edu/

Strains close to Framework organisms^a ^of different *Clostridium *spp.	Total No.
***C. acetobutylicum***	

S000400861, S000400862, S000400863, S000435107, S000478679, S000478680, S000478681, S000478682, S000478683, S000478684, S000478685, S000478686, S000943243, S000966301, S000980425, S000995877, S001020065, S001152396, S001242068, S001745227, S001745233, S001794681, S001794682, S001794683, S001794684, S001794685, S001794686, S001794687, S001794688	29

***C. beijerinckii/C. acetobutylicum***	

S000334985, S000334987, S000334988, S000334990, S000400860, S000400864, S000478687, S000626915, S000722515, S000728324, S000980699, S001152399, S001152400	13

***C. subterminale***	

S000001962, S000129667, S000388864, S000388865, S000388866, S000416645, S000435104, S000485866, S000503799, S000690901, S000735015, S000735016, S000749591, S000749594, S000749595, S001152397, S001170637, S001170638, S001199642, S001199651	20

***C. botulinum/C. novyi/C. Sporogenes***	

S000016396, S000357670, S000375671, S000408659, S000450916, S000728313, S001152392, S001199641, S001415996	9

***C. tetani***	

S000263019, S000386952, S000485868, S000530910, S001152394, S001199640, S001244389	7

***C. chauvoei***	

S000012343, S000389535, S000485865, S001155550	4
***C. kluyveri***	

S000005987, S000127639, S000511412	3

***C. pasteurianum***	

S000334962, S000511411, S001153886, S001170673	4

***C. baratii***	
S000626914 (Close to *C. sardiniense*), S001244388, S001187265, S001187266, S001187267	5

***C. perfringens***	

S000392900	1

Total	95

No *Clostridium *sp. could be assigned to *C. butyricum, C. colicanis *and *C. sardiniense*.

^a ^For Framework organisms see Table 2.

#### (ii) Phylogenetic relationships of *Clostridium *species with low frequency occurrence

**Figure 12:**
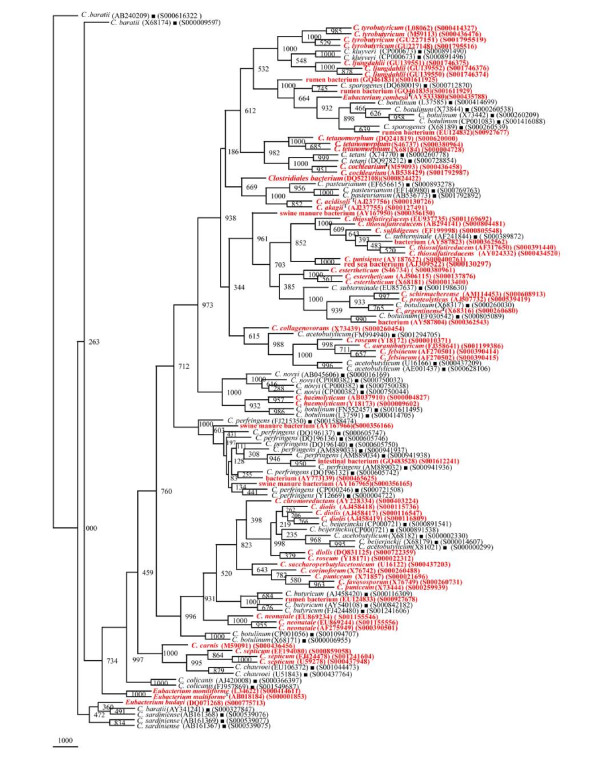
**Phylogenetic tree of 16S rDNA of low frequency *Clostridium *sp. (segregated) and Framework sequences**. A neighbor - joining analysis with Jukes-Cantor correction and bootstrap support was performed on the *rrs *sequences of low frequency *Clostridium *sp. - 68 (shown in red) (which could be segregated among the 15 known *Clostridium *sp. known to occur at high frequency) along with 56 of phylogenetic framework (Figure 1). Bootstrap values are given at nodes. Sequences marked by filled square are the ones considered as framework in the study whereas type strains are indicated by 'T' as superscript. Values in parentheses are accession numbers (RDP and NCBI) (http://rdp.cme.msu.edu/ and http://www.ncbi.nlm.nih.gov/).

**Figure 13:**
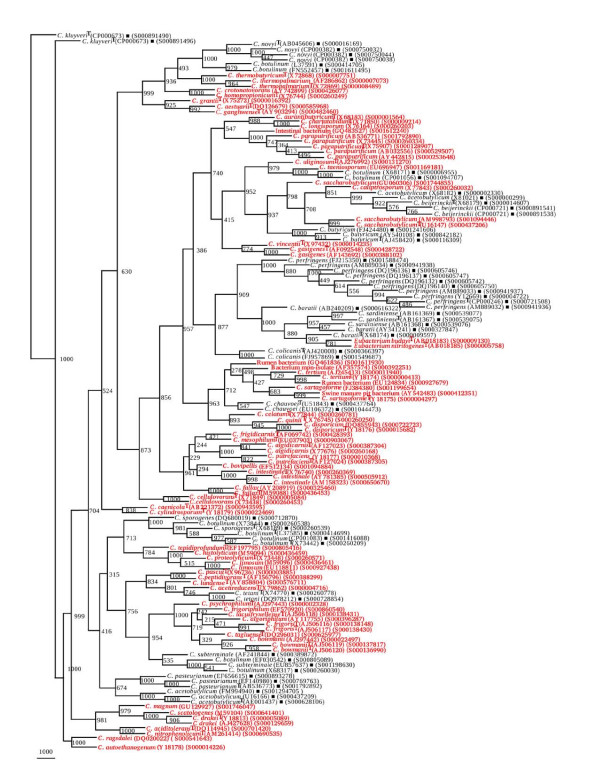
**Phylogenetic tree of 16S rDNA of low frequency *Clostridium *sp. (unsegregated) and Framework sequences**. A neighbor - joining analysis with Jukes-Cantor correction and bootstrap support was performed on the *rrs *sequences of low frequency *Clostridium *sp. - 83 (shown in red) (which could not be segregated among the 15 known *Clostridium *sp. known to occur at high frequency) along with 56 of phylogenetic framework (Figure 1). Bootstrap values are given at nodes. Sequences marked by filled square are the ones considered as framework in the study whereas type strains are indicated by 'T' as superscript. Values in parentheses are accession numbers (RDP and NCBI) (http://rdp.cme.msu.edu/ and http://www.ncbi.nlm.nih.gov/).

A total of 182 *rrs *sequences of *Clostridium *isolates belonging to 80 distinct species and 7 groups which have been classified as swine manure, intestinal bacterium, *Eubacterium*, etc. (Additional file 1: Table S1) were found to form distinct phylogenetic groups (Figures 12 and 13, Additional file 3: Figure S16). The evolutionary relationship among the 68 *rrs *sequences of *Clostridium *spp. (Table 4) which have been reported so far to occur with a low frequency formed a few very distinct and independent groups along with those occurring with relatively high frequency, for example - *C. subterminale *and *C. estertheticum*. Although these appear to be close on a single clade but are phylogenetically quite distant as evident by low BV 385. Another clade, which harbored *C. subterminale *was associated quite closely (BV 703) with *Clostridium tunisiense*, and red sea bacterium on one clade, *Clostridium sulfidigenes*, *C. thiosulfatireducens *and bacterium isolates (BV 852-1000) on a second clade and yet another branch carried swine manure bacterium (BV 961). The two species - *C. acetobutylicum *and *C. beijerinckii *reported to occur with high frequency were weakly grouped along with *Clostridium diolis *(BV 219), *C. chromoreductans *(BV 398), and *C. corinoforum *and *C. saccharoperbutylacetonicum *(BV 643), but showed strong association with *C. roseum *(BV 998), *C. puniceum *and *C. favososporum *(BV 823), (Figure 12). These diverse *Clostridium *species are quite heterogeneous and reflect their individual identities. However, their grouping together indicates the possibility of their common origin, which was quite high in the case of *C. kluyveri *branching with *C. tyrobutyricum *(BV 1000) but moderate in the case of *Clostridium ljungdahlii *(BV 548). *C. botulinum*, which is known to be genetically quite heterogeneous, showed close relationship with *Eubacterium *and rumen bacterium (BV 664-932) on one hand and with *C. neonatale *on the other (BV 996). Similarly, *C. acetobutylicum *showed strong association with *Clostridium aurantibutyricum*, *C. felsineum *and *C. roseum *(BV 998-1000). It also branched along with *Clostridium collagenovorans *albeit with a relatively low BV of 615. *C. botulinum *and *C. novyi *shared a branch on the phylogenetic tree along with *C. haemolyticum *(BV 932-1000). *C. tetani *is a unique *Clostridium *sp. which appears to be of independent origin; however, it showed closeness to *Clostridium cochlearium *and *C. tetanomorphum *(BV 982-1000). *C. chauvoei *was found to be close to *Clostridium carnis *and *C. septicum *(BV 995-1000), whereas *C. pasteurianum *showed high homology with *C. acidisoli *and *C. akagii *(BV 1000) and less homology with Clostridiales in general (BV 670) (Figure 12). Thirty one *rrs *sequences of *Clostridium *isolates belonging to swine fecal, unidentified eubacterium and clostrideaceae bacterium (Additional file 1: Table S1), which did not cluster with *Clostridium *spp., were found to have an overall low phylogenetic relationship (Additional file 3: Figure S16). The evolutionary relationship among the rest 83 of 182 *rrs *sequences of *Clostridium *spp. which have been reported so far to occur with a low frequency formed a few very distinct and independent groups (Figure 13).

**Table 4:** Phylogenetic relationships of *Clostridium *spp. (low frequency) with Frame work organisms supported by RE patterns and signatures^a^

*Clostridium *spp.	Ribosomal Database Acc. No.	RE^b^	Nucleotide signatures (Motif, M)									
			**1**	**2**	**3**	**4**	**5**	**6**	**7**	**8**	**9**	**10**

*Clostridium *sp. close to Framework sequences of *C. subterminale*												

*C. thiosulfatireducens*	S000804481, S000434520, S000391440	DpnII	+^c^	+	+	+	+	U^d^	+	+	+	+
	
	S001169692	DpnII	+	+	+	+	+	U	+	+	+	- ^c^

*C. argentiense*	S000260680	DpnII	+	+	+	+	+	U	+	+	+	+

*C. schirmacherense*	S000608913	DpnII	+	+	+	+	+	-	+	+	+	+

*C. proteolyticus*	S000539419	DpnII	+	+	-	+	+	-	+	+	+	+

*C. tunisiense*	S000400761	DpnII	+	+	+	+	+	-	+	-	+	+

*C. sulfidigens*	S000805548	DpnII	+	+	+	+	+	U	+	+	+	+

swine manure bacterium	S000356150	DpnII	+	+	-	+	+	U	+	+	+	+

red sea bacterium	S000130297	DpnII	+	+	+	+	+	-	+	+	+	+

bacterium	S000362562	DpnII	+	+	+	+	+	U	+	+	+	-

	S000362543	No Match	+	+	+	+	+	U	+	+	+	-

*C. estertheticum*	S000380961, S000137876, S000013400	No Match	+	+	-	-	+	-	+	+	+	+

*Clostridium *sp. close to Framework sequences of *C. beijerinckii*												

*C. diolis*	S000116547, S000116809, S000115736	Tru9I	+	+	+	+	+	+	+	+	+	-

	S000722359	Tru9I	+	+	+	+	+	-	+	+	+	-

*C. saccharoperbutylacetonicum*	S000437203	Tru9I	+	+	+	+	+	-	+	+	+	-

*C. roseum*	S000022312	Tru9I	+	+	+	+	+	+	+	+	+	-

*C. corinoforum*	S000260488	Tru9I	+	+	+	+	+	+	+	+	+	-

*C. chromoreductans*	S000403224	Tru9I	+	+	+	+	+	-	+	+	+	-

*C. favososporum*	S000260731	Tru9I	+	+	+	+	+	-	+	+	+	-

*C. puniceum*	S000259939	No Match	+	+	+	+	-	-	+	+	+	-

	S000021696	No Match	+	+	+	+	+	-	+	+	+	-

*Clostridium *sp. close to Framework sequences of *C. kluyveri*												

*C. tyrobutyricum*	S001795516, S000414327	BfaI	+	-	+	-	-	-	+	+	-	+

	S001795519, S000436476	BfaI	-	-	+	-	-	-	-	-	-	-

*C. ljungdahlii*	S001746374, S001746375, S001746376	BfaI	+	-	+	-	-	+	+	+	-	-

*Clostridium *sp. close to Framework sequences of *C. tetani*												

*C. tetanomorphum*	S000620000, S000380964, S000004728	HaeIII	-	-	-	-	-	-	-	-	-	+

*C. cochlearium*	S000436458, S001792987	HaeIII	U	-	-	U	U	U	-	U	U	-

*Clostridium *sp. close to Framework sequences of *C. chauvoei*												

*C. septicum*	S000437948, S001241604	AluI	+	+	U	-	-	+	+	-	U	+

	S000859058	AluI	-	+	U	-	-	+	+	-	U	+

*C. carnis*	S000436456	AluI	U	+	U	U	-	+	-	-	U	+

*Clostridium *sp. close to Framework sequences of *C. perfringens*												

swine manure bacterium	S000356165, S000356166	BfaI, HaeIII	U	U	U	U	-	+	**+**	**+**	**+**	**+**

bacterium	S000465625	BfaI, HaeIII	U	U	U	U	U	+	-	+	+	+

intestinal bacterium	S001612241	BfaI, HaeIII	U	U	U	U	U	+	**+**	+	+	+

*Clostridium *sp. close to Framework sequences of *C. butyricum*												

rumen bacterium	S000927678		+	+	+	+	+	+	+	+	+	+

*Clostridium *sp. close to Framework sequences of *C. pasteurianum*												

*C. acidisoli*	S000130726	BfaI	-	-	U	+	U	-	U	U	+	+

*C. akagii*	S000127491	BfaI	-	-	-	+	-	-	U	U	+	+

*Clostridiales bacterium*	S000824422	BfaI	-	-	-	-	-	-	-	-	+	+

*Clostridium *sp. close to Framework sequences of *C. novyi/C. botulinum*												

*C. haemolyticum*	S000004827, S000009602	Tru9I	U	+	U	+	+	U	+	+	+	U

*Clostridium *sp. close to Framework sequences of *C. baratii*												

*Eubacterium monoliforme*	S000414611	No Match	+	-	U	+	+	-	+	-	+	+

*Eubacterium multiforme*	S000001853	No Match	-	-	-	+	+	-	+	+	+	+

*Clostridium *sp. close to Framework sequences of *C. sporogenes/C. botulinum*												

rumen bacterium	S001611925, S00927677	BfaI, Tru9I	-	-	+	-	+	+	+	+	-	-

*Clostridium *sp. close to Framework sequences of *C. sardiniense*												

*Eubacterium budayi*	S000775713	Tru9I	-	U	+	U	-	-	-	-	-	-

*Clostridium *sp. close to Framework sequences of *C. botulinum*												

*C. neonatale*	S001155546, S001155556, S000390501	Tru9I, BfaI	+	+	+	+	+	+	+	+	+	+

*Eubacterium combesii*	S000435788	Tru9I, BfaI	+	+	+	+	+	+	+	+	+	+

rumen bacterium	S001611929	Tru9I, BfaI	+	+	+	+	+	+	+	-	+	+

*Clostridium *sp. close to Framework sequences of *C. acetobutylicum*												

*C. roseum*	S000010371	AluI	+	+	+	+	+	+	+	+	+	+

*C. felsineum*	S000390414, S000390415	AluI	+	+	+	+	+	+	+	+	+	+

*C. aurantibutyricum*	S001199386	AluI	+	+	+	+	+	+	+	+	+	+

*C. collagenovorans*	S000260454	AluI	+	+	+	+	+	+	+	+	+	+

^a,b,d^*Clostridium *spp. known to occur with low frequency were found to be phylogentically close to *Clostridium *spp. used for developing Framework (Table 2) were also similar with respect to their *in silico *RE^b ^digestion pattern and unique^c ^nucleotide signatures (30 nts in lengths) (Table 6) ^c^+/- denotes presence/absence of a nucleotide signature also found in the respective species represented in the phylogenetic framework.

#### (iii) Phylogenetic relationships of novel *Clostridium *species

Out of 179 isolates of *Clostridium *sp., 84 *rrs *sequences which have the potential to be classified as novel were found to be represented by 56 groups (Additional file 4: Table S2). A quite low BVs among them implies high genetic heterogeneity (Additional file 5: Figure S17). To further verify if these 84 *Clostridium *sp. are of independent origin, their phylogenetic relationships were checked with *Clostridium *spp. reported to occur with low frequency (Additional file 4: Table S3). It can be seen from the phylogenetic trees (Additional file 6: Figures S18 and S19) that they are quite independent as supported by low BV. The possibility of assigning these 84 *rrs *sequences to novel *Clostridium *species was further supported by subsequent analyses - nucleotide signatures and *in silico *RE digestion patterns.

### *In silico *restriction enzyme activity

**Figure 14:**
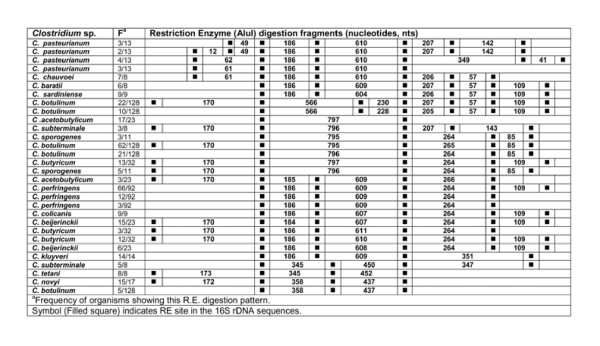
***In silico *Restriction Enzyme activity in 16S rDNA sequences of *Clostridium *spp.: AluI**. ^a^Frequency of organisms showing this RE digestion pattern. Symbol (Filled square) indicates RE site in the *rrs *sequences.

**Figure 15:**
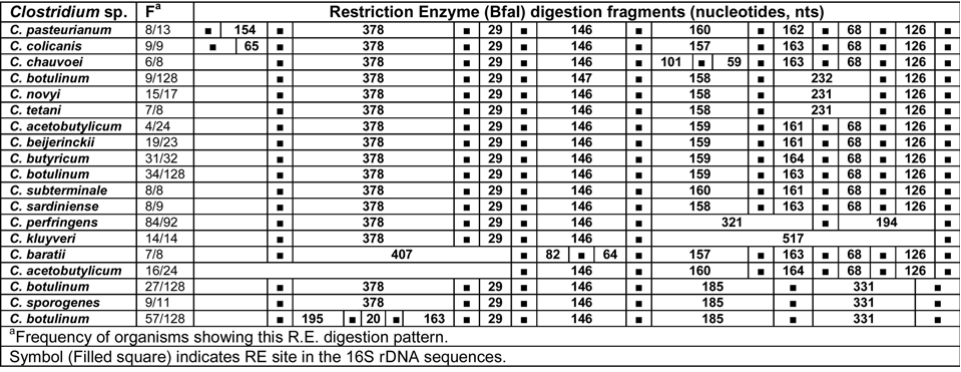
***In silico *Restriction Enzyme activity in 16S rDNA sequences of *Clostridium *spp.: BfaI**. ^a^Frequency of organisms showing this RE digestion pattern. Symbol (Filled square) indicates RE site in the *rrs *sequences.

**Figure 16:**
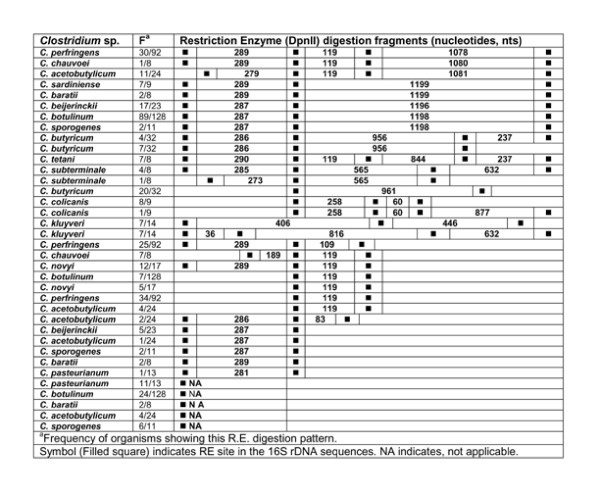
***In silico *Restriction Enzyme activity in 16S rDNA sequences of *Clostridium *spp.: DpnII**. ^a^Frequency of organisms showing this RE digestion pattern. Symbol (Filled square) indicates RE site in the *rrs *sequences. NA indicates, not applicable.

**Figure 17:**
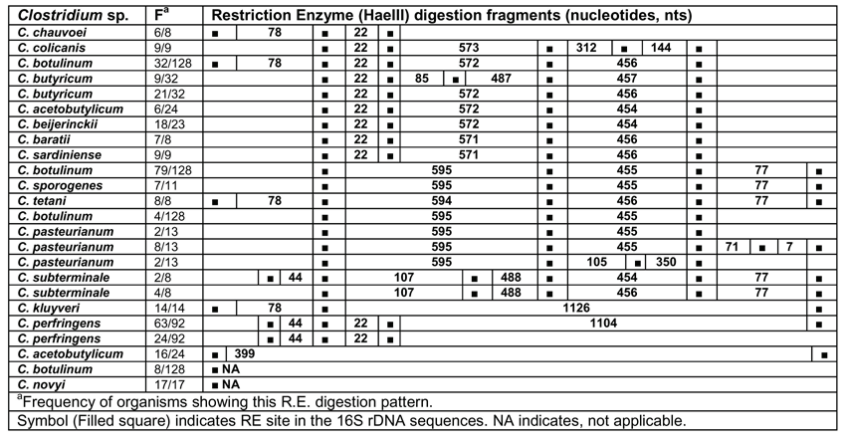
***In silico *Restriction Enzyme activity in 16S rDNA sequences of *Clostridium *spp.: HaeIII**. ^a^Frequency of organisms showing this RE digestion pattern. Symbol (Filled square) indicates RE site in the *rrs *sequences. NA indicates, not applicable.

**Figure 18:**
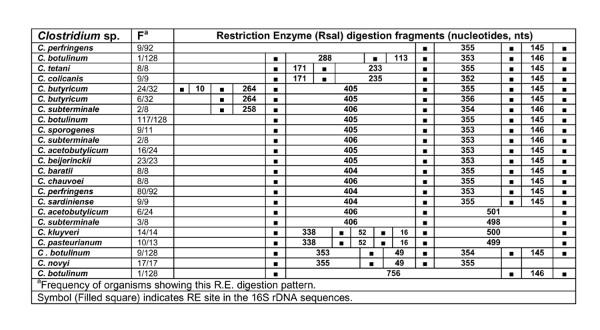
***In silico *Restriction Enzyme activity in 16S rDNA sequences of *Clostridium *spp.: RsaI**. ^a^Frequency of organisms showing this RE digestion pattern. Symbol (Filled square) indicates RE site in the *rrs *sequences.

**Figure 19:**
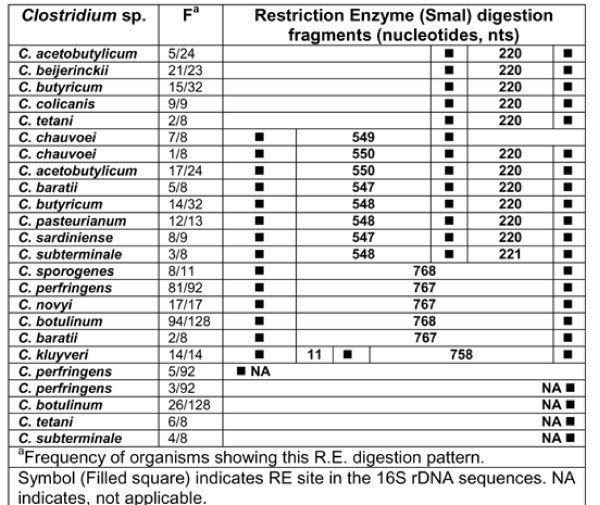
***In silico *Restriction Enzyme activity in 16S rDNA sequences of *Clostridium *spp.: SmaI**. ^a^Frequency of organisms showing this RE digestion pattern. Symbol (Filled square) indicates RE site in the *rrs *sequences. NA indicates, not applicable.

**Figure 20:**
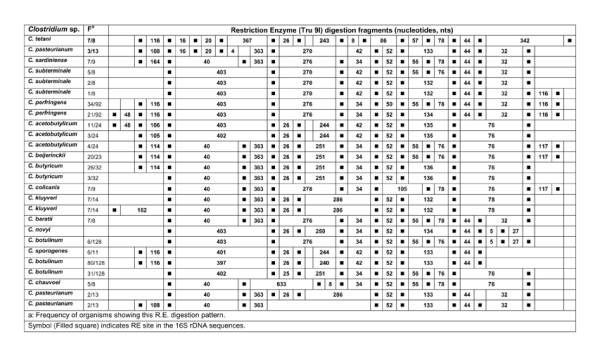
***In silico *Restriction Enzyme activity in 16S rDNA sequences of *Clostridium *spp.: Tru9I**. ^a^Frequency of organisms showing this RE digestion pattern. Symbol (Filled square) indicates RE site in the *rrs *sequences.

Our recent studies [3,49] have revealed a high level of latent characteristics within *rrs *sequences based on *in silico *digestion with 14 Type II REs. These *in silico *digestions have resulted in predicting clear cut fragment lengths in the *rrs *with six REs: AluI, BfaI, DpnII, HaeIII, RsaI and Tru9I [3,49]. In the present study, 7 REs - AluI, BfaI, DpnII, HaeIII, RsaI, SmaI and Tru9I were found effective in drawing meaningful conclusions (Figures 14, 15, 16, 17, 18, 19 and 20). RE sites for BamHI, NotI, PstI and SacI were almost completely absent from the *rrs *sequences of all the strains of 15 *Clostridium *spp. and thus proved to be ''non"-cutters (Additional file 7: Table S4). Another group, which did not permit clear cut conclusions to be drawn was composed of the following three REs: EcoRI, HindIII, and NruI, with one RE site each (leading to two fragments of varied lengths) in most of the *Clostridium *strains.

### AluI

*In silico *digestion of *rrs *sequences of 15 different *Clostridium *spp. with RE - AluI resulted in 4-8 fragments. The segregation of *Clostridium *species was evident at two levels - (i) intra-species and (ii) inter-species. Intra-species variation was observed among *C. pasteurianum *- *rrs *sequences, which were found to possess 4-7 RE - AluI digestion sites. Two fragments of 186-610 nts were found in all the four groups. At the 5' end, upstream of the 186 nts fragments, the RE sites were not detectable such that the two fragments of 12 and 49 nts seem to have merged together to generate a 61/62 nts long fragment. Similarly on the 3' end, the merger of the two fragments of 207 and 142 nts resulted in a larger nucleotide fragment of 349 nts (Figure 14).

Intra-species level segregation was also evident in case of *C. botulinum*. Here, the 120/128 *rrs *sequences were found to be distributed into 5 groups of 5, 10, 21, 22 and 62 sequences each. The largest group of 62 sequences of *C. botulinum *had 5 RE - AluI digestion sites resulting in the following fragments between them: 170-795-265-85 nts. The next group of 21 sequences of *C. botulinum *was quite close to the former. However, it lacked a RE site at the 5' end, such that one could not predict the presence of 170 nts sized fragment. The two smaller groups of 10 and 22 *rrs *sequences of *C. botulinum *had 6-7 RE - AluI sites each. These two groups shared the following fragment sizes and the order of their occurrence: 566-228/230-205/207-57-109 nts. The later group of 22 of *C. botulinum *sequences had an additional RE site which led to the prediction of a 170 nts fragment at the 5' end of this gene. It may be remarked that these four groups differed because of the absence/presence of RE sites (Figure 14).

Variability in the RE - AluI sites among *rrs *sequences of *C. acetobutylicum *was also quite high. Out of 24 *rrs *sequences, 17 of them showed two RE - AluI sites resulting in clear cut fragment of 797 nts. On the other hand, a small group of 3 sequences had additional RE sites, which resulted in additional fragments of 170 nts at 5' end and 266 nts at 3' end. *C. butyricum *3/32 and 3/24 *C. acetobutylicum rrs *sequences show high homology in their RE - AluI digestion pattern. *C. baratii *and *C. sardiniense *had similar RE patterns leading to the following order of fragments - 186-604/605-206-57-109 nts. The situation of *C. colicanis *was unique as it resembled *C. baratii *and *C. sardiniense *except in the absence of a RE - AluI site resulting in the merger of 206-57 nts fragments into a single fragment of 264 nts. On the other hand, *C. colicanis *showed high resemblance in its *in silico *RE digestion pattern to most *C. perfringens *(66/92 sequences) (Figure 14).

A perusal of all the numbers and the order of occurrence of the fragments predicted to be generated by RE -AluI *in silico *digestion enabled us to segregate the following: (i) *C. acetobutylicum *(17/24 sequences), (ii) *C. chauvoei*, (iii) *C. colicanis*, (iv) *C. kluyveri*, (v) *C. pasteurianum*, (vi) *C. perfringens*, and (vii) *C. subterminale *(Additional file 4: Table S2). *C. sporogenes*/*C. botulinum *(62/128 sequences) are indistinguishable here but can be distinguished on the basis of other REs (Figure 14).

### BfaI

With RE - BfaI *in silico *digestion of *rrs *sequences of 15 *Clostridium *species predicted the presence of 5-8 fragments of distinct nucleotide sizes. Intra-species level variability among RE - BfaI generated fragments was evident in *C. acetobutylicum *and *C. botulinum*. RE digestion of *C. acetobutylicum rrs *sequences resulted in segregating them into two populations - (i) 16/24 sequences and (ii) 4/24 sequences. These two populations of *C. acetobutylicum *showed high similarity in RE pattern towards their 3' end: 146-159/160-161/164-68-126 nts fragments. They differed by the presence of two additional RE - BfaI sites, which resulted in two more fragments of 378-29 nts towards the 5'end of the 4/24 *rrs *sequences of *C. acetobutylicum *(Figure 15).

On the basis of RE - BfaI *in silico *digestion of *rrs *of *C. botulinum*, high genetic heterogeneity was evident among the 128 isolates. The four groups of *C. botulinum *were composed of as follows: (i) 57, (ii) 27, (iii) 9, and (iv) 34/128 sequences. All the four groups of *C. botulinum *could be easily distinguished on the basis of their RE - BfaI digestion fragment lengths. The first group of 57/128 *C. botulinum *had a unique RE - BfaI digestion pattern: 195-20-163-29-146-185-331 nts in their *rrs*. Second group of 27/128 *C. botulinum *had similarity only with *C. sporogenes *for their RE - BfaI digestion pattern: 378-29-146-185-331 nts in their *rrs*. It differed from the first group due to merger of three fragments 195-20-163 at the 5' end in to a single fragment of 378 nts in the latter. The third group of 9/128 *C. botulinum *were found to possess an *in silico *RE - BfaI digestion pattern: 378-29-146-158-231-126 nts in their *rrs *sequences. It was similar to that observed in *C. novyi *and *C. tetani*. The fourth cluster of *C. botulinum *(34/128 sequences), did not show any genetic variability with respect to RE - BfaI *in silico *activities with respect to those observed in *C. beijerinckii*, *C. butyricum*, *C. sardiniense*, *C. subterminale*, and *C. acetobutylicum *(4/24 sequences) (Figure 15). This feature implies that these species might have a common phylogenetic origin.

On the basis of inter-species variation in the RE - BfaI sites on the *rrs *the following *Clostridium *species can be distinguished from the rest of the species: *C. acetobutylicum *(16/24 sequences), *C. baratii, C. botulinum *(57/128 sequences), *C. chauvoei*, *C. colicanis*, *C. kluyveri*, *C. pasteurianum *and *C. perfringens *(Additional file 4: Table S2).

### DpnII

In the case of *in silico *digestion of *rrs *sequences with RE - DpnII, a large intra-species variation was evident in the cases of 9 different *Clostridium *species: (i) *C. acetobutylicum*, (ii) *C. baratii*, (iii) *C. beijerinckii*, (iv) *C. botulinum*, (v) *C. butyricum*, (vi) *C. kluyveri*, (vii) *C. novyi*, (viii) *C. pasteurianum *and (ix) *C. perfringens*. On the other hand, interspecies variation was evident in the cases of *C. chauvoei, C. colicanis*, *C. sardiniense*, *C. subterminale *and *C. tetani*. In spite of high homology among different *Clostridium *species in terms of the order, number and length of the fragments generated by RE - DpnII, the following seven *Clostridium *species: *C. butyricum*, *C. chauvoei*, *C. colicanis*, *C. kluyveri*, *C. subterminale *and *C. tetani *could still be easily distinguished (Figure 16, Additional file 4: Table S2). On the other hand, in spite of certain unique features in *C. acetobutylicum *and *C. perfringens*, intra-species variation did not allow complete segregation and may need supplementary information from other REs.

### HaeIII, RsaI, SmaI and Tru9I

*In silico *RE - HaeIII, RsaI, SmaI and Tru9I digestion of *rrs *of 15 *Clostridium *species resulted in predicting the presence of 1-12 sites. Due to high variation in the characteristics of the nucleotide fragments within and among *Clostridium *species, a unique RE digestion pattern was observed in in the following cases: (i) *C. chauvoei, C. colicanis, C. kluyveri *and *C. tetani *with HaeIII (Figure 17, Table 5), (ii) *C. novyi *with RsaI (Figure 18, Table 5), (iii) *C. colicanis *with SmaI (Figure 19, Table 5) and (iv) *C. baratii*, *C. novyi *and *C. sardiniense *with Tru9I (Figure 20, Table 5). It implies limited scope for exploiting these REs for identification purposes.

**Table 5:** Restriction enzymes with unique *in silico *digestion pattern of 16S rDNA of different *Clostridium *species

*Clostridium *species	Restriction Enzyme (RE)						
	
	AluI	BfaI	DpnII	HaeIII	SmaI	Tru9I	RsaI
*C. baratii*	-^a^	U^b^	-	-	-	U	-

*C. butyricum*	-	-	U	-	-	-	-

*C. chauvoei*	U	U	U	U	-	-	-

*C. colicanis*	-	U	U	U	U	-	-

*C. kluyveri*	U	U	U	U	U	-	-

*C. novyi*	-	-	-	-	-	U	U

*C. pasteurianum*	U	U	-	-	-	-	-

*C. perfringens*	-	U	-	-	-	-	-

*C. sardiniense*	-	-	-	-	-	U	

*C. subterminale*	U	-	U	-	-	-	-

*C. tetani*	-	-	U	U	-	-	-

*C. acetobutylicum *(CAcI)^c^	U	U	-	-	-	-	-

*C. botulinum *(CBoI)^c^	-	U	-	-	-	-	-

^a^No unique digestion pattern was detected

^b^It denotes unique in terms of fragment length (nucleotides), order and number

^c^In the cases of *C. acetobutylicum, C. beijerinckii*, *C. botulinum *and *C. sporogenes*, no unique pattern could be detected and have been discussed separately. The different groups are based on their phylogenetic relationship (Figure 1). It is further assumed here that *C. beijerinckii *is a part of population comprised of *C. acetobutylicum *and similarly *C. sporogenes *is a part of *C. botulinum*.

*In silico *RE digestion of *rrs *of 15 *Clostridium *species enabled us to identify unique features (order of occurrence and length, nts) of this gene which can be used as bio-markers (Table 5). *C. butyricum*, *C. perfringens *and *C. sardiniense *could be distinguished from all other *Clostridium *spp. on the basis of the *in silico *digestion with RE - DpnII, BfaI and Tru9I, respectively. *C. baratii*, *C. novyi*, *C. pasteurianum*, *C. subterminale *and *C. tetani *could be distinguished from the rest of the species on the basis of *in silico *digestion pattern of their *rrs *with all REs except SmaI. The most interesting features seem to reside in the *rrs *sequences of *C. chauvoei*, *C. colicanis *and *C. kluyveri*, which possessed unique pattern of active sites for 4-5 different REs such that any one of these was sufficient to identify them. In the cases of *C. acetobutylicum, C. beijerinckii*, *C. botulinum *and *C. sporogenes*, no unique pattern could be detected. For the groups comprised of (i) *C. botulinum *- *C. sporogenes *and (ii) *C. acetobutylicum - C. beijerinckii*, we could not detect any unique RE digestion patterns and may need to be compared with a combination of REs. The close phylogenetic relationship between *C. botulinum *and *C. sporogenes *has been reported previously also [51]. Since they differ on the basis of their phylogenetic relationship (Figures 1, 4 and 5), it appears that *C. beijerinckii *is a part of population comprised of *C. acetobutylicum *and similarly *C. sporogenes *is a part of isolates belonging to *C. botulinum*. Alternatively, the issue can be resolved by creating new species by subdividing *C. acetobutylicum *and *C. botulinum*.

### Validation of framework sequences by *in silico *RE activity on 16S rDNA sequences of organisms identified as *Clostridium *sp

The second level of investigation after segregating *Clostridium *sp. (95 isolates) (Table 3) on the basis of phylogenetic frame work was to validate them on the basis of unique RE digestion patterns deduced from the *rrs *sequences of 15 known *Clostridium *spp.

#### C. acetobutylicum

In the case of *C. acetobutylicum*, two distinct populations were observed in the initial phylogenetic tree which was based on *rrs *sequences of 24 isolates of this species. A similar trend of two major groups was very clearly evident also on the basis of RE - AluI *in silico *digestion patterns: (i) 186-611-264 nts and (ii) 798 nts. The 29 *Clostridium *sp., which we could classify as *C. acetobutylicum *on the basis of phylogenetic framework (Table 3) were further supported by RE digestion patterns of their *rrs *sequences. Here also we could observe that the total population was composed of 2 different groups, consisting of 12 and 17 organisms each. The two groups segregated due to single nucleotide polymorphism (SNPs) at RE sites. Modifications in RE sites can arise primarily through single nucleotide insertion, deletion and/or substitution [52].

#### *C. beijerinckii*

High homology between *C. beijerinckii *and *C. acetobutylicum *was evident due to similarities in *in silico *digestion pattern generated by the action of most of the REs employed in this study especially - AluI, BfaI, HaeIII, RsaI, SmaI and Tru9I. However, it was "unique" in its RE pattern with respect to DpnII. The *rrs *sequences of *C. beijerinckii *on digestion with RE - Tru9I showed a pattern of 114-40-363-26-251-34-52-56-76-76-117 nts, which is quite similar to that recorded with a proportion of *C. acetobutylicum *population. However, these could be distinguished on the basis of RE - DpnII *in silico *digestion fragments 287 or 287-1196 nts found in *rrs *sequences of *C. acetobutylicum*, but were absent in these 13 newly identified *C. beijerinckii *isolates. Thus, we recommend the usage of a combination of DpnII and Tru9I to validate the identification of 13 *Clostridium *sp. as *C. beijerinckii *(Table 3).

#### *C. subterminale*

The phylogenetic framework based on 8 *rrs *sequences of *C. subterminale *allowed identification of 2 unique *in silico *RE digestion patterns with AluI and DpnII. The validation of 20 *Clostridium *sp. which could be classified as *C. subterminale *through phylogenetic framework (Table 3) was done through *in silico *RE digestion of their 16S rDNA with both the REs. Thus *in silico *digestions supported the segregation of these *Clostridium *sp. as *C. subterminale*. Two of three populations were distinct on the basis of the uniqueness of their fragment characteristics - number, length and order of occurrence (i) RE - AluI a) 345-450-347 nts and b) 170-796-207-143 nts and (ii) RE - DpnII a) 285-565-632 and b) 273-565 nts. However, the third group of isolates identified as *C. subterminale *was intermediate to the other two populations with respect to the patterns obtained with both the REs - AluI and DpnII.

#### *C. botulinum *and *C. sporogenes*

*C. botulinum *sp. produces seven serotypes (A through G) of botulinum neurotoxins (BoNTs) [16,20]. It shows 4 distinct lineages (I-IV) [33,50], where good correlations have been found between their phenotypes and phylogenetic classification: (i) Group I - proteolytic *C. botulinum *types A, B and F which are close to *C. sporogenes*, (ii) Group II consists of saccharolytic types B, E and F, (iii) Group III includes types C and D and *C. novyi *type A and (iv) Group IV has *Clostridium argentinense *(*C. botulinum *type G) and *C. subterminale *[32]. Based on DNA S1 nuclease analysis, *C. botulinum *was found to be genetically close to *C. sporogenes *at the species level, however, the later was proposed to be conserved for non-toxigenic strains [51].

In a phylogenetic tree based only on the framework sequences, *C. botulinum *and *C. sporogenes *were found to occur together on a single clade. A comparison of the different *in silico *RE digestion patterns of *rrs *of *C. sporogenes *and *C. botulinum *showed exact homology in the cases of REs - AluI, BfaI, HaeIII, RsaI, Tru9I and SmaI except DpnII (Figures 14, 15, 16, 17, 18, 19 and 20). In the case of DpnII, *C. sporogenes *was segregated into 2 populations, where one of them showed exact homology with *C. botulinum *and the other one was different and unique in comparison to all other *Clostridium *species. It may not be too inappropriate to suggest that *C. sporogenes *is perhaps a sub-species of *C. botulinum *or it may find its appropriate place if *C. botulinum *can be reclassified as 4 different sub-species. This proposal finds support from the RE pattern of sequences of *Clostridium *species (S000357670 and S000357671) which had been classified as *C. sporogenes *with the help of phylogenetic frame work. Nine *Clostridium *sp. which appeared close to *C. botulinum*, *C. sporogenes *and *C. novyi *(Table 3) were found to be similar to each other with respect to the *in silico *activities of one or more REs - Tru9I, HaeIII, AluI and BfaI.

#### Other *Clostridium *spp

The rest 24 *Clostridium *sp. identified up to species level on the basis of phylogenetic framework (Table 3), could be supported also by high similarity in the *in silico *RE digestion pattern of their *rrs*: (i) *C. tetani *- HaeIII, (ii) *C. chauvoei *- AluI and BfaI, (iii) *C. kluyveri *- AluI, (iv) *C. pasteurianum *and *C. perfringens *- BfaI and (v) *C. baratii *- Tru9I and BfaI (Figures 14, 15, 17 and 20).

The phylogenetic relationships observed among *Clostridium *spp. reported to occur with low frequency formed quite distinct clusters. Sixty eight *rrs *sequences belonging to 37 species were found to clusters along with 14 *Clostridium *spp. used for framework analysis. The close relationship among the different *Clostridium *spp. was validated by comparing their *in silico *analysis RE digestion pattern of their *rrs *(Table 4), which matched invariably with the unique RE pattern of known *Clostridium *spp. It is perhaps the first report where in 37 different *Clostridium *species have been segregated among 14 groups based on phylogeny and *in silico *RE digestion pattern of their *rrs*.

### Nucleotide signature analysis - frequency and distribution pattern

To further validate the segregation of *Clostridium *sp. done on the basis of phylogenetic frame work, we investigated them for the presence of nucleotide signatures (30 nts) deduced from isolates of the 15 known *Clostridium *spp. The validity of the nucleotide signatures was done through the frequency of occurrence of the reference organism among the top 10 hits (BLAST).

The sequences of 8-128 data sets submitted group-wise to MEME (Multiple EM for Motif Elicitation) program http://meme.sdsc.edu/meme4_5_0/cgi-bin/meme.cgi revealed ten signatures (30 nts) for each species. Regular expression diagram of signatures (nts) (Additional file 8: Figures S20,S21,S22,S23,S24,S25,S26,S27,S28,S29, S30,S31,S32,S33,S34,S35 and S36) and the frequency of occurrence of motifs obtained through MEME for different taxonomic groups have been presented with reference to 15 *Clostridium *spp. (Additional file 9: Tables S5-S20).

Out of the 10 signatures (30 nts), identified in each of the 15 different *Clostridium *spp., unique signatures in the *rrs *sequences (Table 6) were found in the following cases: *C. beijerinckii *and *C. sporogenes *- 1 each, *C. butyricum *- 2, *C. colicanis *- 4, *C. kluyveri, C. pasteurianum *and *C. perfringens *- 5 each, *C. chauvoei *- 6 and *C. tetani *- 9. However, using similar strategy, no unique signatures could be identified in the cases of (i) *C. baratii *and *C. sardiniense*, (ii) *C. botulinum, C. novyi *and *C. subterminale *and (iii) *C. acetobutyilicum*. These latter 6 *Clostridium *spp. have been dealt with separately as detailed below.

**Table 6:** Characteristics of "unique" nucleotide signatures for *rrs *sequences of 15 different *Clostridium *spp

*Clostridium *sp.	Signature		
	
	Nucleotide sequence (30 nts)	No.^a^	Frequency^b^
*C. beijerinckii*	ATATCTAGAGTGCAGGAGAGCAAAGTAGAA	M10	23/23

*C. sporogenes*	AATCGCATGATTATCTTATCAAAGATTTAT	M1	11/11

*C. butyricum*	ACTTGACATCTCCTGAATTACTCTGTAATG	M3	32/32
	TACAATGGTCGGTACAATGAGATGCAACCT	M9	32/32

*C. colicanis*	AAAGGGAGATTAATACCTCATAATATCCTA	M1	9/9
	AAAACTTTAAAACCGGTCTCAGTTCGGATT	M3	9/9
	ATACATGGATTAAAGGAGCAATCCGCTATA	M5	9/9
	TAGGCGGATCTTTAAGTGGGATGTGAAATA	M9	9/9

*C. kluyveri*	AGATTAATACCGCATAGAAGGTAAAAATCG	M2	14/14
	AGGCGGATATTTAAGTGAGATGTGAAAGAC	M4	14/14
	AGTGCATTTCAAACTGGATATCTAGAGTGC	M5	14/14
	AAATCTCAAAAACTGCCCCCAGTTCGGATT	M9	14/14
	AAGAAGGTTTTCGGATCGTAAAGCTCTGTC	M10	14/14

*C. pasteurianum*	CGAAAGGGAGATTAATACCGCATAATATTA	M2	13/13
	AAATGCGTAGAGATTAGGAAGAACATCAGT	M3	13/13
	ATTGTAAAGCTCTGTCTTTTGGGACGATAA	M5	13/13
	TAAACGATGAGTACTAGGTGTAGGAGGTAT	M7	13/13
	TACAATGGTGAGAACAACGAGATGCAATAC	M8	13/13

*C. perfringens*	TTAGTTACTACCATTAAGTTGAGGACTCTA	M1	92/92
	ACACTTGACATCCCTTGCATTACTCTTAAT	M2	92/92
	TAGGCGGATGATTAAGTGGGATGTGAAATA	M3	92/92
	AAGGTTTTCGGATCGTAAAGCTCTGTCTTT	M4	92/92
	TTATGTGTAGGGCTACACACGTGCTACAAT	M5	92/92

*C. chauvoei*	AAAGGAAGATTAATACCGCATAATATTGCA	M1	8/8
	AGACTTGACATCTCCTGCATTACTCTTAAT	M3	8/8
	AGTAATTAAAGGAGCAATCCGCTACAAGAT	M4	8/8
	TAAACTATAATACTTGTCTCAGTTCGGATT	M5	8/8
	AACTTGGGTGCTGCATTTCAAACTGGAAGT	M8	8/8
	AAGGTTTTCGGATCGTAAAGCTCTGTCTTC	M9	8/8

*C. tetani*	AACCCTTATTATTAGTTGCTACCATTAAGT	M1	8/8
	TTAAGTGAGATGTGAAATACCTAAGCTTAA	M2	8/8
	AAAGGAGGATTAATACCGCATAAAGTTAAG	M3	8/8
	TTTAACCAAAGGAGTAATCTGCTTTGAGAT	M4	8/8
	AGTTGCTAGTAATCGCAAATCAGAATGTTG	M5	8/8
	TTCTGTGCCGCAGTTAACACATTAAGTATT	M6	8/8
	AAATCTCAAAAACCGATCCCAGTTCGGATT	M7	8/8
	AAGGTTTTCGGATCGTAAAACCCTGTTTTC	M8	8/8
	ACGGTCGCAAGACTAAAACTCAAAGGAATT	M9	8/8

*C. baratii*^c^	TTGTAAAGCTCTGTCTTTGGGGACGATAAT	M3	8/8
	ATTTTTAAGTGGGATGTGAAATACCCGGGC	M6	8/8

*C. sardiniense*^c^	AAAGGAAGATTAATACCGCATAACATTGCA	M1	9/9
	TAAACTTCAAAACTTGTCTCAGTTCGGATT	M2	9/9
	TTCGCATGAAACAGCAATTAAAGGAGCAAT	M4	9/9
	CTACAATGGCAAGTACAGAGAGATGCAATA	M9	9/9
	GTAAACGATGAATACTAGGTGTAGGGGTTT	M10	9/9

*C. botulinum* (CBoI - 83/128) ^c,d^	AAAACTTATAAAACCTATCTCAGTTCGGAT	M1	82/83
	AACCCTTGTTATTAGTTGCTACCATTAAGT	M2	83/83
	AAGGTCTTCGGATTGTAAAGCCCTGTTTTC	M7	83/83
	GTAGGCGGATGTTTAAGTGGGATGTGAAAT TAAACGATGGATACTAGGTGTAGGGGGTAT	M8 M9	83/83 82/83

*C. novyi*^c^	AAAGGGAGATTAATACCGCATAACATTATT	M1	17/17
	AAGATTAAAACTCAAAGGAATTGACGGGGA	M3	17/17
	ACTTTCTGGACTGTAACTGACACTGAGATA	M6	17/17
	TTAAGTCAGATGTGAAATTCCCGGGCTTAA	M10	17/17

*C. subterminale*^c^	AATGAAGAAGGCCTTAGGGTTGTAAAGTTC	M6	8/8

*C. acetobutylicum*	No unique signature could be found	-	24/24

^a^Refer to Additional file 8: Figures S20-S36 and Additional file 9: Tables S5-S20

^b^Frequency of occurrence of the signature out of the total sequences screened.

^c^Can be designated as unique signatures if we assume i) *C. baratii *and * C. sardiniense *and ii) *C. botulinum, C. novyi *and *C. subterminale *to belong to single sp. based on phylogenetic framework and *in silico *analyses

^d^Out of the two groups of 45 and 83 organisms, no unique signature could be detected in the first group

#### *C. baratii *and *C. sardiniense*

Out of 10 signatures deduced among the *rrs *sequences of 8 isolates of *C. baratii *only two - M3 and M6 could be designated as "unique". In fact, these two signatures were absent from all other *Clostridium *spp. except *C. sardiniense*. Similarly, in *C. sardiniense *6 out of 10 nucleotide signatures were found to be "unique" to it but were present in the overall *rrs *sequence of *C. baratii*. We may conclude that although these two *Clostridium *spp. did not show any exact resemblance in their signatures, however, they shared 19 out of 20 signatures in their overall *rrs *sequence. Thus it is difficult to use them for segregating the two *Clostridium *species. This observation of high homology is further supported by the fact that *C. baratii *and *C. sardiniense *are phylogenetically present on a single clade and had high BV in the range of 654-999 (Figure 1). In spite of such high similarity they could be separated on the basis of nucleotide signature (M2) of *C. baratii *- 5-TATTGTTAGTTGCTACCATTTAGTTGAGCA-3' (which incidentally was present in most other *Clostridium *species).

#### *C. botulinum *and *C. acetobutylicum*

Organisms belonging to *C. botulinum *were segregated phylogenetically into 4 groups. Distinct nucleotide signatures could be detected on segregating 128 *rrs *sequences of *C. botulinum *isolates into two groups - i) 83 equivalent to CBoI of the phylogenetic tree (Additional file 2: Figure S1) and ii) 45 representing (CBoII-CBoIV) (Additional file 2: Figure S1). The first group of 83 isolates had 5 unique nucleotide signatures, which could be used to distinguish them from all other *Clostridium *species except *C. sporogenes *isolates. On the other hand, 45 *rrs *sequences of isolates belonging to CBoII-CBoIV could not be distinguished on the basis of the 10 nucleotide signatures deduced from their *rrs *sequences. Previous reports of screening with four REs and 30 primers combinations revealed that HindIII and HpyCH4IV are effective in demonstrating extensive diversity among *C. botulinum *type E, whereas *C. botulinum *Group I indicted low genetic diversity [50,53,54]. A similar scenario was evident also in the case of *C. acetobutylicum*. It prompted us to conclude that *C. acetobutylicum *and *C. botulinum *perhaps represent ancestral *Clostridium *species.

### Validation of framework sequences for *Clostridium *sp: nucleotide signatures

To validate the categorization and classification of 95 *rrs *sequences of *Clostridium *sp. belonging to 13 *Clostridium *spp. (Additional file 9: Table S21), we looked up for 10 nucleotide signatures of the reference framework sequences in them. Of these 10 signatures, the emphasis has been laid on i) the signatures unique to a particular *Clostridium *sp. and ii) presence of rest of the signatures. On the basis of these two criteria, we may conclude that *Clostridium *spp.: *C. subterminale *- 16/20 sequences, *C. tetani *- 2/7 sequences, *C. perfringens *- 1/1 sequence, *C. pasteurianum *- 4/4 sequences, *C. baratii *and *C. sardiniense *- 5/5 sequences showed quite close match with the reference framework sequences. On the other hand, *C. beijerinckii *had only one unique signature which was absent in 13/13 sequences of *Clostridium *sp. but due to the presence of all other signatures we may conclude that they are quite close to the reference framework sequences. Similarly, *C. acetobutylicum *showed that 29/29 *Clostridium *sp. were quite similar to the reference sequences. This implies that in these cases signature analysis supports the framework sequence based analysis. However, in the following *Clostridium *spp., the identified *Clostridium *sp. did not show much homology with respect to the signatures found in the reference framework sequences: (i) *C. botulinum*, (ii) *C. chauvoei, C. kluyveri*, *C. novyi *and *C. sporogenes*.

## Discussion

Molecular techniques have added new dimensions and sensitivity to the phylogeny and taxonomy of organisms. The most remarkable and widely acknowledged are the unique features of highly conserved gene - *rrs *[55]. Based on *rrs *sequencing Collins et al. revealed the polyphyletic nature of *Clostridium sensu stricto *[35]. Since then there have been many additions and rectifications in the classification of *Clostridium *[56,57]. There are two major issues with respect to Clostridial taxonomy and phylogeny. First is the high genetic diversity - GC content, which varies from 24 mol% (*C. perfringens*) [47] to 58 mol% (*C. barkeri*) [48]. It is perhaps too wide a range to encompass a single genus [42] and may need revision. Secondly, *Clostridium *comprises a very heterogeneous group of bacteria representing more than 110 spp., with a large proportion having only 1-5 strains. The question is whether we should split the genus *Clostridium *(especially *C. acetobutylicum *and *C. botulinum*) and/or we may merge quite a few of its species (small sized). In certain cases of recent speciation events, where *rrs *is not sufficient to discriminate phylogenetically close relatives, one resorts to DNA-DNA reassociation technique [58]. In spite of an ever increasing knowledge about various conserved genes for distinguishing microbes [3], *rrs *continues to be quite effective in segregating and even in reclassifying quite a few microbial isolates as new genera and species. A large number of organisms reported initially as *Bacillus *species [59] have been reclassified as genus *Geobacillus *[60], *Sporosarcina *[61], or *Marinibacillus marinus *[62]. In fact, many species of *Clostridium *[35] have also been reclassified as new genera, such as *Sedimentibacter *[63]. The three *Clostridium *spp. - *C. carboxidivorans*, *C. drakei*, and *C. scatologenes*, which showed high (99.7-99.8%) sequence similarity for their *rrs *sequences [64] were later found to be distinct species based on their DNA-DNA reassociation values [58]. In spite of such high volume of data available on *rrs *http://rdp.cme.msu.edu/, the fact remains that it needs support from other genes to arrive at authentic conclusions. Does it imply that *rrs *is already at its dead end? or Do we need to look deeper into the latent and as yet un-explored features of this wonderful gene?

Our recent studies on the diversity of *Bacillus *spp. [3] and *Stenotrophomonas *isolates [49] revealed certain very interesting 'latent' features of *rrs*. We could designate *rrs *of certain isolates as phylogenetic framework sequences. These were supported by species specific unique signatures and *in silico *RE patterns. Together, all these features of *rrs *allowed us to (i) classify *Bacillus *sp., reveal novel lineages and distinguish the two subgroups of *B. subtilis *[3] and (ii) evaluate the biodiversity of *Stenotrophomonas *isolates [49]. These studies indicate towards unexplored features within the *rrs *to define and circumscribe the genus *Clostridium sensu stricto *[52].

The species-specific phylogenetic framework developed here proved instrumental as a tool to deducing the limits of genetic diversity within 15 *Clostridium *spp. The validity of the phylogenetic framework sequences was established by clear cut segregation of members of a given *Clostridium *sp. against a total of 56 *rrs *sequences (Table 2, Figure 1). High genetic heterogeneity was recorded among members of *C. acetobutylicum *and *C. botulinum *in our study. It has been previously supported by significant differences in their physiological and genetic characteristics [65,66]. Within the 56 framework sequences of 16S rDNA of 15 *Clostridium *spp. based phylogenetic tree, we observed the 5 framework sequences of *C. acetobutylicum *to segregate in to two groups: (i) 2 sequences were close to *C. beijerinckii *and *C. butyricum *and (ii) 3 were close to *C. pasteurianum *(Figure 1). The findings in our study find support from previous phylogenetic studies based on concatenated sequences of 37 proteins, which showed that *C. acetobutylicum *clusters along with *C. beijerinckii *in addition to other species such as *C. botulinum, C. kluyveri, C. novyi*, *C. perfringens*, and *C. tetani *with 100% BV [52]. In fact, 24 strains belonging to *C. acetobutylicum *were reclassified as either *C. beijerinckii *(Sub group 3) or *C. saccharobutylicum *[25,46]. Here, we observed that some strains of *C. acetobutylicum *are phylogenetically close to *C. felsineum*, however DNA-DNA hybridization study [34] shows them to be distinct species.

Similarly, *C. botulinum *is well known to for its segregation on the basis of a wide range of neurotoxins produced by them [16,28,67]. Here in our study, *C. botulinum *16S rDNA sequences showed 4 clear grouping in accordance with those reported in literature [32]: (i) *C. sporogenes*, (ii) a group of *C. butyricum *and *C. acetobutylicum*, (iii) *C. novyi *and (iv) *C. subterminale*. This close relationship among the species of *Clostridium *cluster I (*C. botulinum, C. acetobutylicum *and *C. novyi*) finds support from the presence of 4 amino acid insert in a highly conserved region of Gyrase A protein, which could not be traced in any other Clostridia or other group of bacteria [52]. High phylogenetic closeness based on *rrs *has been reported between *C. botulinum *and *C. sporogenes *[18,68] and *C. botulinum *type G and *C. subterminale *[32]. Three distinct lineages of *C. botulinum *were confirmed by AFLP analysis of their *rrs *sequences [32]. In the present work, other close relationships were observed among (i) *C. baratii*, *C. colicanis *and *C. sardiniense *(BV 840-1000) and (ii) *C. botulinum *and *C. novyi *(BV 703-1000). A complete molecular typing of *C. botulinum *is needed for correct classification of this species [50]. In depth analysis of *rrs *reported here can in fact provide enough supplementary information to counter multilocus sequence typing recommended for elucidating phylogeny of *C. botulinum *[28]. On the basis of the evidences provided here (Additional file 2: Figure S1, Figures 1, 14, 15, 17, 18, 19 and 20), we further propose here that *C. botulinum *needs to be reclassified in to 4 different (sub)-species, as is generally believed by many researchers [16]. The validity of Framework sequences was further confirmed in the case of the classification of 95 *Clostridium *sp. up to species level, by *in silico *RE digestions especially - AluI, BfaI and DpnII (Figures 14, 15, 16, 17, 18, 19 and 20).

Our present analysis which shows a close phylogenetic relationship between *C. subterminale *and certain *Clostridium *spp. such as *C. argentinense, C. estertheticum*, and *C. thiosulfatireducens *(Table 4), finds support from the previous reports of Spring et al., [69]. Although *C. tetanomorphum *has been observed to be close to *C. subterminale *and *C. thiosulfatireducens *with a 97% BV [70] but we found it to be close to *C. tetani *(BV 982). Unlike previous studies [17,42,71], we could demonstrate closer relationships among (i) *C. puniceum, C. beijerinckii *and *C. butyricum *and (ii) *C. haemolyticum *and *C. novyi *with a much smaller number of *rrs *frame work sequences.

Quite a few species of other Clostridial genera in Cluster I belong to *Eubacterium moniliforme, E. terantellae, Ancerobacter polyendosporus, Sarcina ventriculi *and *S. maxima *[42]. As far as *Eubacterium *is concerned, several *Clostridium *spp. have been reclassified within newly created genera: *Moorella *and *Oxobacter *[45] or within *Eubacterium *itself [35]. *C. barkeri*, a member of Cluster XV was found close to *Eubacterium limosum *with a level of similarity as high as 95% [35]. In Cluster XVI: phylogenetically significant association was observed among the 3 species - *Eubacterium biforme*, *C. innocuum*, and *Streptococcus pleomorphus *[35]. In the present study, *Eubacterium combesii, E. moniliforme *and *E. budayi *were found to cluster with *C. botulinum, C. baratii *and *C. sardiniense*, respectively.

High similarity in the nucleotide signatures within *rrs *sequences of *C. acetobutylicum *and *C. botulinum *prompt us to speculate that *C. acetobutylicum *and *C. botulinum *might have common ancestor. In fact, these two *Clostridium *spp. shared 34-35% of the total *rrs *sequences. Similarly, we have observed that certain signatures are common to almost all the 15 *Clostridium *spp. which were used for developing framework sequences. It may be remarked that at least 18 common signatures (30 nts) were distributed along the length of the *rrs *(Additional file 10: Table S22). The most unique features were the positions of signatures with maximum frequency, which varied from 394-1170, implying higher susceptibility of the flanking regions to genetic modifications. In fact, such large core regions were also observed in *Bacillus *sp. [3]. It can be concluded that about 540 nts were shared by different *Clostridium *spp. and may represent those sequences which are highly conserved during evolution and may be designated as representatives of the genus *Clostridium*.

## Conclusions

*rrs *sequences have certain features such as nucleotide signatures (25 to 30 nts long) and unique RE digestion patterns which can be exploited for (re)defining bacterial taxonomy and phylogeny. This approach enabled us to develop species specific phylogenetic framework. These genetic tools allowed us to categorize *Clostridium *isolates which have been classified presently up to genus level into (i) well defined *Clostridium *spp. and (ii) as novel species. It also holds promise to reduce the number of *Clostridium *species represented by small populations. This integrated approach is quite sensitive and can be easily extended as a molecular tool for diagnosis of microbes used in food industries and health departments.

It appears as if we are reiterating (today in 2010) the statement made by Prof. C.L. Oakley, almost 6 decades ago: "Little is gained by multiplying genera to include rare or imperfectly-described forms, though increase in our knowledge may justify subdivision of the genus (*Clostridium*) in the future." [72].

## Methods

### Sequence data

The *rrs *sequences (> 1200 nts) chosen for this study corresponds to a total of 765 isolates belonging to the genus *Clostridium *(from RDP/NCBI sites: http://rdp.cme.msu.edu/; http://www.ncbi.nlm.nih.gov/. These included sequences belonging to isolates of *C. botulinum *- 128, *C. perfringens *- 92, *C. butyricum *- 32, *C. acetobutylicum *- 24, *C. beijerinckii *- 23, *C. novyi *- 17, *C. kluyveri *- 14, *C. pasteurianum *- 13, *C. sporogenes *- 11, *C. colicanis *and *C. sardiniense *- 9 each, *C. baratii*, *C. chauvoei*, *C. subterminale *and *C. tetani *- 8 each, *Clostridium *sp. - 179 and *Clostridium *spp. having members with low frequency (Table 1). The first fifteen sets of *Clostridium *species consisting of 404 *rrs *sequences were used as the reference species set for generating species specific: (i) phylogenetic framework, (ii) signatures and (iii) *in silico *RE digestion pattern. These tools were then used for identifying (i) *Clostridium *sp. up to species level and (ii) developing phylogenetic relationships among the other *Clostridium *species, which are represented by relatively smaller number of isolates.

### Phylogenetic Analyses

For phylogenetic analyses, *rrs *sequences of each of these 15 species were assembled and aligned using the multiple alignment program - ClustalX version 2.0.12 [73]. To estimate evolutionary distance, pairwise distances between all species were calculated with the DNADIST of the PHYLIP 3.69 package [3,74]. The resultant distance matrix was then used to draw a neighbour joining tree with the program NEIGHBOR. The program SEQBOOT [74] was used for statistical testing of the trees by resampling the dataset 1000 times. The trees were viewed through TreeView Version 1.6.6 [75], Phylodraw [76], MEGA [77] (Additional file 2: Figures S1,S2,S3,S4,S5,S6,S7,S8,S9,S1,S11,S12, S13,S14 and S15). For each of these 15 data sets, a 'guide' tree was made and 2-10 representative sequences were selected to develop framework sequences for the rest of the study. Thus a reference set of 56 *rrs *sequences was selected and regarded these as likely candidates that could give information about the organismal phylogeny (Figure 1). These framework sequences were validated by using 404 *rrs *sequences from 15 *Clostridium *species (Figures 2, 3, 4, 5, 6, 7 and 8). Subsequently, these 56 framework sequences were used to reclassify the *Clostridium *sp. (Figures 9, 10 and 11) and show phylogenetic relationships among other *Clostridium *spp. (with small populations) (Figures 12 and 13, Additional file 3: Figure S16).

In order to establish whether the 84 *rrs *sequences of *Clostridium *sp. which could not be clearly segregated among 15 *Clostridium *species (Figure 11), they were checked for their phylogenetic relationship with 80 *Clostridium *spp. (consisting of small populations) in the following manner. Initially, these 84 sequences (Figure 11) were reduced to 56 representative sequences on the basis of their distribution on the phylogenetic tree (Additional file 5: Figure S17). Two phylogenetic trees of 28 sequences each were drawn (Additional file 6: Figures S18 and S19) along with 83 sequences of 80 different *Clostridium *sp. (Additional file 4: Table S3).

### Restriction Enzyme Analyses

A total of 14 Type II REs http://rebase.neb.com/rebase/rebase.html were considered for these analyses: (i) 4 nts cutters - AluI (AG↓CT), BfaI (C↓TAG), DpnII (↓GATC), HaeIII (GG↓CC), RsaI (GT↓AC), Tru9I (T↓TAA), (ii) 6 nts cutters - BamHI (G↓GATCC), EcoRI (G↓AATTC), HindIII (A↓AGCTT), NruI (TCG↓CGA), SacI (GAGCT↓C), SmaI (CCC↓GGG), PstI (CTGCA↓G) and (iii) 8 nts cutter - NotI (GC↓GGCCGC) [3]. All the 15 *Clostridium *species considered for developing the phylogenetic framework were checked for all the 14 Type II RE using the online software: Restriction Mapper Version 3 http://www.restrictionmapper.org/. Sequences (one at a time) were entered in the restriction mapper site by removing all non base characters, results obtained were analyzed and consensus pattern was determined for each species depending upon its frequency of occurrence and the nucleotide fragment lengths of the sequences. The different *Clostridium *sp. and *Clostridium *spp. with low frequency of occurrence were checked for these consensus RE patterns.

### *Clostridium *Species-Specific Signatures

MEME has been used here for searching novel motifs or signatures in sets of biological sequences. MEME works by searching for repeated, ungapped sequence patterns that occur in the DNA or protein sequences [78,79]. MEME searches can be performed via the web server http://meme.sdsc.edu/meme4_4_0/intro.html and its mirror sites [79]. To successfully discover motifs with MEME, it is necessary to choose and prepare the input sequences carefully. Ideally, the sequences should be, 1000 base pairs long [80]. In our analysis, sequences of each of the 15 data sets in FASTA format were submitted group wise in MEME program Version 4.4.0 http://meme.sdsc.edu/meme4_4_0/intro.html. In order to obtain maximum number of motifs in our sequences, we modified default settings from 3-10 motifs. The default value of motif widths, set between 6 (minimum) and 50 (maximum) were modified and re-set between 25 and 30, respectively. We used default setting zero or one motif per sequence to get the occurrence of single motif which is distributed among the sequences. MEME search stops when this number of motifs has been found, or when none can be found with E-value less than 10000 http://meme.sdsc.edu/meme4_4_0/intro.html. Since, it posed a restriction of using 60000 characters at a time, we also used its stand alone format (MEME program Version 4.3.0) for our study for generating nucleotide signatures for data sets having greater than 60000 characters (for example in the cases of *C. botulinum *and *C. perfringens*, (Additional file 8: Figures S20,S21,S22 and S23, Additional file 11: Tables S23,S24,S25 and S26), where a large data was compressed for easy comprehension. Each of the 10 signatures (30 nts) (Additional file 9: Tables S5-S20) was checked through BioEdit [81] for its frequency of occurrence among all the sequences of all the 15 *Clostridium *spp. and the ones with highest frequency that did not appear in other *Clostridium *spp. were considered as unique to this species. In the case of 128 *rrs *sequences of *C. botulinum*, we could not detect any unique signatures. We thus divided the *C. botulinum *isolates into two groups of 83 and 45 on the basis of their distribution on the phylogenetic (guide) tree (Additional file 2: Figure S1). The *rrs *sequences of these two groups were then analyzed through MEME for identifying unique nucleotide signatures. These unique motifs were used as query sequence to validate the *Clostridium *spp. segregated through phylogenetic framework. The MEME result gives a graphic representation and Motifs in regular expression format.

## Competing interests

The authors declare that they have no competing interests.

## Authors' contributions

The author(s) have made the following declarations about their contributions: Conceived and designed the experiments: VCK. Performed the experiments: TM AB JJ PS NH. Analyzed the data: VCK. Wrote the paper: VCK.

## Supplementary Material

Additional file 1 **Table S1 *Clostridium *species**. File contains *rrs *sequences of *Clostridium *species which occurred with low frequency and the number of sequences used in this study http://rdp.cme.msu.edu/. Click here for file

Additional file 2 **Figures S1-S15 'Guide trees' for phylogenetic famework**. File contains 'Guide trees' for all the 15 *Clostridium *spp. used to develop phylogenetic framework sequences for this study. Click here for file

Additional file 3 **Figure S16 Phylogenetic tree of 16S rDNA of *Clostridium *spp. and Framework sequences**. File contains a neighbor - joining analysis of *rrs *sequences of Swine fecal bacterium, Clostridiaceae bacterium and unidentified eubacterium - 31 along with 56 of phylogenetic framework. Click here for file

Additional file 4 **Tables S2-S3 'Novel' and low frequency *Clostridium *spp**. File contains representative *rrs *sequences of unsegregated *Clostridium *sp. (novel) and *Clostridium *spp. with small population sizes. Click here for file

Additional file 5 **Figure S17 Phylogenetic tree of 84 16S rDNA sequences of 'novel' *Clostridium *species**. File contains a neighbor - joining analysis of 56 representative novel *Clostridium *sequences. Click here for file

Additional file 6 **Figures S18-S19 Phylogenetic tree of 16S rDNA of novel *Clostridium *spp. and low frequency *Clostridium *spp**. File contains a neighbor - joining analysis performed on the *rrs *sequences of novel *Clostridium *sp. (Additional file 4: Table S2) 56 along with 83 representatives of *Clostridium *sp. known to occur at low frequency (Additional file 4: Table S3). Click here for file

Additional file 7 **Table S4 RE sites with low frequency**. File contains low frequency *in silico *Restriction Enzymes cut sites in *rrs *sequences of different *Clostridium *spp. Click here for file

Additional file 8 **Figures S20-S36 Regular expression diagram**. File contains regular expression diagram of signatures (nucleotides) of *rrs *sequences of 15 *Clostridium *spp. obtained through MEME suite. Click here for file

Additional file 9 **Table S5-S21 Motifs for 15 *Clostridium *spp**. File represents motifs obtained for *Clostridium botulinum *(128 *rrs *sequences) through MEME suite and the frequency of their occurrence across other *Clostridium *spp. using BioEdit. Click here for file

Additional file 10 **Table S22 Nucleotide signatures**. File contains nucleotide signatures of *rrs *sequences common to *Clostridium *sp. used for developing framework sequences. Click here for file

Additional file 11 **Tables S23-S26 Representative motifs for *Clostridium botulinum *and *C. perfringens***. File contains details of *rrs *sequences of *Clostridium botulinum *and *C. perfringens *used for drawing Regular Expression Diagram of signatures presented in Additional file 8: Figures S20-S23. Click here for file
