# Supplementary material for: Analysis of the unexplored features of rrs (16S rDNA) of the Genus Clostridium
Source: BMC Genomics. 2011 Jan 11;12:18. doi: 10.1186/1471-2164-12-18 (PMC3024285; doi:10.1186/1471-2164-12-18)
Supplement: Additional file 1 — Table S1 Clostridium species. File contains rrs sequences of Clostridium species which occurred with low frequency and the number of sequences used in this study http://rdp.cme.msu.edu/. [file 1471-2164-12-18-S1.DOC]

| **Table S1: 16S r DNA sequences of *Clostridium* species which occurred with low frequency and the number of sequences used in this study (**[**http://rdp.cme.msu.edu/**](http://rdp.cme.msu.edu/)**).** | | |
| --- | --- | --- |
| **S. No.** | Organism | No. of sequences |
| **1** | *Clostridium paraputrificum* | 5 |
| **2** | *C. diolis* | 4 |
| **3** | *C. thiosulfatireducens* | 4 |
| **4** | *C. tyrobutyricum* | 4 |
| **5** | *C. bowmanii* | 3 |
| **6** | *C. estertheticum* | 3 |
| **7** | *C. intestinale* | 3 |
| **8** | *C. ljungdahlii* | 3 |
| **9** | *C. neonatale* | 3 |
| **10** | *C. saccharobutylicum* | 3 |
| **11** | *C. septicum* | 3 |
| **12** | *C. tetanomorphum* | 3 |
| **13** | *C. algidicarnis* | 2 |
| **14** | *C. aurantibutyricum* | 2 |
| **15** | *C. cellulovorans* | 2 |
| **16** | *C. cochlearium* | 2 |
| **17** | *C. disporicum* | 2 |
| **18** | *C. drakei* | 2 |
| **19** | *C. fallax* | 2 |
| **20** | *C. felsineum* | 2 |
| **21** | *C. frigoris* | 2 |
| **22** | *C. gasigenes* | 2 |
| **23** | *C. haemolyticum* | 2 |
| **24** | *C. limosum* | 2 |
| **25** | *C. puniceum* | 2 |
| **26** | *C. putrefaciens* | 2 |
| **27** | *C. roseum* | 2 |
| **28** | *C. sartagoforme* | 2 |
| **29** | *C. tertium* | 2 |
| **30** | *C. thermopalmarium* | 2 |
| **31** | *C. acetireducens* | 1 |
| **32** | *C. acidisoli* | 1 |
| **33** | *C. aciditolerans* | 1 |
| **34** | *C. aestuarii* | 1 |
| **35** | *C. akagii* | 1 |
| **36** | *C. algoriphilum* | 1 |
| **37** | *C. argentinense* | 1 |
| **38** | *C. autoethanogenum* | 1 |
| **39** | *C. bovipellis* | 1 |
| **40** | *C. caenicola* | 1 |
| **41** | *C. caliptrosporum* | 1 |
| **42** | *C. carnis* | 1 |
| **43** | *C. celatum* | 1 |
| **44** | *C. chartatabidum* | 1 |
| **45** | *C. chromoreductans* | 1 |
| **46** | *C. collagenovorans* | 1 |
| **47** | *C. corinoforum* | 1 |
| **48** | *C. crotonatovorans* | 1 |
| **49** | *C. cylindrosporum* | 1 |
| **50** | *C. favososporum* | 1 |
| **51** | *C. frigidicarnis* | 1 |
| **52** | *C. frigoriphilum* | 1 |
| **53** | *C. ganghwense* | 1 |
| **54** | *C. grantii* | 1 |
| **55** | *C. histolyticum* | 1 |
| **56** | *C. homopropionicum* | 1 |
| **57** | *C. lacusfryxellense* | 1 |
| **58** | *C. longisporum* | 1 |
| **59** | *C. lundense* | 1 |
| **60** | *C. magnum* | 1 |
| **61** | *C. mesophilum* | 1 |
| **62** | *C. nitrophenolicum* | 1 |
| **63** | *C. pascui* | 1 |
| **64** | *C. peptidivorans* | 1 |
| **65** | *C. proteolyticum* | 1 |
| **66** | *C. proteolyticus* | 1 |
| **67** | *C. psychrophilum* | 1 |
| **68** | *C. quinii* | 1 |
| **69** | *C. ragsdalei* | 1 |
| **70** | *C. saccharoperbutylicum* | 1 |
| **71** | *C. scatologenes* | 1 |
| **72** | *C. schirmacherense* | 1 |
| **73** | *C. sulfidigenes* | 1 |
| **74** | *C. taeniosporum* | 1 |
| **75** | *C. tagluense* | 1 |
| **76** | *C. tepidiprofundum* | 1 |
| **77** | *C. thermobutyricum* | 1 |
| **78** | *C. tunisiense* | 1 |
| **79** | *C. uliginosum* | 1 |
| **80** | *C. vincentii* | 1 |
| **81** | Clostridiaceae bacterium^a^ | 8 |
| **82** | Clostridiales bacterium | 1 |
| **83** | *Eubacterium budayi* | 2 |
| **84** | *Eubacterium combesii* | 1 |
| **85** | *Eubacterium moniliforme* | 1 |
| **86** | *Eubacterium multiforme* | 1 |
| **87** | *Eubacterium nitritogenes* | 1 |
| **88** | Unidentified eubacterium^a^ | 8 |
| **89** | Bacterium | 4 |
| **90** | Swine fecal bacterium^a^ | 15 |
| **91** | Rumen bacterium | 6 |
| **92** | Intestinal bacterium | 2 |
| **93** | Swine manure bacterium | 4 |
| **94** | Red Sea bacterium | 1 |
|  | TOTAL | 182 |
| **^a^The 31 sequences belonging to three groups were not included for detailed studies.** | | |
