# Supplementary material for: Analysis of the unexplored features of rrs (16S rDNA) of the Genus Clostridium
Source: BMC Genomics. 2011 Jan 11;12:18. doi: 10.1186/1471-2164-12-18 (PMC3024285; doi:10.1186/1471-2164-12-18)
Supplement: Additional file 2 — Figures S1-S15 'Guide trees' for phylogenetic famework. File contains 'Guide trees' for all the 15 Clostridium spp. used to develop phylogenetic framework sequences for this study. [file 1471-2164-12-18-S2.PDF]

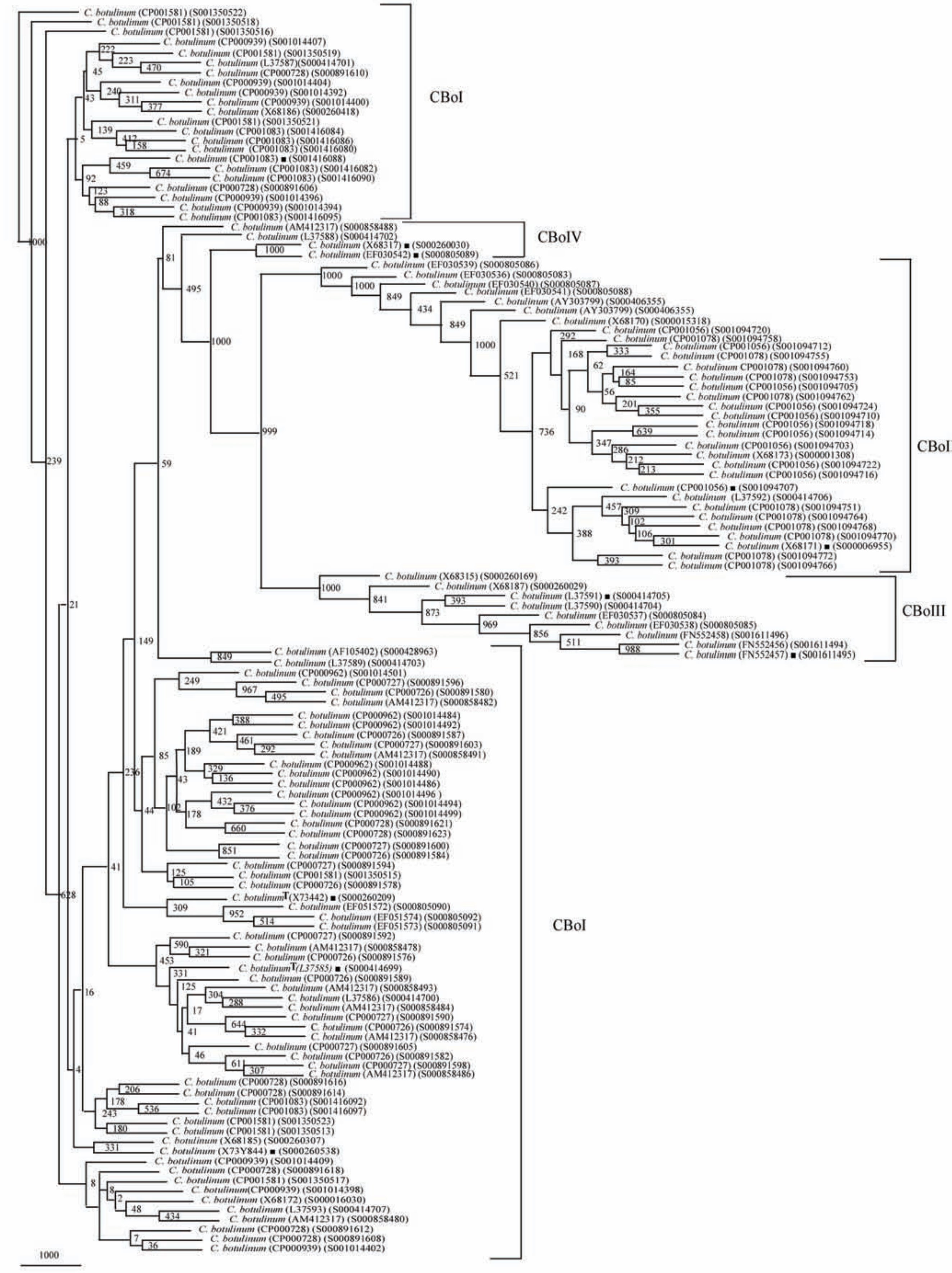

Figure S1 Phylogenetic tree based on 128, 16S rDNA sequences of *Clostridium botulinum*.
A neighbor – joining analysis with Jukes–Cantor correction and bootstrap support was performed on the gene sequences. Bootstrap values are given at nodes. Sequences marked by filled square are the ones considered as framework in the study whereas type strains are indicated by 'T' as superscript. Values in parentheses are accession number (RDP and NCBI) (http://rdp.cme.msu.edu/ and http://www.ncbi.nlm.nih.gov/).

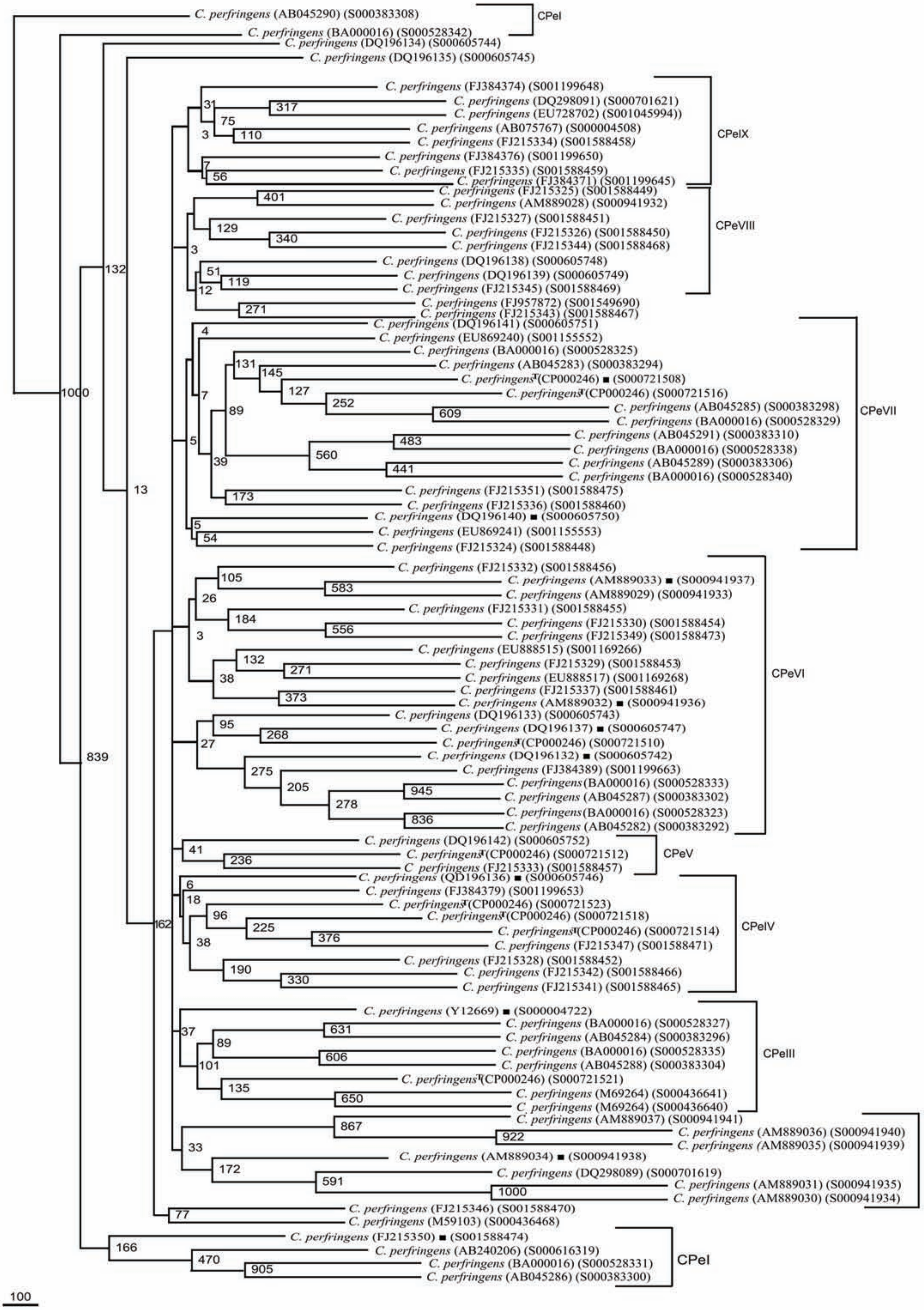

Figure S2 Phylogenetic tree based on 92, 16S rDNA sequences of *Clostridium perfringens*.

A neighbor – joining analysis with Jukes–Cantor correction and bootstrap support was performed on the gene sequences. Bootstrap values are given at nodes. Sequences marked by filled square are the ones considered as framework in the study whereas type strains are indicated by 'T' as superscript. Values in parentheses are accession numbers (RDP and NCBI) (http://rdp.cme.msu.edu/ and http://www.ncbi.nlm.nih.gov/).

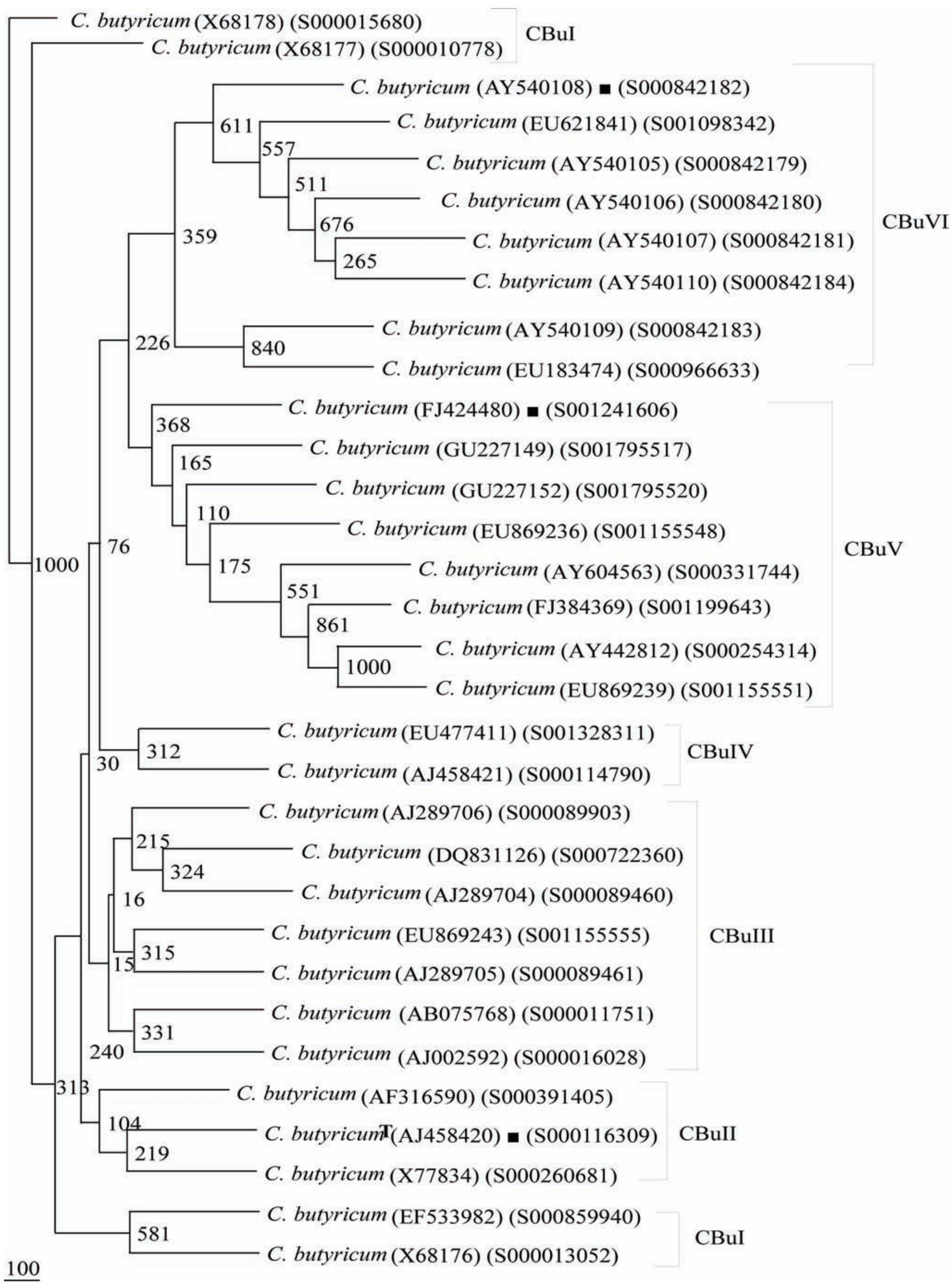

Figure S3 Phylogenetic tree based on 32, 16S rDNA sequences of *Clostridium butyricum*.
A neighbor – joining analysis with Jukes–Cantor correction and bootstrap support was performed on the gene sequences. Bootstrap values are given at nodes. Sequences marked by filled square are the ones considered as framework in the study whereas type strains are indicated by 'T' as superscript. Values in parentheses are accession numbers (RDP and NCBI) (http://rdp.cme.msu.edu/ and http://www.ncbi.nlm.nih.gov/).

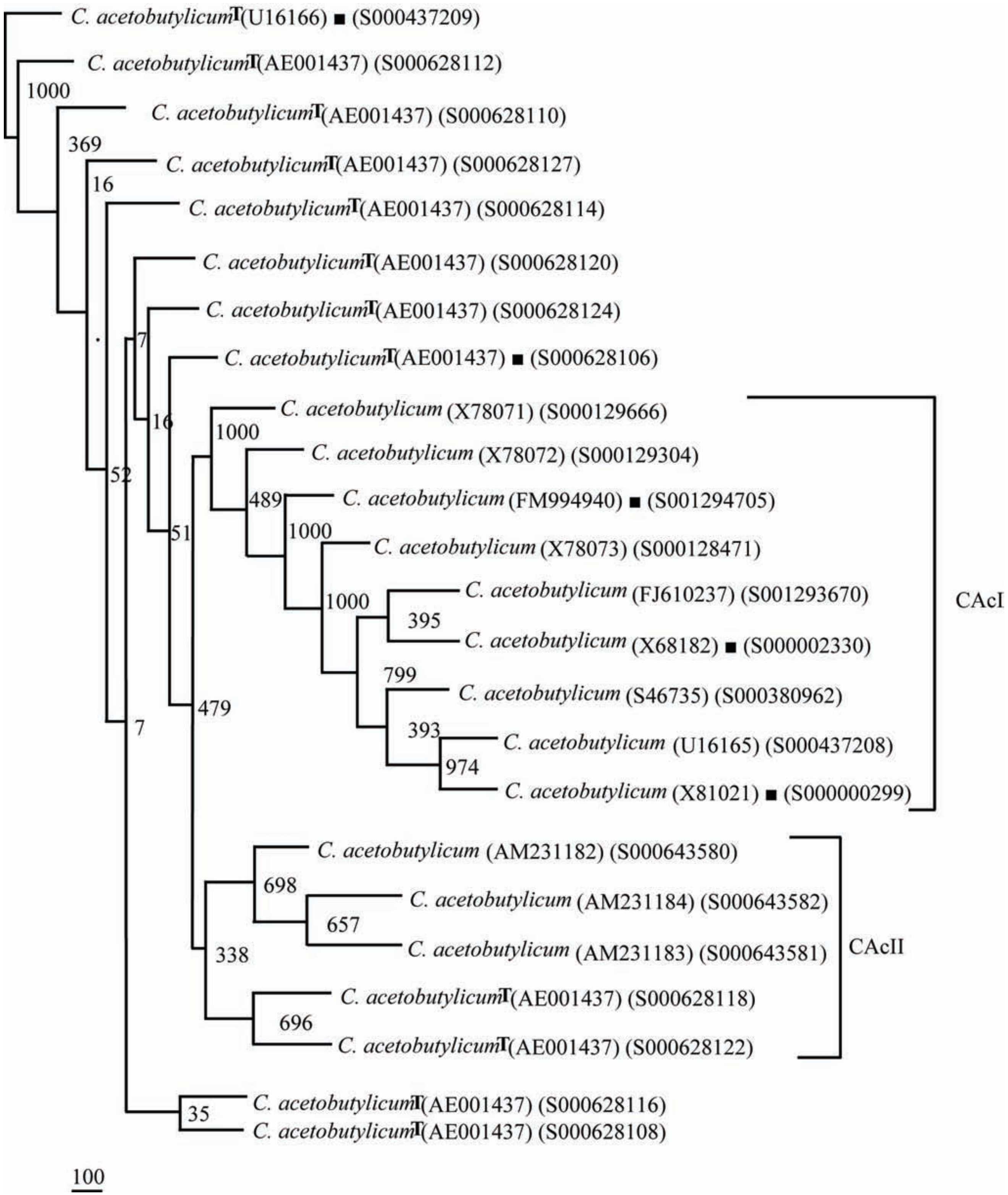

Figure S4 Phylogenetic tree based on 24, 16S rDNA sequences of *Clostridium acetobutylicum*.
A neighbor – joining analysis with Jukes–Cantor correction and bootstrap support was performed on the gene sequences. Bootstrap values are given at nodes. Sequences marked by filled square are the ones considered as framework in the study whereas type strains are indicated by 'T' as superscript. Values in parentheses are accession numbers (RDP and NCBI) (http://rdp.cme.msu.edu/ and http://www.ncbi.nlm.nih.gov/).

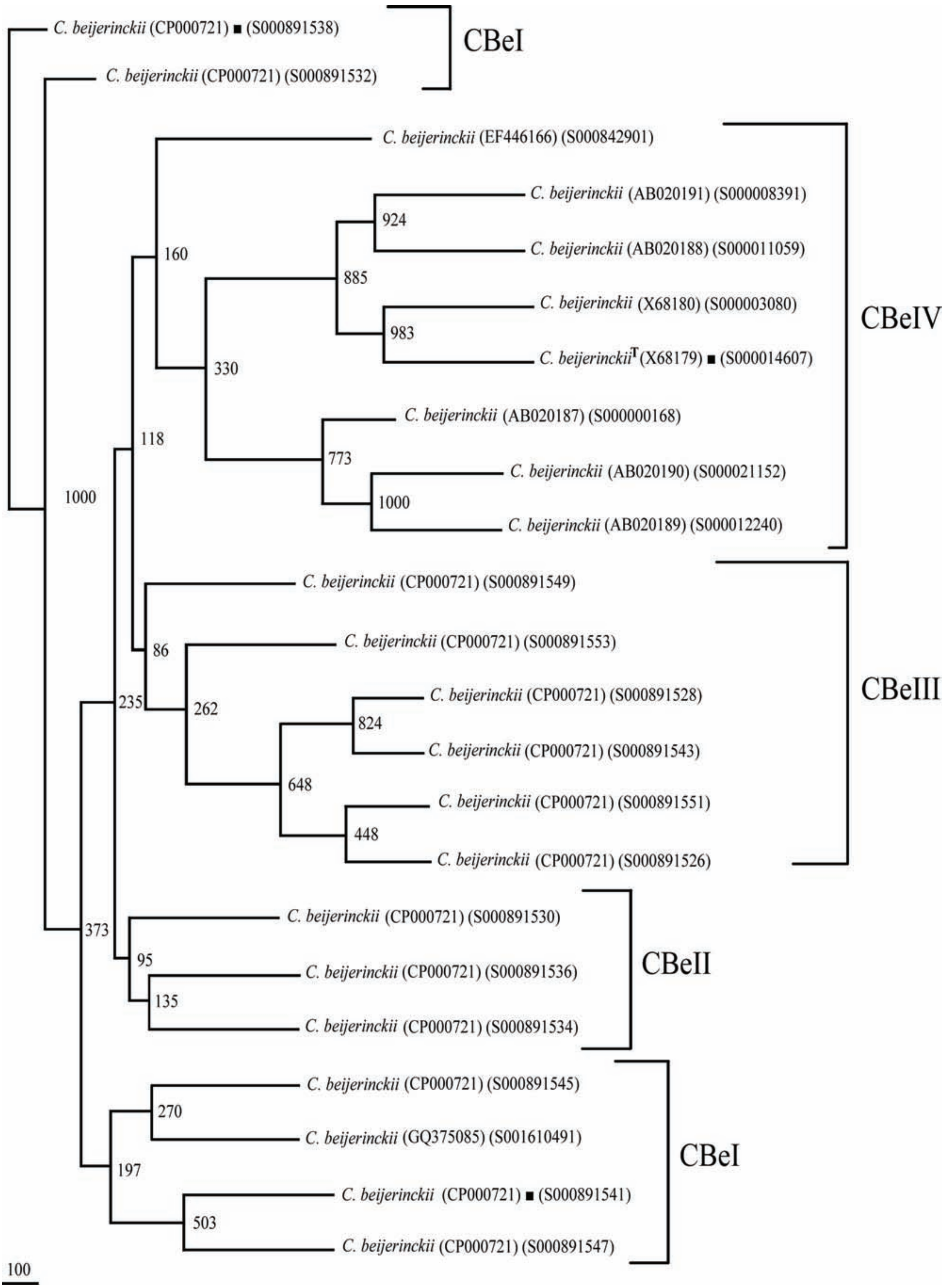

Figure S5 Phylogenetic tree based on 23, 16S rDNA sequences of *Clostridium beijerinckii*.
A neighbor – joining analysis with Jukes–Cantor correction and bootstrap support was performed on the gene sequences. Bootstrap values are given at nodes. Sequences marked by filled square are the ones considered as framework in the study whereas type strains are indicated by ‘T’ as superscript. Values in parentheses are accession numbers (RDP and NCBI) (http://rdp.cme.msu.edu/ and http://www.ncbi.nlm.nih.gov/).

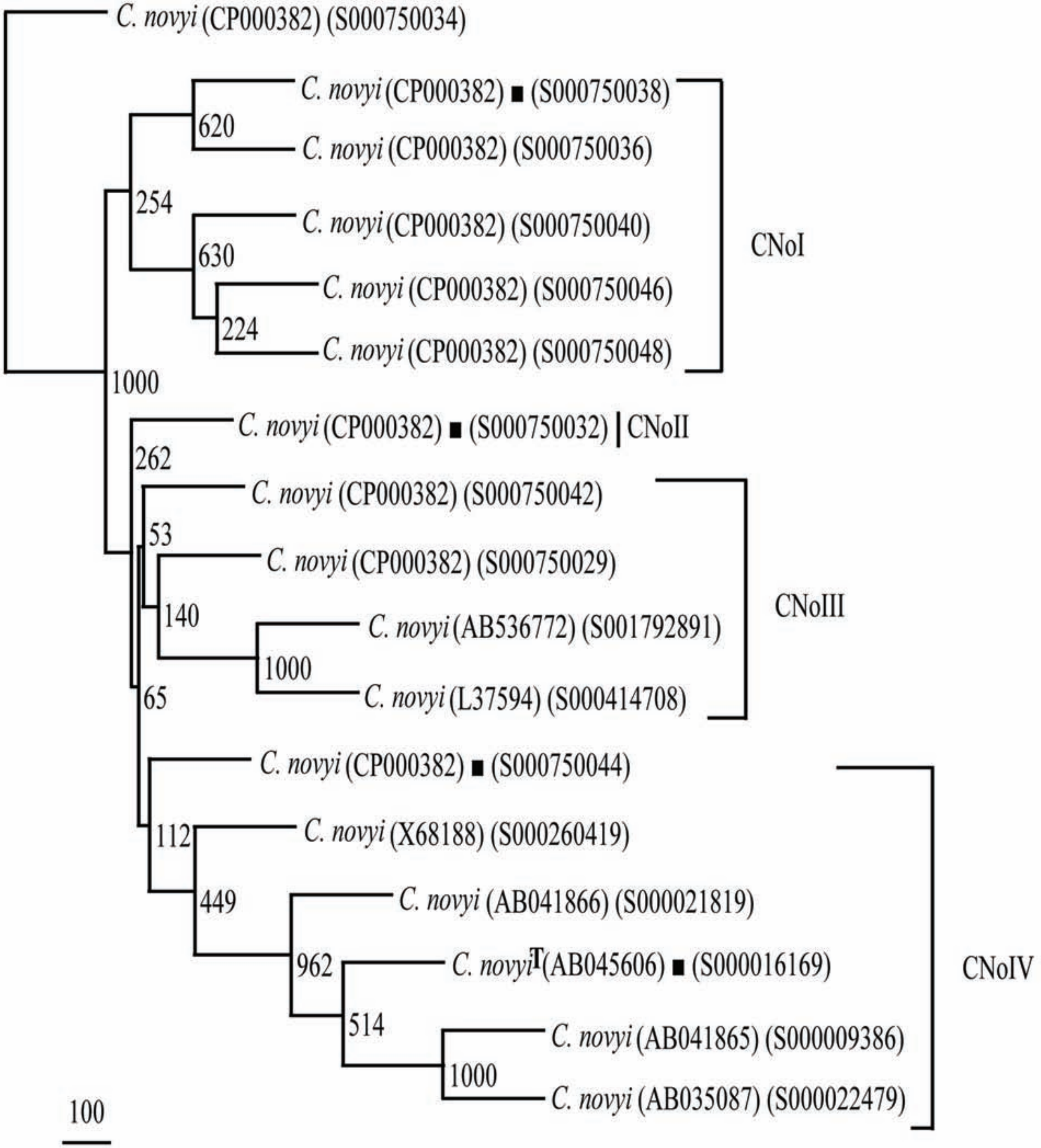

Figure S6 Phylogenetic tree based on 17, 16S rDNA sequences of *Clostridium novyi*.

A neighbor – joining analysis with Jukes–Cantor correction and bootstrap support was performed on the gene sequences. Bootstrap values are given at nodes. Sequences marked by filled square are the ones considered as framework in the study whereas type strains are indicated by ‘T’ as superscript. Values in parentheses are accession numbers (RDP and NCBI) (http://rdp.cme.msu.edu/ and http://www.ncbi.nlm.nih.gov/).

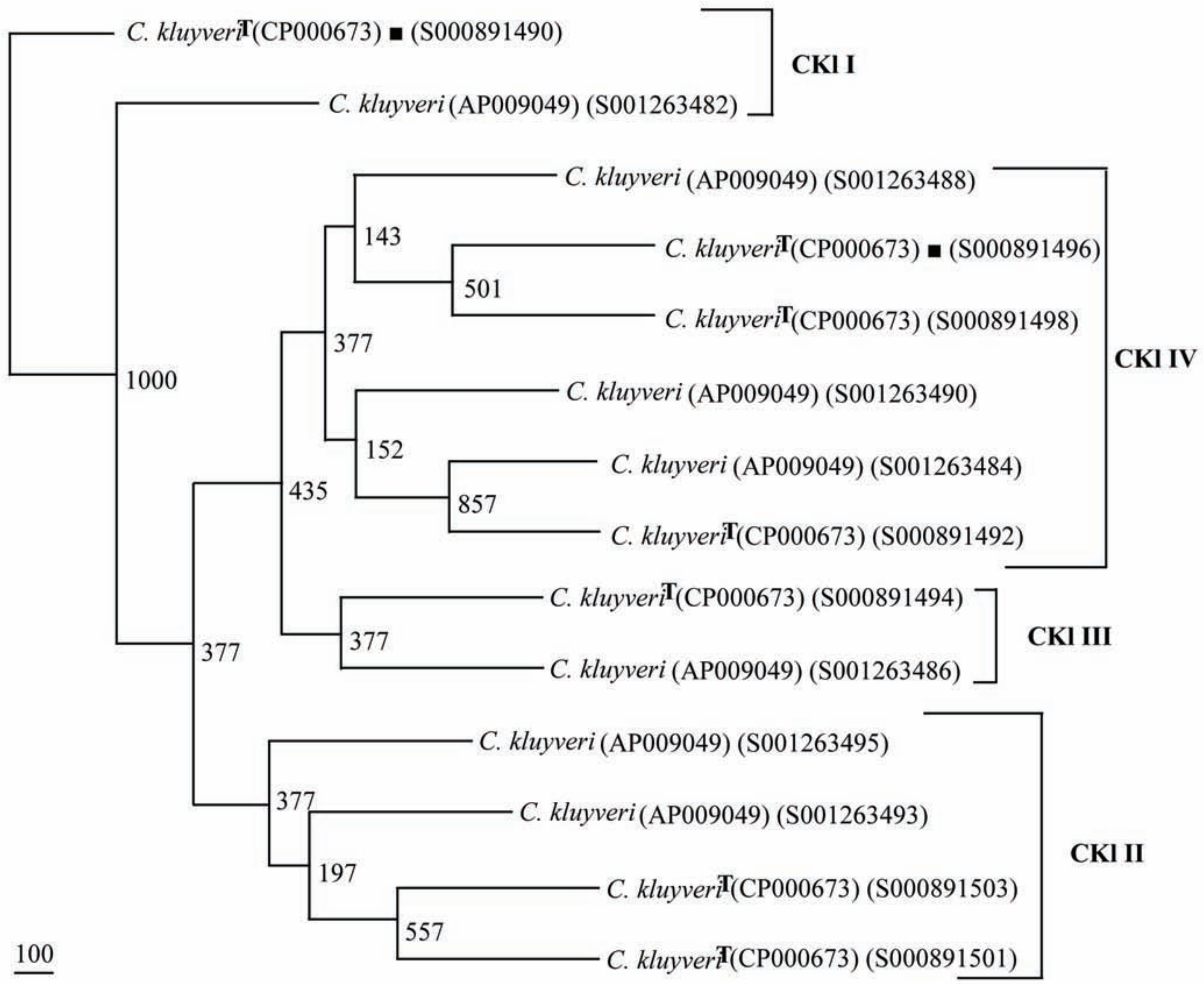

Figure S7 Phylogenetic tree based on 14, 16S rDNA sequences of *Clostridium kluyveri*.

A neighbor – joining analysis with Jukes–Cantor correction and bootstrap support was performed on the gene sequences. Bootstrap values are given at nodes. Sequences marked by filled square are the ones considered as framework in the study whereas type strains are indicated by 'T' as superscript. Values in parentheses are accession numbers (RDP and NCBI) (http://rdp.cme.msu.edu/ and http://www.ncbi.nlm.nih.gov/).

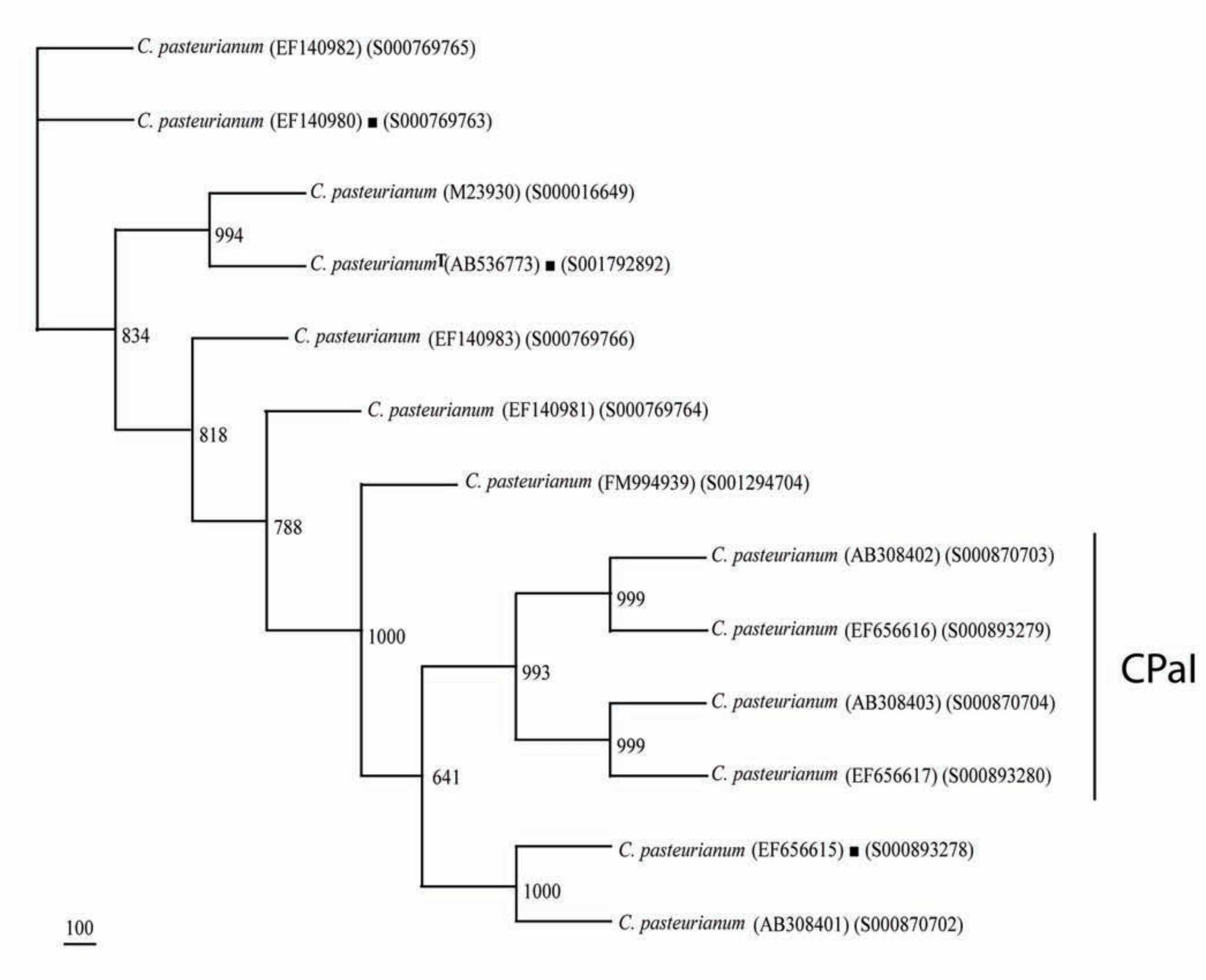

Figure S8 Phylogenetic tree based on 13, 16S rDNA sequences of *Clostridium pasteurianum*.

A neighbor – joining analysis with Jukes–Cantor correction and bootstrap support was performed on the gene sequences. Bootstrap values are given at nodes. Sequences marked by filled square are the ones considered as framework in the study whereas type strains are indicated by 'T' as superscript. Values in parentheses are accession numbers (RDP and NCBI) (http://rdp.cme.msu.edu/ and http://www.ncbi.nlm.nih.gov/).

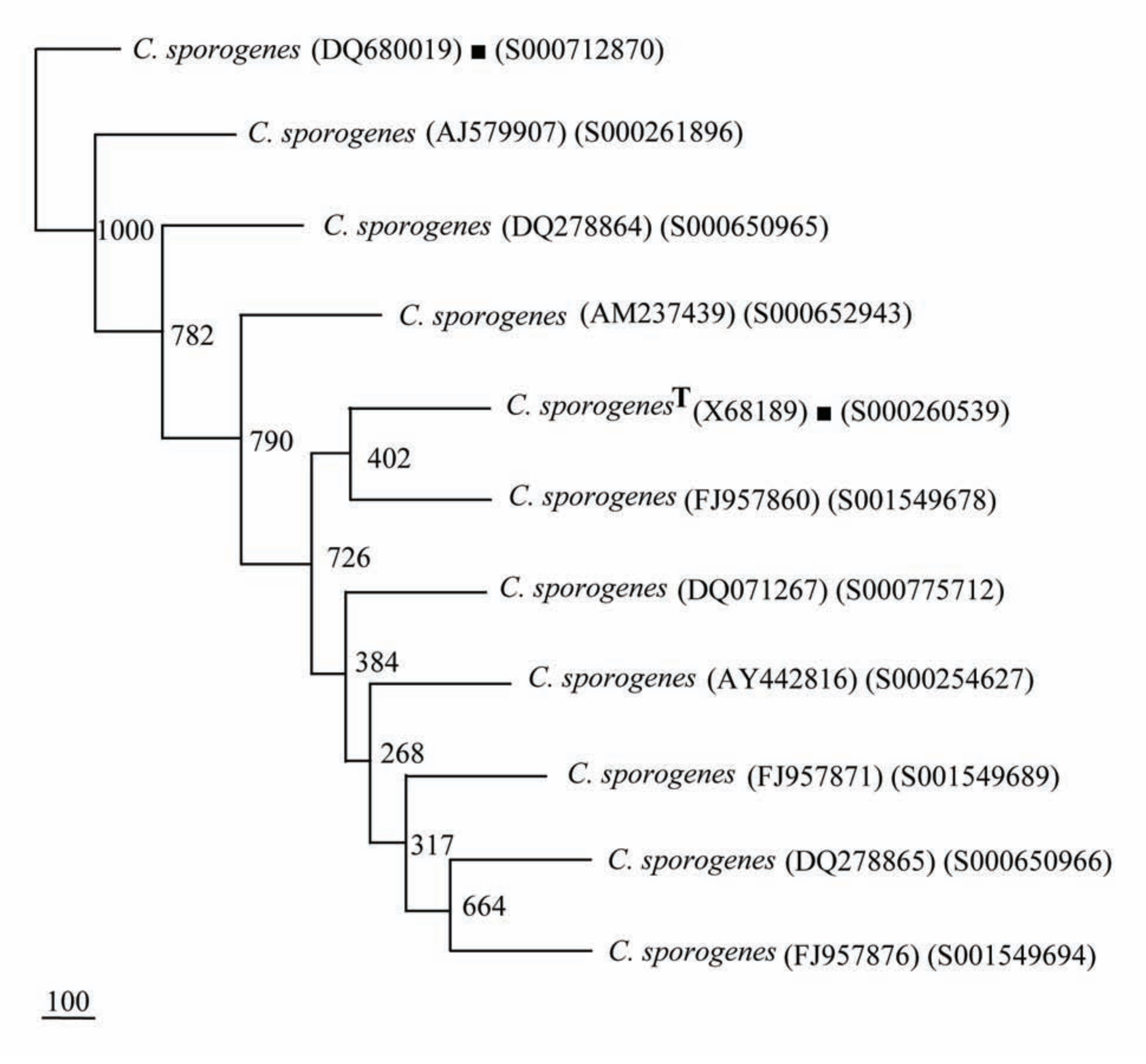

Figure S9 Phylogenetic tree based on 11, 16S rDNA sequences of *Clostridium sporogenes*.
A neighbor – joining analysis with Jukes–Cantor correction and bootstrap support was performed on the gene sequences. Bootstrap values are given at nodes. Sequences marked by filled square are the ones considered as framework in the study whereas type strains are indicated by 'T' as superscript. Values in parentheses are accession numbers (RDP and NCBI) (http://rdp.cme.msu.edu/ and http://www.ncbi.nlm.nih.gov/).

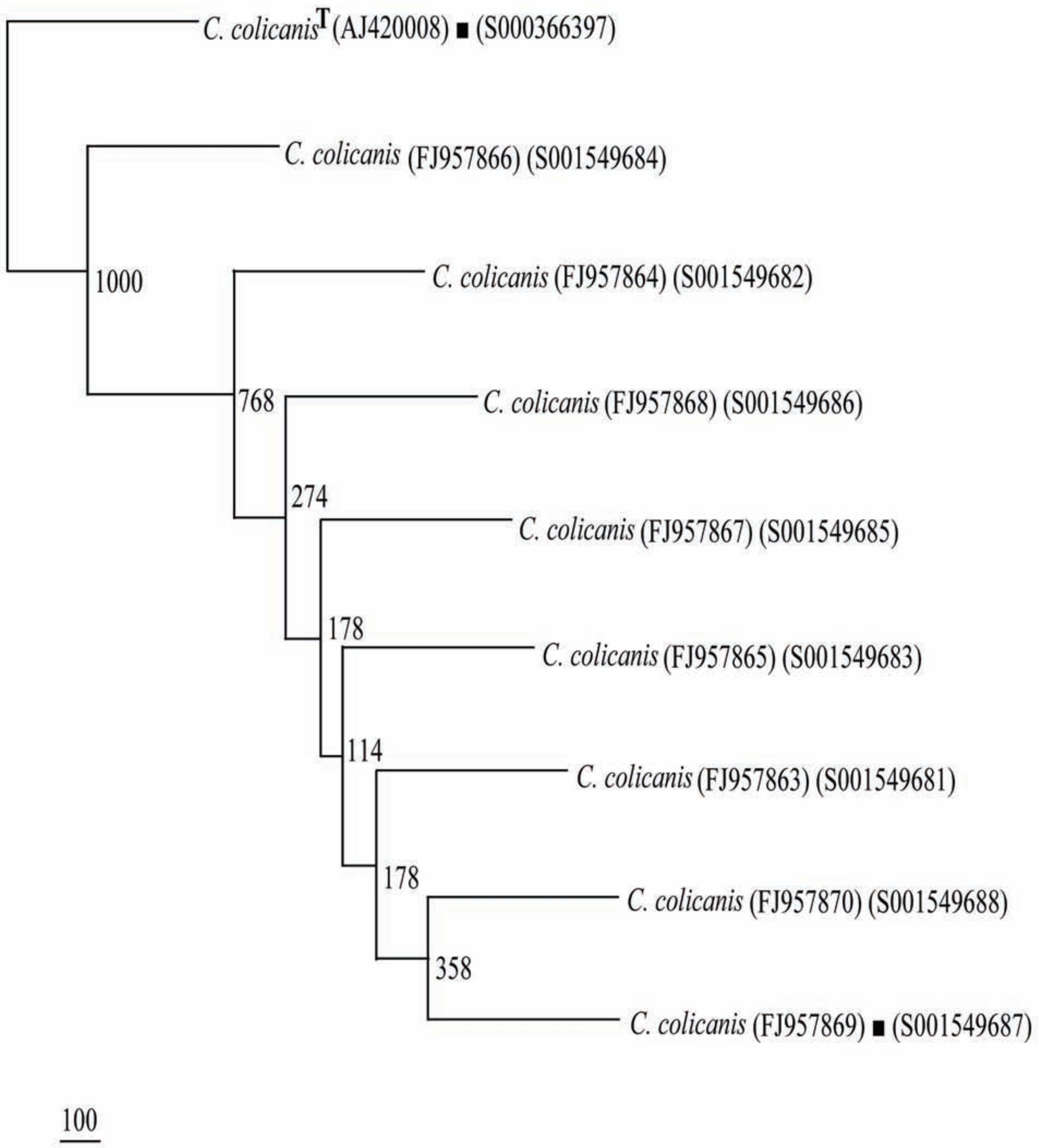

Figure S10 Phylogenetic tree based on 9, 16S rDNA sequences of *Clostridium colicanis*.
A neighbor – joining analysis with Jukes–Cantor correction and bootstrap support was performed on the gene sequences. Bootstrap values are given at nodes. Sequences marked by filled square are the ones considered as framework in the study whereas type strains are indicated by 'T' as superscript. Values in parentheses are accession numbers (RDP and NCBI) (http://rdp.cme.msu.edu/ and http://www.ncbi.nlm.nih.gov/).

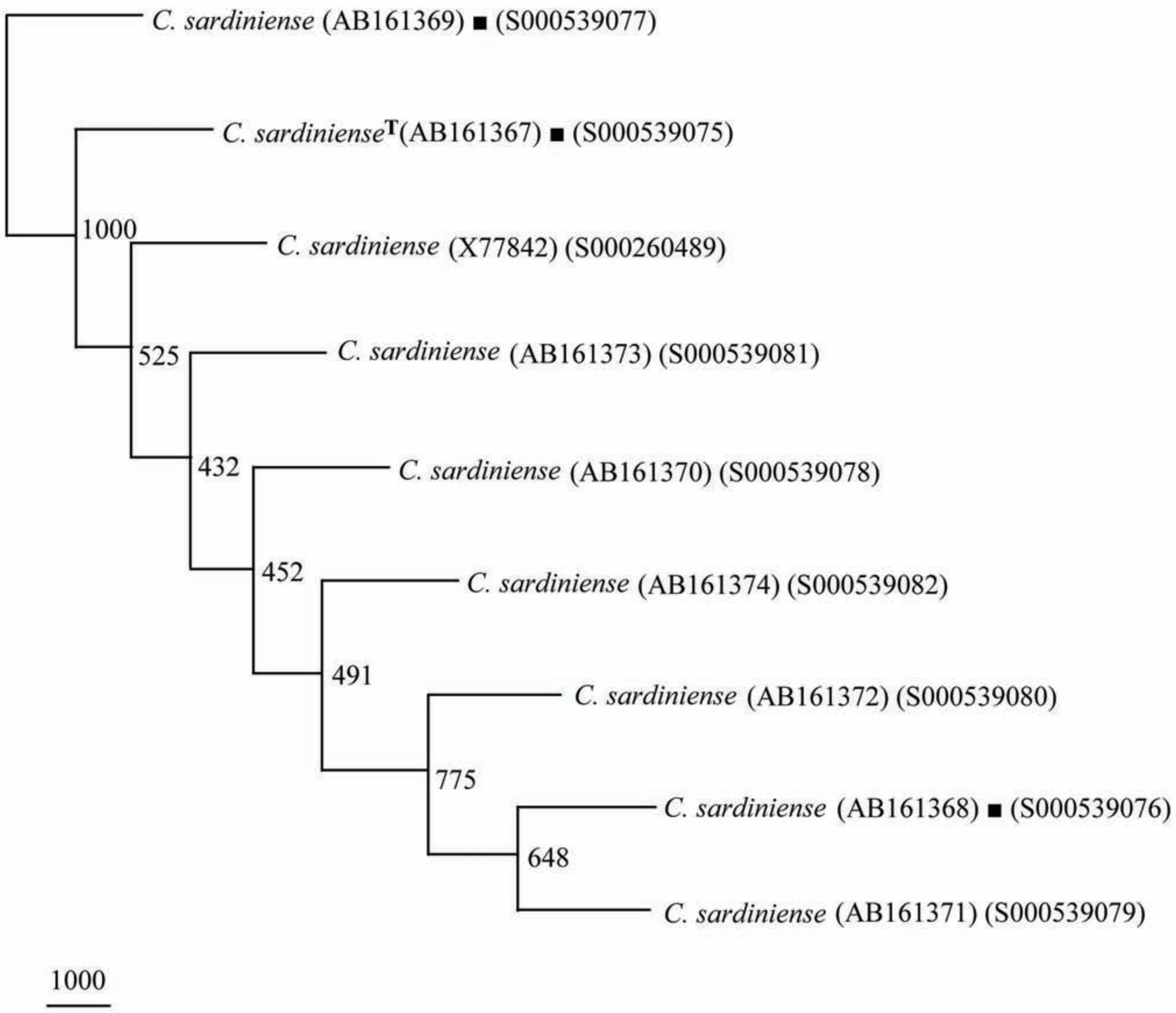

Figure S11 Phylogenetic tree based on 9, 16S rDNA sequences of *Clostridium sardiniense*.

A neighbor – joining analysis with Jukes–Cantor correction and bootstrap support was performed on the gene sequences. Bootstrap values are given at nodes. Sequences marked by filled square are the ones considered as framework in the study whereas type strains are indicated by 'T' as superscript. Values in parentheses are accession numbers (RDP and NCBI) (http://rdp.cme.msu.edu/ and http://www.ncbi.nlm.nih.gov/).

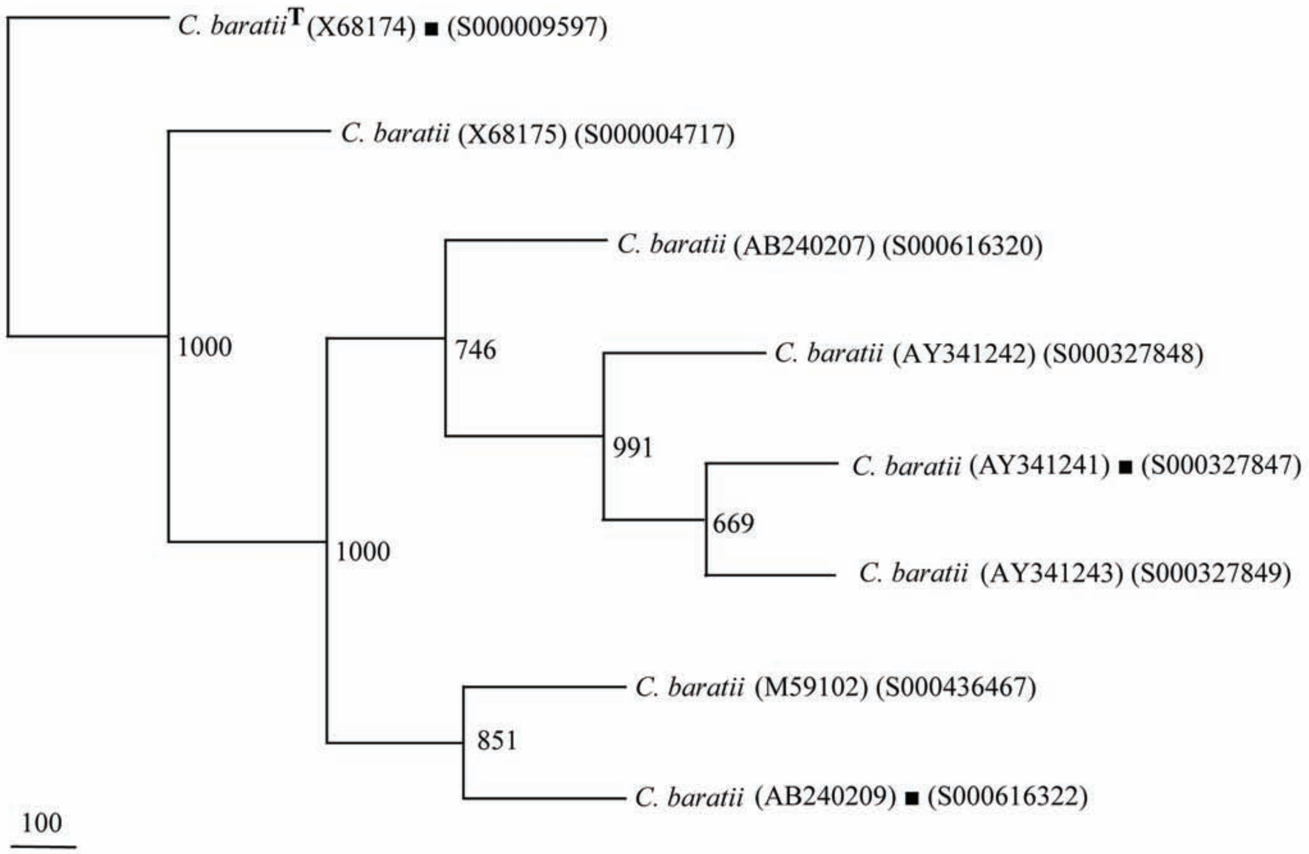

Figure S12 Phylogenetic tree based on 8, 16S rDNA sequences of *Clostridium baratii*.
A neighbor – joining analysis with Jukes–Cantor correction and bootstrap support was performed on the gene sequences. Bootstrap values are given at nodes. Sequences marked by filled square are the ones considered as framework in the study whereas type strains are indicated by 'T' as superscript.Values in parentheses are accession numbers (RDP and NCBI) (http://rdp.cme.msu.edu/ and http://www.ncbi.nlm.nih.gov/).

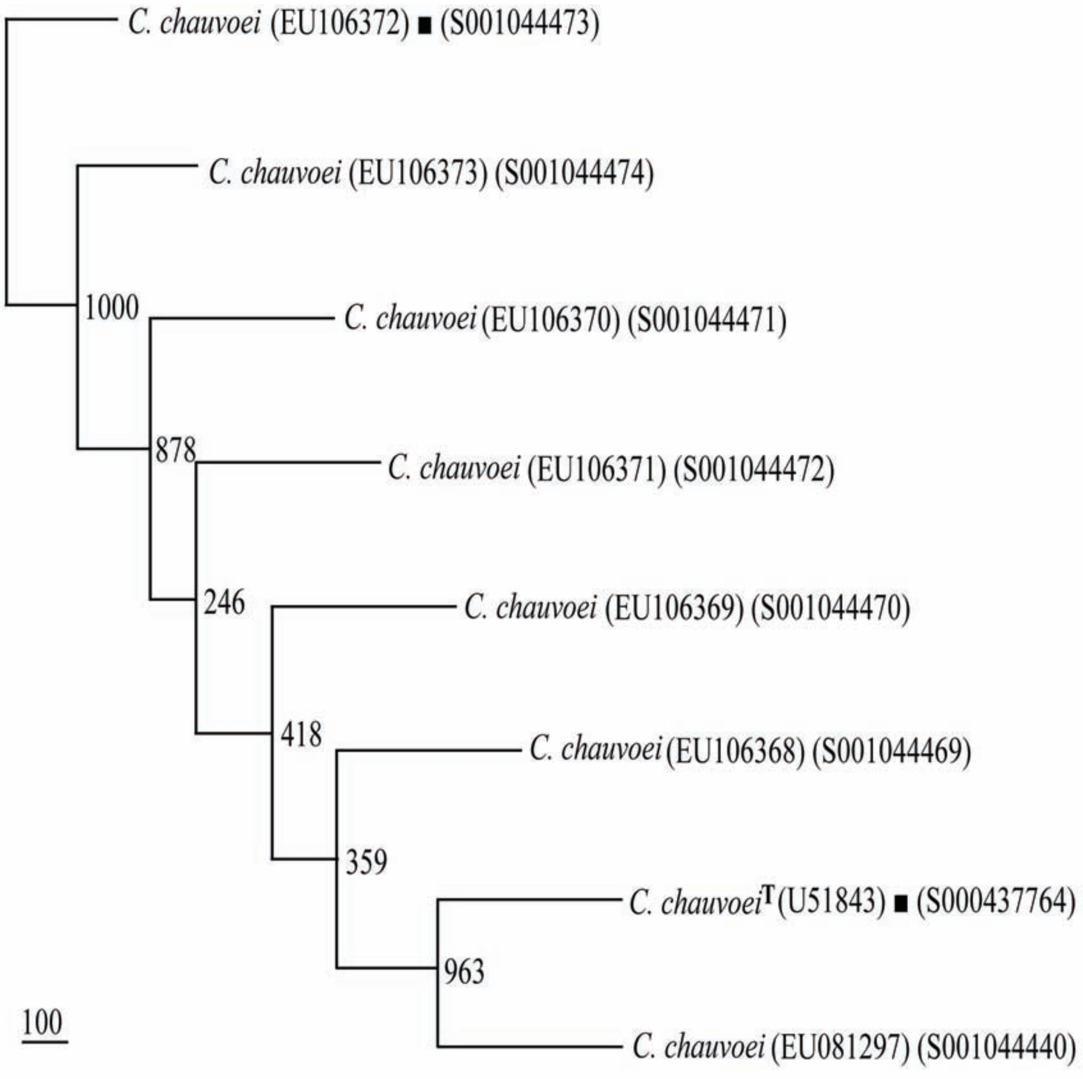

Figure S13 Phylogenetic tree based on 8, 16S rDNA sequences of *Clostridium chauvoei*. A neighbor – joining analysis with Jukes–Cantor correction and bootstrap support was performed on the gene sequences. Bootstrap values are given at nodes. Sequences marked by filled square are the ones considered as framework in the study whereas type strains are indicated by ‘T’ as superscript.Values in parentheses are accession numbers (RDP and NCBI) (http://rdp.cme.msu.edu/ and http://www.ncbi.nlm.nih.gov/).

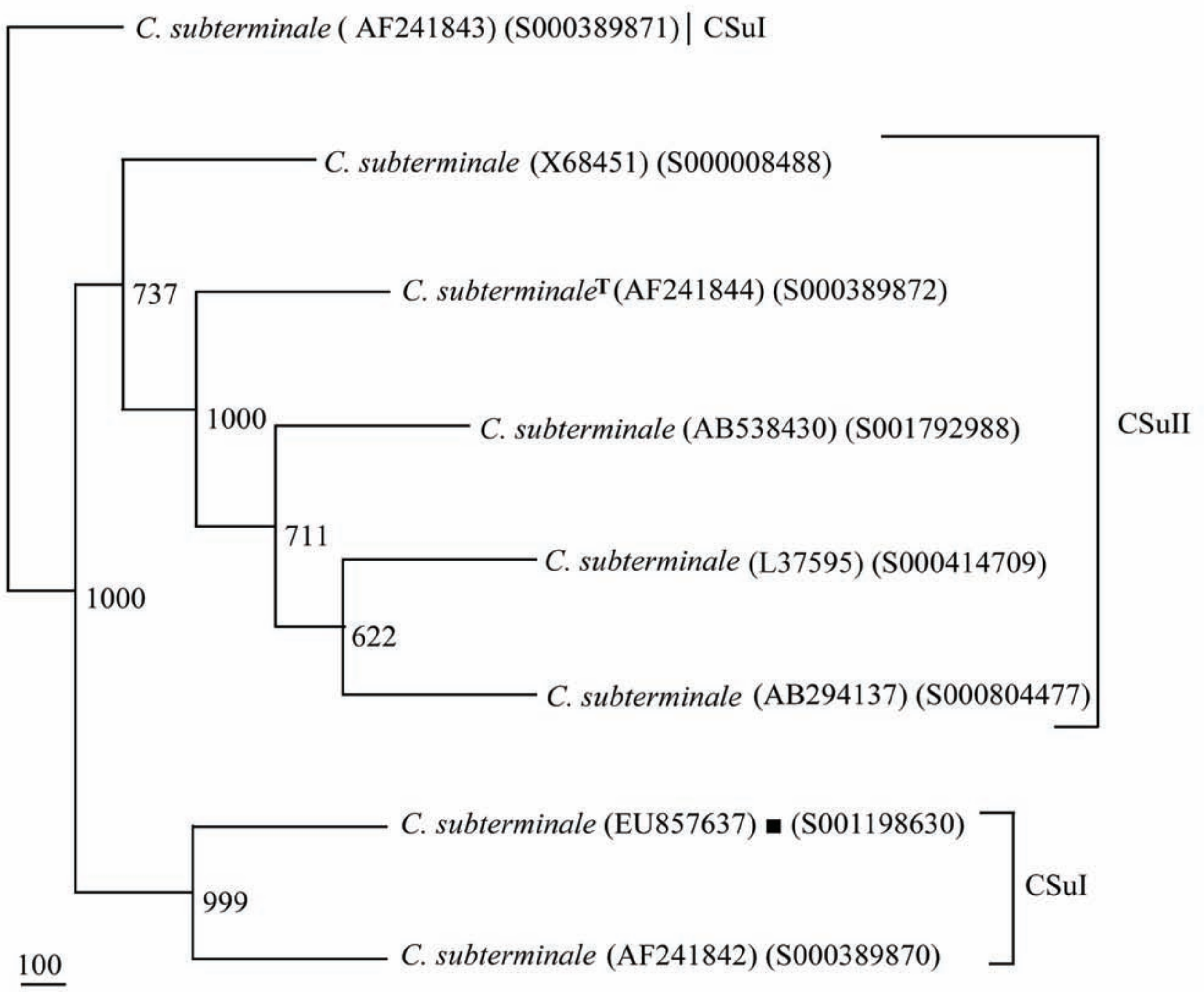

Figure S14 Phylogenetic tree based on 8, 16S rDNA sequences of *Clostridium subterminale*.
A neighbor – joining analysis with Jukes–Cantor correction and bootstrap support was performed on the gene sequences. Bootstrap values are given at nodes. Sequences marked by filled square are the ones considered as framework in the study whereas type strains are indicated by 'T' as superscript. Values in parentheses are accession numbers (RDP and NCBI) (http://rdp.cme.msu.edu/ and http://www.ncbi.nlm.nih.gov/).

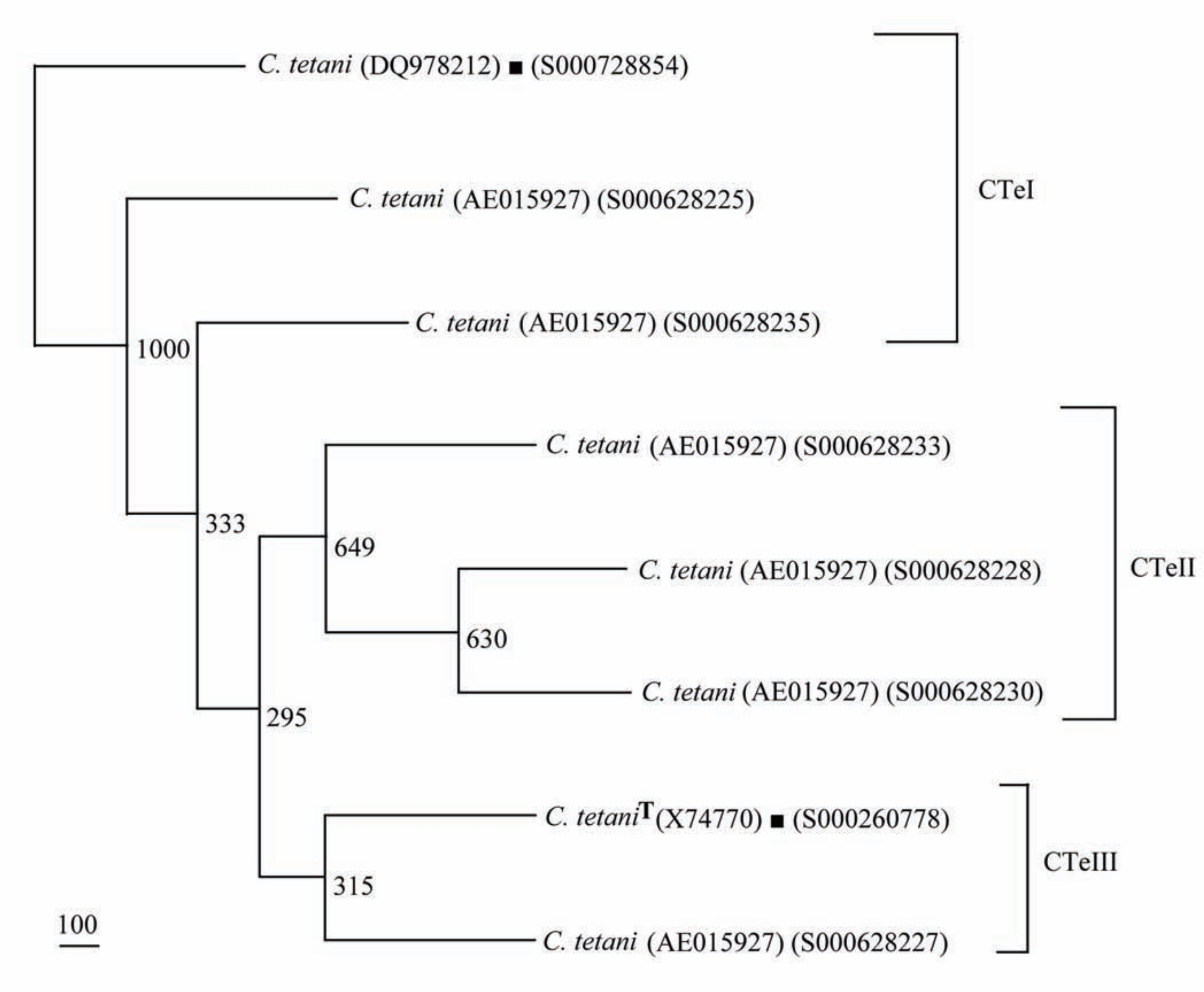

Figure S15 Phylogenetic tree based on 8, 16S rDNA sequences of *Clostridium tetani*.
A neighbor – joining analysis with Jukes–Cantor correction and bootstrap support was performed on the gene sequences. Bootstrap values are given at nodes. Sequences marked by filled square are the ones considered as framework in the study whereas type strains are indicated by 'T' as superscript.Values in parentheses are accession numbers (RDP and NCBI) (http://rdp.cme.msu.edu/ and http://www.ncbi.nlm.nih.gov/).
