# Supplementary material for: Analysis of the unexplored features of rrs (16S rDNA) of the Genus Clostridium
Source: BMC Genomics. 2011 Jan 11;12:18. doi: 10.1186/1471-2164-12-18 (PMC3024285; doi:10.1186/1471-2164-12-18)
Supplement: Additional file 3 — Figure S16 Phylogenetic tree of 16S rDNA of Clostridium spp. and Framework sequences. File contains a neighbor - joining analysis of rrs sequences of Swine fecal bacterium, Clostridiaceae bacterium and unidentified eubacterium - 31 along with 56 of phylogenetic framework. [file 1471-2164-12-18-S3.PDF]

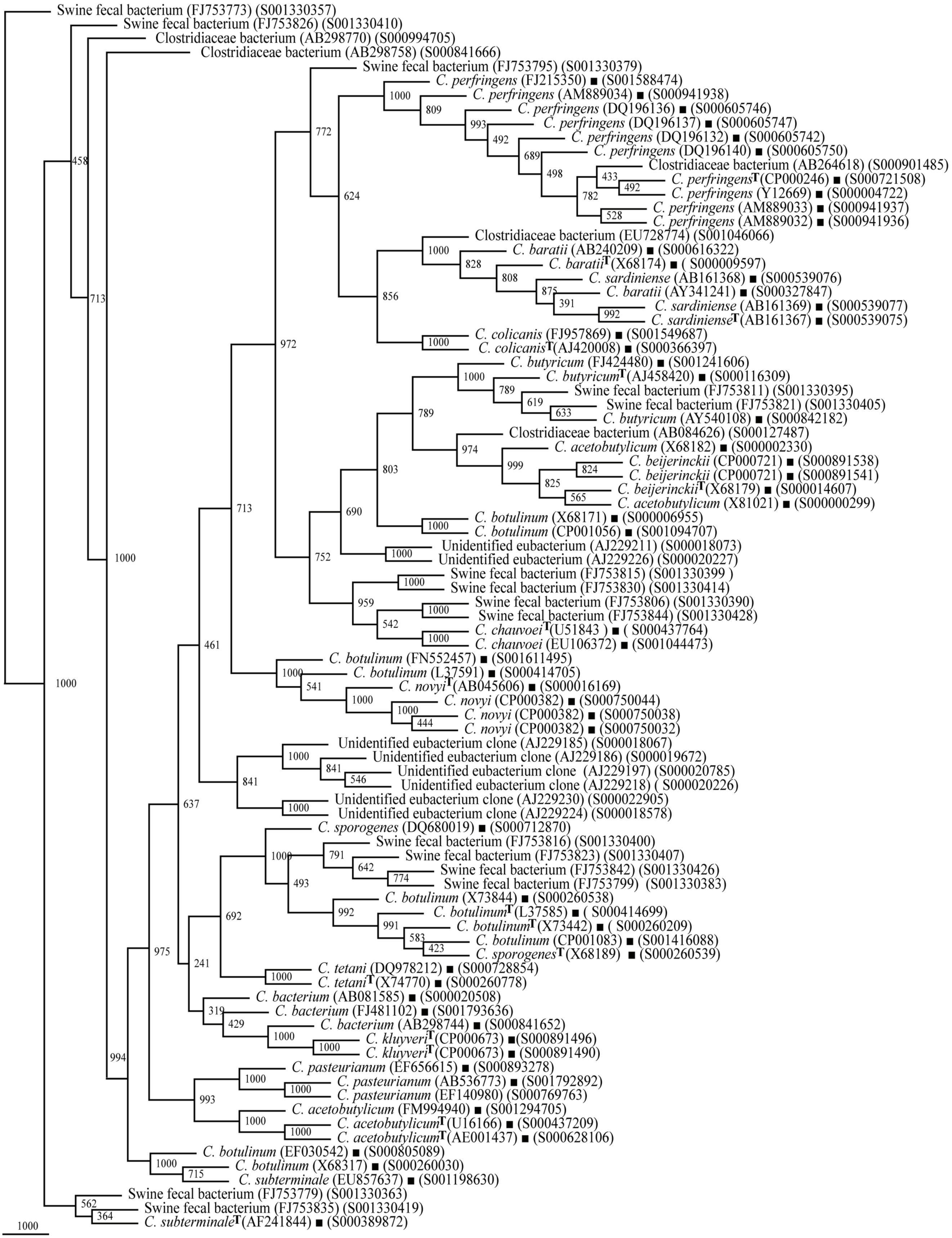

Figure S16 Phylogenetic tree of 16S rDNA of *Clostridium* spp. and Framework sequences. A neighbor – joining analysis with Jukes–Cantor correction and bootstrap support was performed on the 16S rDNA sequences of Swine fecal bacterium, Clostridiaceae bacterium and unidentified eubacterium – 31 along with 56 of phylogenetic framework (Figure 1). Bootstrap values are given at nodes. Sequences marked by filled square are the ones considered as framework in the study whereas type strains are indicated by 'T' as superscript. Values in parentheses are accession numbers (RDP and NCBI) (http://rdp.cme.msu.edu/ and http://www.ncbi.nlm.nih.gov/).
