# Supplementary material for: Analysis of the unexplored features of rrs (16S rDNA) of the Genus Clostridium
Source: BMC Genomics. 2011 Jan 11;12:18. doi: 10.1186/1471-2164-12-18 (PMC3024285; doi:10.1186/1471-2164-12-18)
Supplement: Additional file 4 — Tables S2-S3 'Novel' and low frequency Clostridium spp. File contains representative rrs sequences of unsegregated Clostridium sp. (novel) and Clostridium spp. with small population sizes. [file 1471-2164-12-18-S4.DOC]

| **Table S2: Representative 16S rDNA sequences of unsegregated *Clostridium* sp.^a^** | | |
| --- | --- | --- |
| **S.**  **No.** | **Unsegregated *Clostridium* sp.** | |
|  | **Member(s) of a clade** | **Representative sequences** |
| 1a | S000334965, S000334966, S000334967 | S000334966 |
| 1b | S000334963 | S000334963 |
| 1c | S000334969 | S000334969 |
| 1d | S000334970 | S000334970 |
| 1e | S000334964, S000334968, S000334973, S000334972, S001199652 | S001199652, S000334964 |
| 2 | S001152393, S000514472 | S000514472 |
| 3a | S001244381 | S001244381 |
| 3b | S001244385 | S001244385 |
| 3c | S001244377 | S001244377 |
| 3d | S001244382, S001244387, S001244378 | S001244378 |
| 3e | S001244375 | S001244375 |
| 3f | S001244384 | S001244384 |
| 3g | S001244373 | S001244373 |
| 3h | S001244371 | S001244371 |
| 3i | S001244380 | S001244380 |
| 3j | S001244379, S001244370, S001244372 | S001244372 |
| 4a | S000005475 | S000005475 |
| 4b | S000994781, S000386951 | S000386951 |
| 4c | S000334974 | S000334974 |
| 4d | S000334984 | S000334984 |
| 4e | S000334986 | S000334986 |
| 4f | S000334989 | S000334989 |
| 4g | S000009609 | S000009609 |
| 4h | S000335312 | S000335312 |
| 4i | S000400857 | S000400857 |
| 4j | S000400858 | S000400858 |
| 4k | S000400859 | S000400859 |
| 4l | S000334976, S000334977 | S000334977 |
| 4m | S000334978 | S000334978 |
| 4n | S000334983 | S000334983 |
| 4o | S000334982 | S000334982 |
| 4p | S000334980 | S000334980 |
| 4q | S000334979, S000334981 | S000334981 |
| 4r | S000335470, S000335471 | S000335471 |
| 4s | S000335494 | S000335494 |
| 4t | S000335486 | S000335486 |
| 4u | S000335487 | S000335487 |
| 5 | S000139890 | S000139890 |
| 6a | S001241607, S001199658, S001199664, S001199354, S001293399 | S001241607, S001293399 |
| 6b | S001241598 | S001241598 |
| 6c | S001199657 | S001199657 |
| 6d | S001199647 | S001199647 |
| 6e | S001418739 | S001418739 |
| 6f | S001352517 | S001352517 |
| 7 | S000400865, S001572695 | S000400865 |
| 8a | S001241600 | S001241600 |
| 8b | S000690904, S001152395, S000776523,  S000335504 | S000335504, S000690904 |
| 9 | S001415997, S001415998, S001415999, S001416000, S001199660, S001199661 | S001199660, S001415997 |
| 10 | S001170673 | S001170673 |
| 11 | S001548586, S000485869 | S000485869 |
| 12 | S000330874, S000330869, S001264257, S000722658, S000022010 | S000330874, S000022010 |
| Total | 84 sequences | 56 sequences |
| ^a^Phylogenetic tree drawn on the basis of all the sequences of unsegregated *Clostridium* sp. has been presented in Figure S17. Selected representative sequences of Clusters 1 to 4k were used in Figure S18 whereas rest (4l to 12) were used in Figure S19. | | |

| **Table S3** Representative 16S rDNA sequences of *Clostridium* spp. with small population sizes.^a^ | | | |
| --- | --- | --- | --- |
| **S. No.** | **Species Name** | **Species Member** | **Used as Representative** |
| 1 | *Clostridium paraputrificum* | S000128907 (T), S000260334, S001792890, S000529507, S000253648 | S000128907 (T), S000260334 |
| 2 | *C. diolis* | S000115736 (T), S000116547, S000116809, 000722359 | S000115736 (T) |
| 3 | *C. saccharobutylicum* | S000437206 (T), S001744855, S001094446, | S000437206 (T) |
| 4 | *C. thiosulfatireducens* | S000434520 (T), S000804481, S000391440, S001169692 | S000434520 (T) |
| 5 | *C. tyrobutyricum* | S000436476 (T), S001795516, S001795519, S000414327 | S000436476 (T), S001795516 |
| 6 | *C. bowmanii* | S000137817 (T), S000022497, S000136990 | S000137817 (T) |
| 7 | *C. estertheticum* | S000380961 (T), S000137876 (T), S000013400 | S000137876 (T) |
| 8 | *C. intestinale* | S000260369 (T), S000505912, S000650670 | S000260369 (T) |
| 9 | *C. ljungdahlii* | S001746376, S001746374, S001746375 | S001746376 |
| 10 | *C. neonatale* | S001155546, S001155556, S00390501 | S001155546, S001155556 |
| 11 | *C. septicum* | S000437948 (T), S000859058, S001241604 | S000437948 (T) |
| 12 | *C. tetanomorphum* | S000620000 (T), S000380964, S000004728 | S000620000 (T) |
| 13 | *C. algidicarnis* | S000387304 (T), S000260168 | S000387304 (T) |
| 14 | *C. aurantibutyricum* | S000001564 (T), S001199386 | S000001564 (T) |
| 15 | *C. cellulovorans* | S000005084 (T), S000260453 | S000005084 (T) |
| 16 | *C. cochlearium* | S000436458 (T), S001792987 | S000436458 (T) |
| 17 | *C. disporicum* | S000015682 (T), S000722723 | S000015682 (T) |
| 18 | *C. drakei* | S000005089 (T), S000129659 | S000005089 (T) |
| 19 | *C. fallax* | S000436453 (T), S000325460 | S000436453 (T) |
| 20 | *C. felsineum* | S000390415, S000390414 | S000390415 |
| 21 | *C. frigoris* | S000138148 (T), S000138430 | S000138148 (T) |
| 22 | *C. gasigenes* | S000428722 (T), S000388102 | S000428722 (T) |
| 23 | *C. haemolyticum* | S000004827 (T), S000009602 | S000004827 (T) |
| 24 | *C. limosum* | S000436461, S000927438 | S000436461 |
| 25 | *C. puniceum* | S000021696 (T), S000259939 | S000021696 (T) |
| 26 | *C. putrefaciens* | S000387305 (T), S000010368 | S000387305 (T) |
| 27 | *C. roseum* | S000022312 (T), S000010371 | S000022312 (T) |
| 28 | *C. sartagoforme* | S000004297 (T), S001199654 | S000004297 (T) |
| 29 | *C. tertium* | S000000413 (T), S000011940 | S000000413 (T) |
| 30 | *C. thermopalmarium* | S000008489 (T), S000007073 | S000008489 (T) |
| 31 | *C. longisporum* | S000260203 | S000260203 |
| 32 | *C. lundense* | S000576711 (T) | S000576711 (T) |
| 33 | *C. tunisiense* | S000400761 | S000400761 |
| 34 | *C. acetireducens* | S000004716 (T) | S000004716 (T) |
| 35 | *C. acidisoli* | S000130726 (T) | S000130726 (T) |
| 36 | *C. aciditolerans* | S000701420 (T) | S000701420 (T) |
| 37 | *C. aestuarii* | S000585968 (T) | S000585968 (T) |
| 38 | *C. akagii* | S000127491 (T) | S000127491 (T) |
| 39 | *C. algoriphilum* | S000396287 | S000396287 |
| 40 | *C. argentinense* | S000260680 (T) | S000260680 (T) |
| 41 | *C. autoethanogenum* | S000014226 | S000014226 |
| 42 | *C. bovipellis* | S001094884 | S001094884 |
| 43 | *C. caenicola* | S000943595 (T) | S000943595 (T) |
| 44 | *C. caliptrosporum* | S000260032 | S000260032 |
| 45 | *C. carnis* | S000436456 (T) | S000436456 (T) |
| 46 | *C. celatum* | S000260781 (T) | S000260781 (T) |
| 47 | *C. chartatabidum* | S000009214 (T) | S000009214 (T) |
| 48 | *C. chromoreductans* | S000403224 | S000403224 |
| 49 | *C. collagenovorans* | S000260454 (T) | S000260454 (T) |
| 50 | *C. corinoforum* | S000260488 | S000260488 |
| 51 | *C. crotonatovorans* | S000426077 | S000426077 |
| 52 | *C. cylindrosporum* | S000022469 (T) | S000022469 (T) |
| 53 | *C. favososporum* | S000260731 | S000260731 |
| 54 | *C. frigidicarnis* | S000428393 (T) | S000428393 (T) |
| 55 | *C. frigoriphilum* | S000860540 | S000860540 |
| 56 | *C. ganghwense* | S000482460 | S000482460 |
| 57 | *C. grantii* | S000016392 (T) | S000016392 (T) |
| 58 | *C. histolyticum* | S000436459 | S000436459 |
| 59 | *C. homopropionicum* | S000260249 (T) | S000260249 (T) |
| 60 | *C. lacusfryxellense* | S000138431 (T) | S000138431 (T) |
| 61 | *C. magnum* | S001746047 | S001746047 |
| 62 | *C. mesophilum* | S000903067 (T) | S000903067 (T) |
| 63 | *C. nitrophenolicum* | S000690535 (T) | S000690535 (T) |
| 64 | *C. pascui* | S000003885 (T) | S000003885 (T) |
| 65 | *C. peptidivorans* | S000388299 (T) | S000388299 (T) |
| 66 | *C. proteolyticum* | S000260571 (T) | S000260571 (T) |
| 67 | *C. proteolyticus* | S000539419 (T) | S000539419 (T) |
| 68 | *C. psychrophilum* | S000002328 (T) | S000002328 (T) |
| 69 | *C. quinii* | S000260250 (T) | S000260250 (T) |
| 70 | *C. ragsdalei* | S000541643 | S000541643 |
| 71 | *C. saccharoperbutylacetonicum* | S000437203 (T) | S000437203 (T) |
| 72 | *C. scatologenes* | S000641401 | S000641401 |
| 73 | *C. schirmacherense* | S000608913 (T) | S000608913 (T) |
| 74 | *C. sulfidigenes* | S000805548 (T) | S000805548 (T) |
| 75 | *C. taeniosporum* | S001169181 | S001169181 |
| 76 | *C. tagluense* | S000625977 (T) | S000625977 (T) |
| 77 | *C. tepidiprofundum* | S000805416 (T) | S000805416 (T) |
| 78 | *C. thermobutyricum* | S000007751 (T) | S000007751 (T) |
| 79 | *C. uliginosum* | S000131270 (T) | S000131270 (T) |
| 80 | *C. vincentii* | S000014235 (T) | S000014235 (T) |
| ^a^In most of the cases, type strains were used as representative(s), totaling 83 in number. | | | |
