# Supplementary material for: Analysis of the unexplored features of rrs (16S rDNA) of the Genus Clostridium
Source: BMC Genomics. 2011 Jan 11;12:18. doi: 10.1186/1471-2164-12-18 (PMC3024285; doi:10.1186/1471-2164-12-18)
Supplement: Additional file 5 — Figure S17 Phylogenetic tree of 84 16S rDNA sequences of 'novel' Clostridium species. File contains a neighbor - joining analysis of 56 representative novel Clostridium sequences. [file 1471-2164-12-18-S5.PDF]

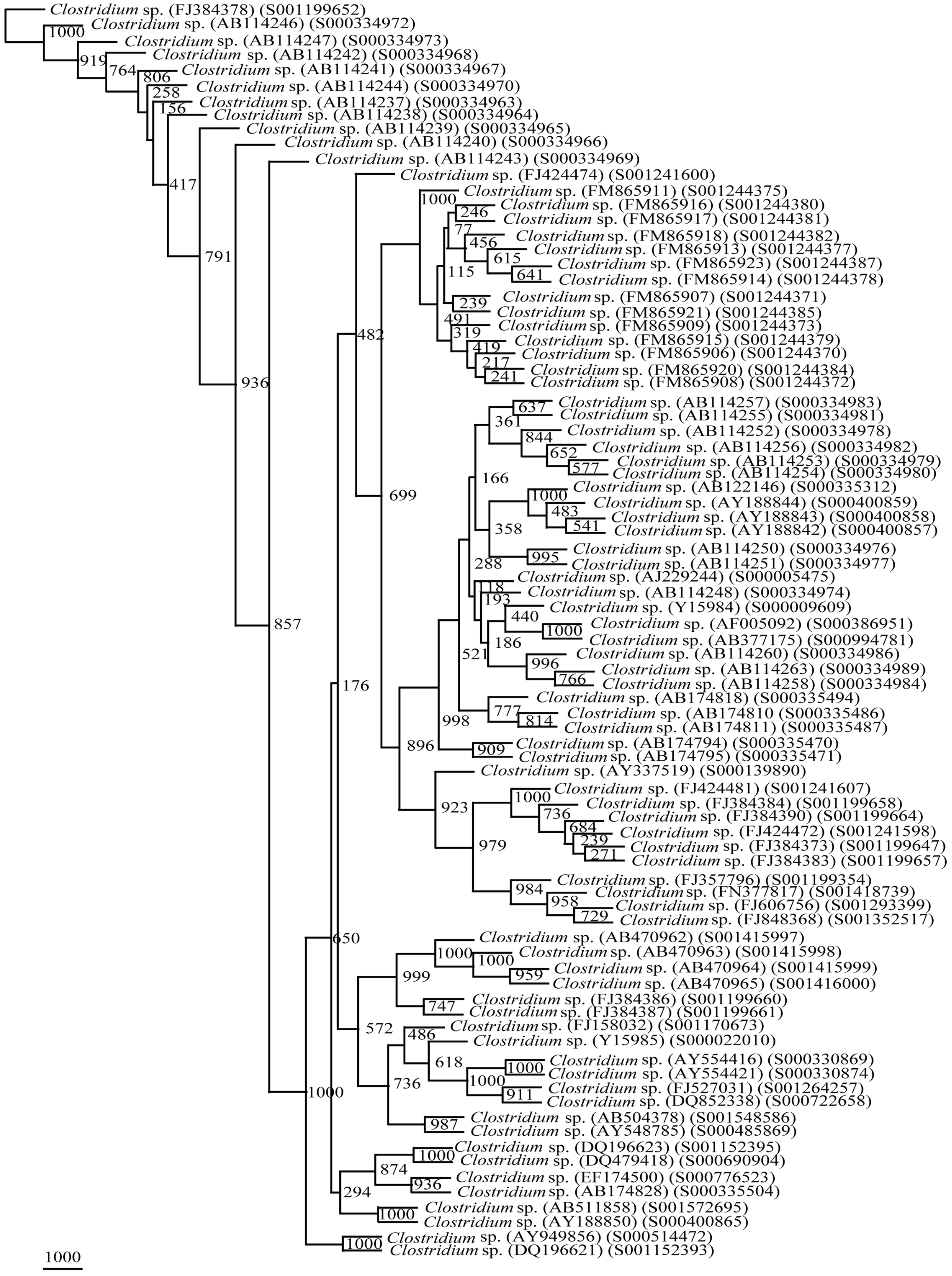

Figure S17 Phylogenetic tree of 84 16S rDNA sequences of 'novel' *Clostridium* species. A neighbor – joining analysis with Jukes–Cantor correction and bootstrap support was performed on the gene sequences. Bootstrap values are given at nodes. Sequences marked by filled square are the ones considered as representative (56) of the 12 clusters. Values in parentheses are accession numbers (RDP and NCBI) (http://rdp.cme.msu.edu/ and http://www.ncbi.nlm.nih.gov/).
