# Supplementary material for: Analysis of the unexplored features of rrs (16S rDNA) of the Genus Clostridium
Source: BMC Genomics. 2011 Jan 11;12:18. doi: 10.1186/1471-2164-12-18 (PMC3024285; doi:10.1186/1471-2164-12-18)
Supplement: Additional file 6 — Figures S18-S19 Phylogenetic tree of 16S rDNA of novel Clostridium spp. and low frequency Clostridium spp. File contains a neighbor - joining analysis performed on the rrs sequences of novel Clostridium sp. (Additional file 4: Table S2) 56 along with 83 representatives of Clostridium sp. known to occur at low frequency (Additional file 4: Table S3). [file 1471-2164-12-18-S6.PDF]

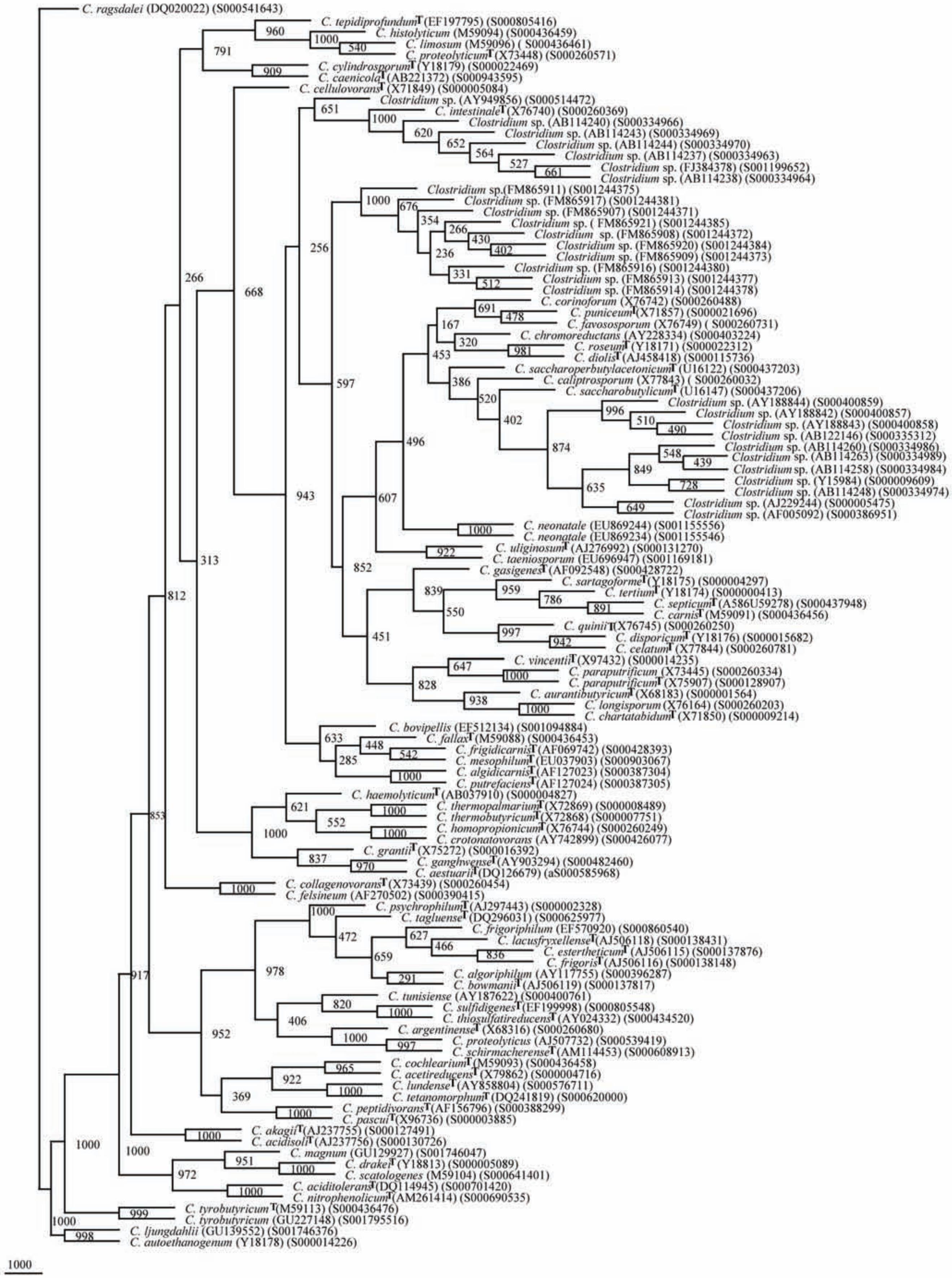

Figure S18 Phylogenetic tree of 16S rDNA of novel *Clostridium* spp. (1-28) and low frequency *Clostridium* spp. A neighbor – joining analysis with Jukes–Cantor correction and bootstrap support was performed on the 16S rDNA sequences of novel *Clostridium* sp. (Additional file 4: Table S2, Clusters 1 to 4k, 1-28) - 28 along with 83 representatives of *Clostridium* sp. known to occur at low frequency (Additional file 4: Table S3). Bootstrap values are given at nodes. Sequences marked by filled square are the ones considered as framework in the study whereas type strains are indicated by 'T' as superscript. Values in parentheses are accession numbers (RDP and NCBI) (http://rdp.cme.msu.edu/ and http://www.ncbi.nlm.nih.gov/. Note: Out of a total of 56 *Clostridium* spp. 16S rDNA sequences (Additional file 5: Figure S17), 28 have been presented here to achieve clarity of presentation. The rest 28 16S rDNA sequences have been presented in Additional file 6: Figure S19.

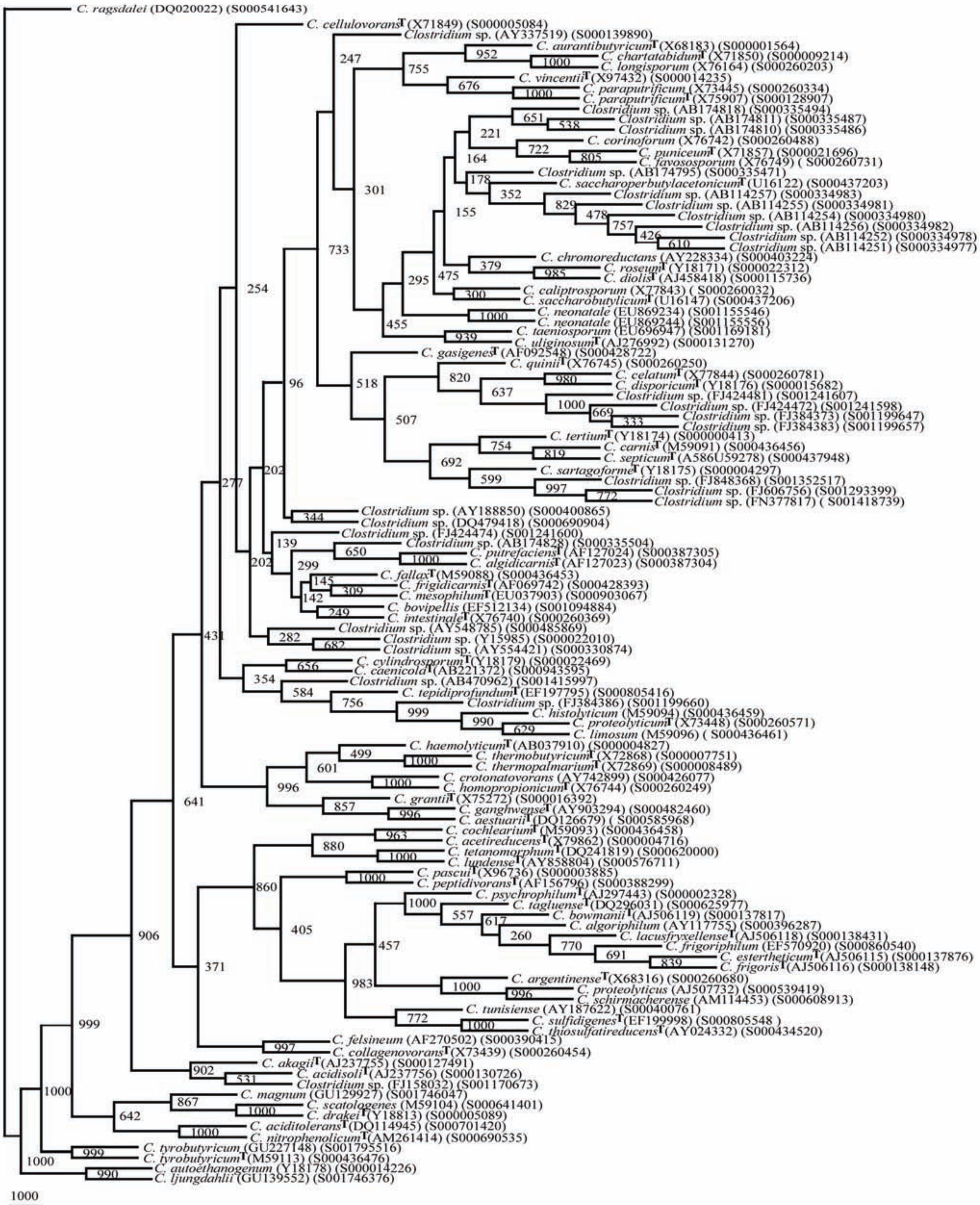

Figure S19. Phylogenetic tree of 16S rDNA of novel *Clostridium* spp. (29-56) and low frequency *Clostridium* spp. A neighbor – joining analysis with Jukes–Cantor correction and bootstrap support was performed on the 16S rDNA sequences of novel *Clostridium* sp. (Additional file 4: Table S2, Clusters 4l to 12, 29-56) - 28 along with 83 representatives of *Clostridium* sp. known to occur at low frequency (Additional file 4: Table S3). Bootstrap values are given at nodes. Sequences marked by filled square are the ones considered as framework in the study whereas type strains are indicated by 'T' as superscript. Values in parentheses are accession numbers (RDP and NCBI) (http://rdp.cme.msu.edu/ and http://www.ncbi.nlm.nih.gov/). Note: Out of a total of 56 *Clostridium* spp. 16S rDNA sequences (Additional file 5: Figure S17), 28 have been presented here to achieve clarity of presentation. The first 28 16S rDNA sequences have been presented in Additional file 6: Figure S18.
