# Supplementary material for: Analysis of the unexplored features of rrs (16S rDNA) of the Genus Clostridium
Source: BMC Genomics. 2011 Jan 11;12:18. doi: 10.1186/1471-2164-12-18 (PMC3024285; doi:10.1186/1471-2164-12-18)
Supplement: Additional file 7 — Table S4 RE sites with low frequency. File contains low frequency in silico Restriction Enzymes cut sites in rrs sequences of different Clostridium spp. [file 1471-2164-12-18-S7.DOC]

| **Table S4: Low frequency *in silico* Restriction Enzyme cut sites in 16S rDNA sequences of different *Clostridium* spp.** | | | | | | | | |
| --- | --- | --- | --- | --- | --- | --- | --- | --- |
| **Organism** | **F ^a^** | **BamHI** | **NotI** | **PstI** | **SacI** | **HindIII** | **EcoRI** | **NruI** |
| *C. acetobutylicum* | 24 | 0 | 0 | 0 | 0 | 0 | 24(1) | 24(1) |
| *C. baratii* | 8 | 0 | 0 | 0 | 0 | 0 | 8(1) | 8(1) |
| *C. beijerinckii* | 23 | 0 | 0 | 0 | 0 | 2(1) | 23(1) | 23(1) |
| *C. botulinum* | 128 | 0 | 0 | 0 | 0 | 88(1) | 128(1) | 126(1) |
| *C. butyricum* | 32 | 0 | 0 | 0 | 0 | 1(1) | 32(1) | 30(2)+2(1) |
| *C. colicanis* | 9 | 0 | 0 | 0 | 0 | 0 | 9(1) | 9(1) |
| *C. chauvoei* | 8 | 0 | 0 | 0 | 0 | 0 | 8(1) | 8(1) |
| *C. kluyveri* | 14 | 0 | 0 | 0 | 0 | 0 | 14(1) | 14(1) |
| *C. novyi* | 17 | 0 | 0 | 0 | 0 | 15(1) | 17(1) | 17(1) |
| *C. pasteurianum* | 13 | 0 | 0 | 0 | 0 | 2(1) | 13(1) | 1(1) |
| *C. perfringens* | 92 | 0 | 0 | 2(1) | 0 | 0 | 92(1) | 91(1) |
| *C. sardiniense* | 9 | 0 | 0 | 0 | 0 | 0 | 9(1) | 9(1) |
| *C. sporogenes* | 11 | 0 | 0 | 0 | 0 | 8(1) | 11(1) | 10(1) |
| *C. subterminale* | 8 | 0 | 0 | 0 | 4(1) | 1(1) | 8(1) | 8(1) |
| *C. tetani* | 8 | 0 | 0 | 0 | 0 | 8(2) | 8(1) | 0 |
| Numbers in parentheses denote number of cut sites in 16S rDNA sequences. | | | | | | | | |
| ^a^ No. of organisms within the corresponding Clostridium sp. | | | | | | | | |
