# Supplementary material for: Analysis of the unexplored features of rrs (16S rDNA) of the Genus Clostridium
Source: BMC Genomics. 2011 Jan 11;12:18. doi: 10.1186/1471-2164-12-18 (PMC3024285; doi:10.1186/1471-2164-12-18)
Supplement: Additional file 8 — Figures S20-S36 Regular expression diagram. File contains regular expression diagram of signatures (nucleotides) of rrs sequences of 15 Clostridium spp. obtained through MEME suite. [file 1471-2164-12-18-S8.PDF]

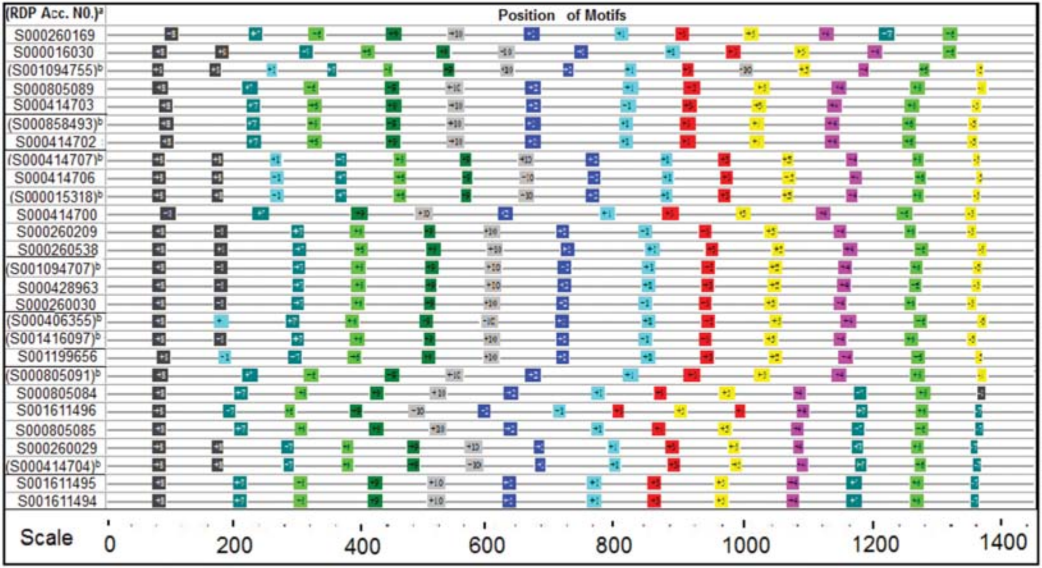

Motifs:

M1: TCGCAAGATTAAAACTCAAAGGAATTGACG
M2: AATTCCTAGTGTAGCGGTGAAATGCGTAGA
M3: GTTTAATTCGAAGCAACGCGAAGAACCTTA
M4: ATGACGTCAAATCATCATGCCCCTTATGTC
M5: ATGGTTGTCGTCAGCTCGTGTCGTGAGATG
M6: CCTACATGAAGCTGGAGTTGCTAGTAATCG
M7: ACGATGCGTAGCCGACCTGAGAGGGTGATC
M8: ATTAGCGGCGGACGGGTGAGTAACACGTGG
M9: AACTACGTGCCAGCAGCCGCGGTAATACGT
M10: AGCGTTGTCCGGATTTACTGGGCGTAAAGG

Figure S20 Regular expression diagram of signatures (nucleotides) of 128 16S rDNA sequences of *Clostridium botulinum.* (http://meme.sdsc.edu/meme4_3_0/cgi-bin/meme.cgi) [a]http://rdp.cme.msu.edu/, [b]16S rDNA sequences with similar regular expression diagram. The details of other members in each group have been presented in Additional file 11: Table S23.

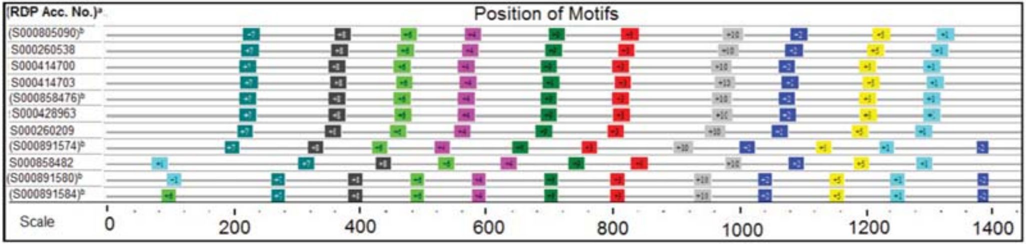

Motifs:

M1: AAAACTTATAAAACCTATCTCAGTTCGGAT
M2: AACCCTTGTTATTAGTTGCTACCATTAAGT
M3: GGTCGCAAGATTAAAACTCAAAGGAATTGA
M4: AAATGCGTAGAGATTAGGAAGAACACCAGT
M5: ACACACGTGCTACAATGGTAGGTACAATAA
M6: AAACTGGATATCTAGAGTGCAGGAGAGGAA
M7: AAGGTCTTCGGATTGTAAAGCCCTGTTTTC
M8: GTAGGCGGATGTTTAAGTGGGATGTGAAAT
M9: TAAACGATGGATACTAGGTGTAGGGGGTAT
M10: TGTCGTGAGATGTTAGGTTAAGTCCTGCAA

Figure S21 Regular expression diagram of signatures (nucleotides) of 83 16S rDNA sequences of *Clostridium botulinum.* [a]http://rdp.cme.msu.edu/ , [b]16S rDNA sequences with similar regular expression diagram. The details of other members in each group have been presented in Additional file: Table S24.

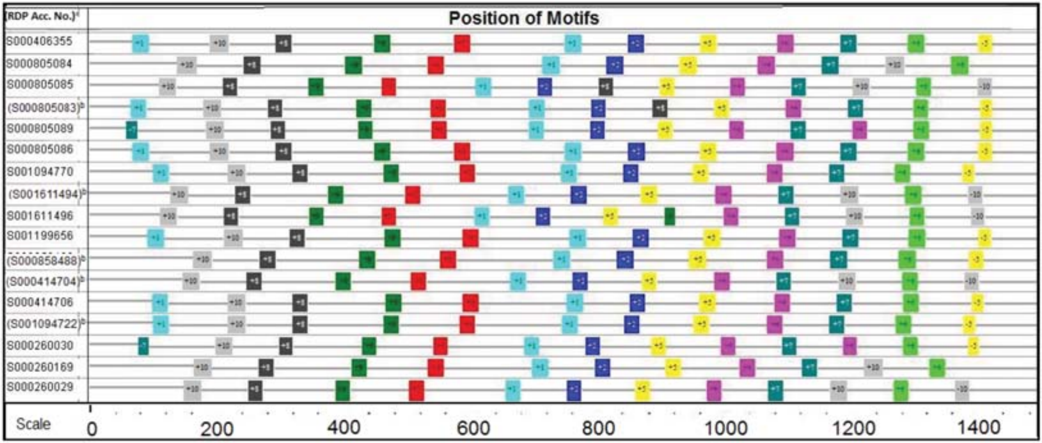

Motifs:

M1: TCGCAAGATTAAAACTCAAAGGAATTGACG
M2: ATGTGGTTTAATTCGAAGCAACGCGAAGAA
M3: AATTCCTAGTGTAGCGGTGAAATGCGTAGA
M4: AGGAAGGTGGGGATGACGTCAAATCATCAT
M5: ATGGTTGTCGTCAGCTCGTGTCGTGAGATG
M6: GATTGTAGGCTGAAACTCGCCTACATGAAG
M7: TTATGTCTAGGGCTACACACGTGCTACAAT
M8: ACTCCTACGGGAGGCAGCAGTGGGGAATAT
M9: AAGCGTTGTCCGGATTTACTGGGCGTAAAG
M10: ACGATGCGTAGCCGACCTGAGAGGGTGATC

Figure S22 Regular expression diagram of signatures (nucleotides) of 45 16S rDNA sequences of *Clostridium botulinum.* [a]http://rdp.cme.msu.edu/ , [b]16S rDNA sequences with similar regular expression diagram. The details of other members in each group have been presented in Additional file 11: Table S25.

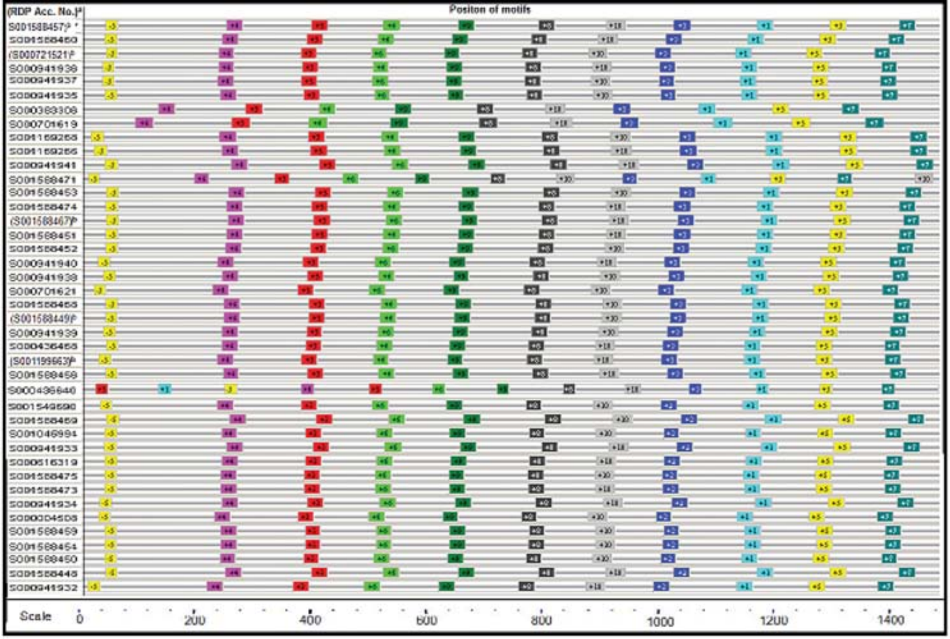

Motifs:
M1: TTAGTTACTACCATTAAGTTGAGGACTCTA
M2: ACACTTGACATCCCTTGCATTACTCTTAAT
M3: TAGGCGGATGATTAAGTGGGATGTGAAATA
M4: AAGGTTTTCGGATCGTAAAGCTCTGTCTTT
M5: TTATGTGTAGGGCTACACACGTGCTACAAT
M6: AGAGTGGAATTCCTAGTGTAGCGGTGAAAT
M7: AGAGTGGAATTCCTAGTGTAGCGGTGAAAT
M8: AGTACGGTCGCAAGATTAAAACTCAAAGGA
M9: AAAGCGTGGGGAGCAAACAGGATTAGATAC
M10: TAATTCGAAGCAACGCGAAGAACCTTACCT

Figure S23 Regular expression diagram of signatures (nucleotides) of 92 16S rDNA sequences of *Clostridium perfringens*. [a]http://rdp.cme.msu.edu/, [b]16S rDNA sequences with similar regular expression diagram. The details of other members in each group have been presented in Additional file 11: Table S26.

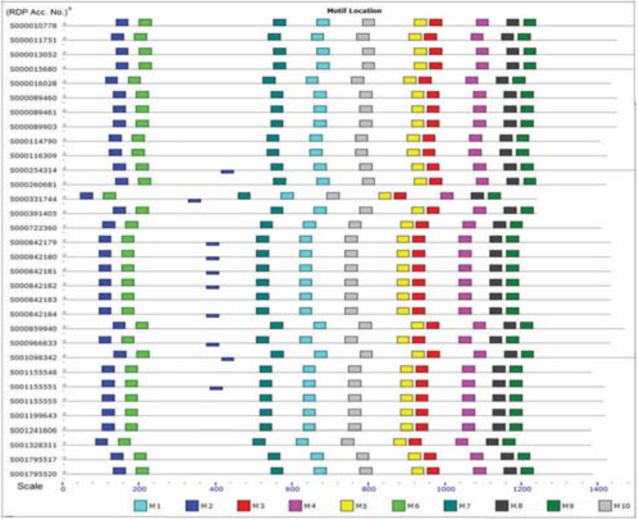

Motifs:

M1: AAATGCGTAGAGATTAGGAAGAATACCAGT
M2: AATAGCCTTTCGAAAGGAAGATTAATACCG
M3: ACTTGACATCTCCTGAATTACTCTGTAATG
M4: AACCCTTATTGTTAGTTGCTACCATTTAGT
M5: ATGTGGTTTAATTCGAAGCAACGCGAAGAA
M6: ATTAAAGGAGTAATCCGCTATGAGATGGAC
M7: GTAGGTGGATATTTAAGTGGGATGTGAAAT
M8: ACGTCAAATCATCATGCCCCTTATGTCTAG
M9: TACAATGGTCGGTACAATGAGATGCAACCT
M10: TAAACGATGAATACTAGGTGTAGGGGTTGT

Figure S24 Regular expression diagram of signatures (nucleotides) of 32 16S rDNA sequences of *Clostridium butyricum*. [a]http://rdp.cme.msu.edu/.

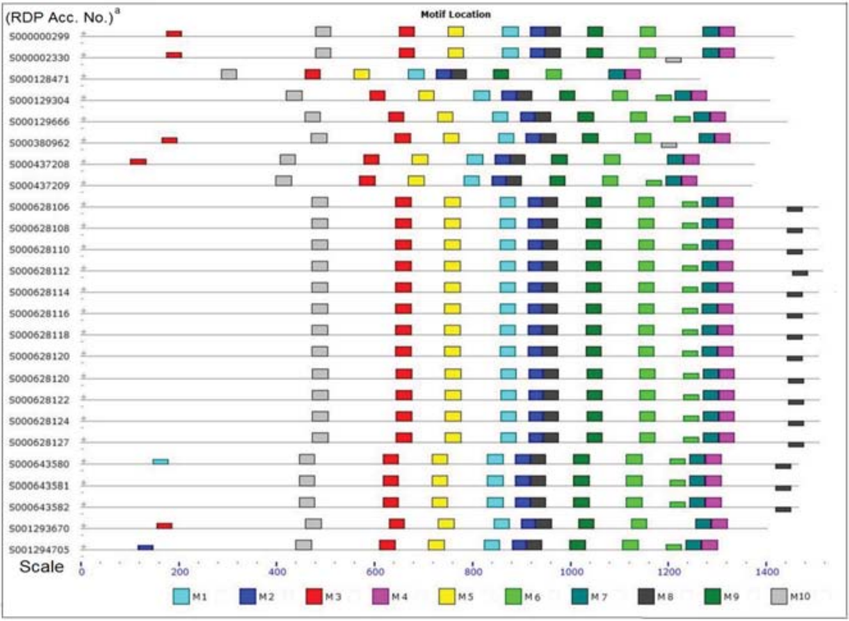

Motifs:

M1: ACGGTCGCAAGATTAAAACTCAAAGGAATT
M2: ATGTGGTTTAATTCGAAGCAACGCGAAGAA
M3: CTAGTGTAGCGGTGAAATGCGTAGAGATTA
M4: AGTTGCTAGTAATCGCGAATCAGAATGTCG
M5: AAACAGGATTAGATACCCTGGTAGTCCACG
M6: AGGAAGGTGGGGATGACGTCAAATCATCAT
M7: ATTGTAGGCTGAAACTCGCCTACATGAAGC
M8: CCTTACCTAGACTTGACATCTCCTGAATTA
M9: AGCTCGTGTCGTGAGATGTTGGGTTAAGTC
M10: AACTACGTGCCAGCAGCCGCGGTAATACGT

Figure S25 Regular expression diagram of signatures (nucleotides) of 24 16S rDNA of sequences *Clostridium acetobutylicum*. [a]http://rdp.cme.msu.edu/.

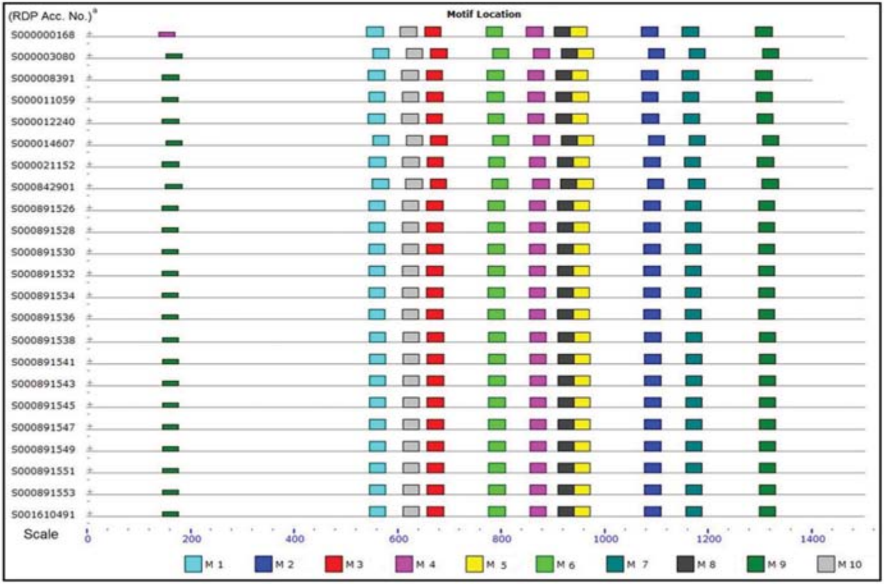

Motifs:
M1: TATTGTTAGTTGCTACCATTTAGTTGAGCA
M2: TAGGTGGATATTTAAGTGGGATGTGAAATA
M3: AAATGCGTAGAGATTAGGAAGAATACCAGT
M4: ACGGTCGCAAGATTAAAACTCAAAGGAATT
M5: AACCTTACCTAGACTTGACATCTCCTGAAT
M6: TAAACGATGAATACTAGGTGTAGGGGTTGT
M7: AAATCATCATGCCCCTTATGTCTAGGGCTA
M8: AGCATGTGGTTTAATTCGAAGCAACGCGAA
M9: AGTTGCTAGTAATCGCGAATCAGAATGTCG
M10: ATATCTAGAGTGCAGGAGAGCAAAGTAGAA

Figure S26 Regular expression diagram of signatures (nucleotides) of 23 16S rDNA of sequences *Clostridium beijerinckii.* [a]http://rdp.cme.msu.edu/.

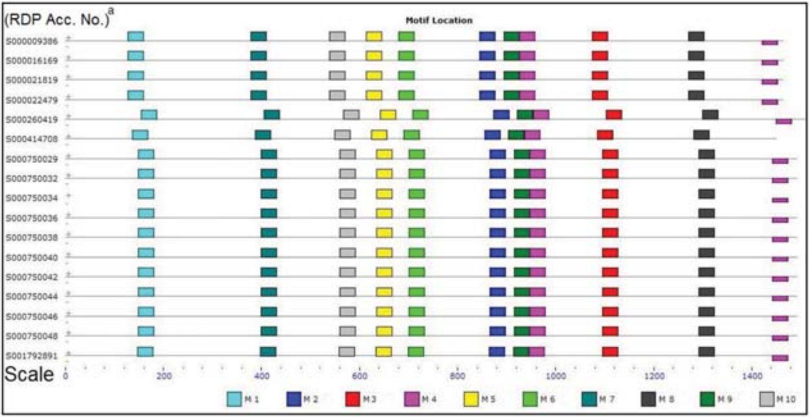

Motifs:

M1: AAAGGGAGATTAATACCGCATAACATTATT
M2: AAGATTAAAACTCAAAGGAATTGACGGGGA
M3: ACTATTAAGTTAAGCACTCTAACGAGACTG
M4: TTACCTAGACTTGACATCTCCTGAATTACT
M5: AAAGTGGAATTCCTAGTGTAGCGGTGAAAT
M6: ACTTTCTGGACTGTAACTGACACTGAGATA
M7: AGTGATGAAGGTTTTCGGATCGTAAAACTC
M8: ATGAAGTTGGAGTTGCTAGTAATCGCGAAT
M9: ATGTGGTTTAATTCGAAGCAACGCGAAGAA
M10: TTAAGTCAGATGTGAAATTCCCGGGCTTAA

Figure S27 Regular expression diagram of signatures (nucleotides) of 17 16S rDNA sequences of *Clostridium novyi.* [a]http://rdp.cme.msu.edu/.

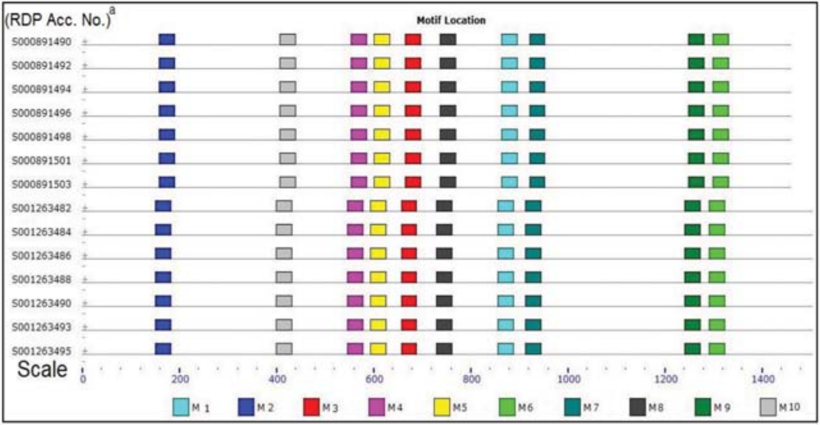

Motifs:

M1: ACGATCGCAAGATTAAAACTCAAAGGAATT
M2: AGATTAATACCGCATAGAAGGTAAAAATCG
M3: AAATGCGTAGAGATTAGGAAGAACACCAGT
M4: AGGCGGATATTTAAGTGAGATGTGAAAGAC
M5: AGTGCATTTCAAACTGGATATCTAGAGTGC
M6: ATGAAGTTGGAGTTGCTAGTAATCGCGAAT
M7: ATGTGGTTTAATTCGAAGCAACGCGAAGAA
M8: AAAGCGTGGGTAGCAAACAGGATTAGATAC
M9: AAATCTCAAAAACTGCCCCCAGTTCGGATT
M10: AAGAAGGTTTTCGGATCGTAAAGCTCTGTC

Figure S28 Regular expression diagram of signatures (nucleotides) of 14 16S rDNA sequences of *Clostridium kluyveri*. [a]http://rdp.cme.msu.edu/.

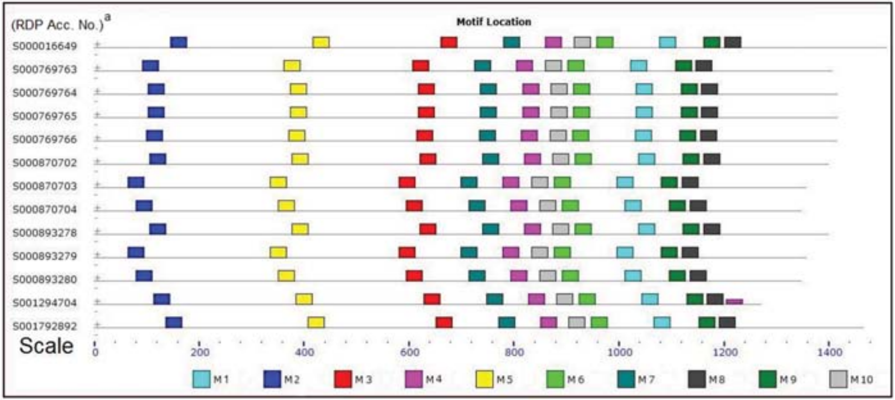

Motifs:

M1: AACCCTTATCATTAGTTGCTACCATTAAGT
M2: CGAAAGGGAGATTAATACCGCATAATATTA
M3: AAATGCGTAGAGATTAGGAAGAACATCAGT
M4: ACGGTCGCAAGATTAAAACTCAAAGGAATT
M5: ATTGTAAAGCTCTGTCTTTTGGGACGATAA
M6: ACTTGACATCTCCTGAATAGCGTAGAGATA
M7: TAAACGATGAGTACTAGGTGTAGGAGGTAT
M8: TACAATGGTGAGAACAACGAGATGCAATAC
M9: AAATCATCATGCCCCTTATGTCTAGGGCTA
M10: AGCATGTGGTTTAATTCGAAGCAACGCGAA

Figure S29 Regular expression diagram of signatures (nucleotides) of 13 16S rDNA sequences of *Clostridium pasteurianum*. [a]http://rdp.cme.msu.edu/.

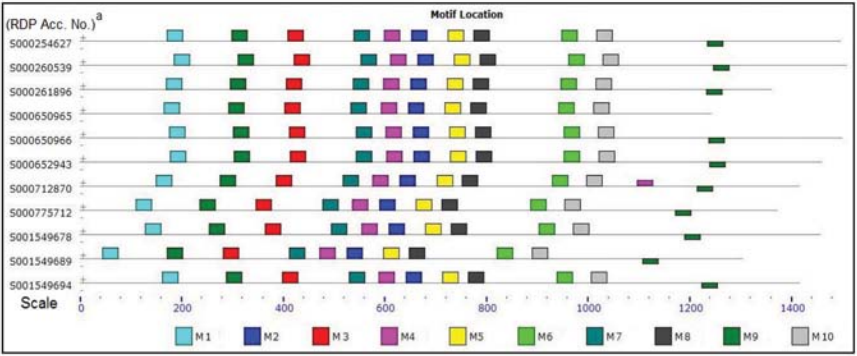

Motifs:

M1: AATCGCATGATTATCTTATCAAAGATTTAT
M2: AAATGCGTAGAGATTAGGAAGAACACCAGT
M3: ATTGTAAAGCCCTGTTTTCTGGGACGATAA
M4: AAACTGGATATCTAGAGTGCAGGAGAGGAA
M5: AAAGCGTGGGTAGCAAACAGGATTAGATAC
M6: ACTTGACATCCCTTGCATAGCCTAGAGATA
M7: GTAGGCGGATGTTTAAGTGGGATGTGAAAT
M8: ACGATGGATACTAGGTGTAGGGGGTATCAA
M9: ATTGGAACTGAGACACGGTCCAGACTCCTA
M10: GTTGTCGTCAGCTCGTGTCGTGAGATGTTA

Figure S30 Regular expression diagram of signatures (nucleotides) of 11 16S rDNA sequences of *Clostridium sporogenes*. [a]http://rdp.cme.msu.edu/.

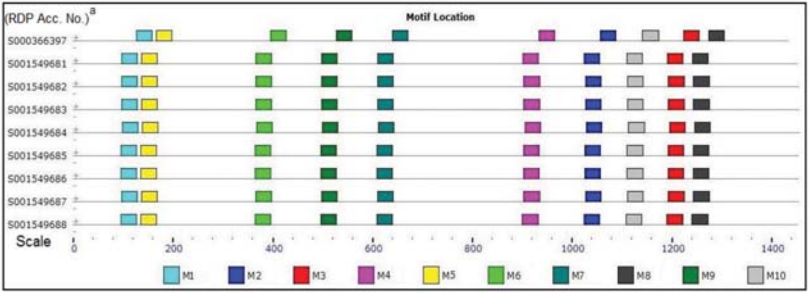

Motifs:

M1: AAAGGGAGATTAATACCTCATAATATCCTA
M2: AACCCTTATTGTTAGTTGCTACCATTTAGT
M3: AAAACTTTAAAACCGGTCTCAGTTCGGATT
M4: AGACTTGACATCTTCTGCATTACCCTTAAT
M5: ATACATGGATTAAAGGAGCAATCCGCTATA
M6: ATTGTAAAGCTCTGTCTTTAGGGACGATAA
M7: AAATGCGTAGAGATTAGGAAGAACACCAGT
M8: ATGAAGCTGGAGTTACTAGTAATCGCGAAT
M9: TAGGCGGATCTTTAAGTGGGATGTGAAATA
M10: AAATCATCATGCCCCTTATGTCTAGGGCTA

Figure S31 Regular expression diagram of signatures (nucleotides) of 9 16S rDNA sequences of *Clostridium colicanis*. [a]http://rdp.cme.msu.edu/.

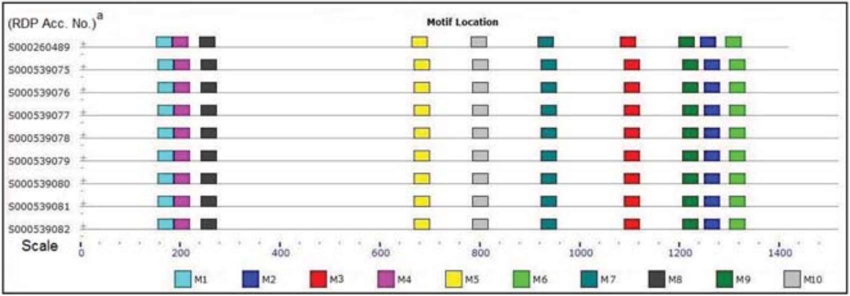

Motifs:
M1: AAAGGAAGATTAATACCGCATAACATTGCA
M2: TAAACTTCAAAACTTGTCTCAGTTCGGATT
M3: TATTGTTAGTTGCTACCATTAAGTTGAGCA
M4: TTCGCATGAAACAGCAATTAAAGGAGCAAT
M5: AAATGCGTAGAGATTAGGAAGAACACCAGT
M6: ATGAAGCTGGAGTTACTAGTAATCGCGAAT
M7: ATGTGGTTTAATTCGAAGCAACGCGAAGAA
M8: ATTAGCTAGTTGGTAAGGTAATGGCTTACC
M9: CTACAATGGCAAGTACAGAGAGATGCAATA
M10: GTAAACGATGAATACTAGGTGTAGGGGTTT

Figure S32 Regular expression diagram of signatures (nucleotides) of 9 16S rDNA sequences of *Clostridium sardiniense*. [a]http://rdp.cme.msu.edu/.

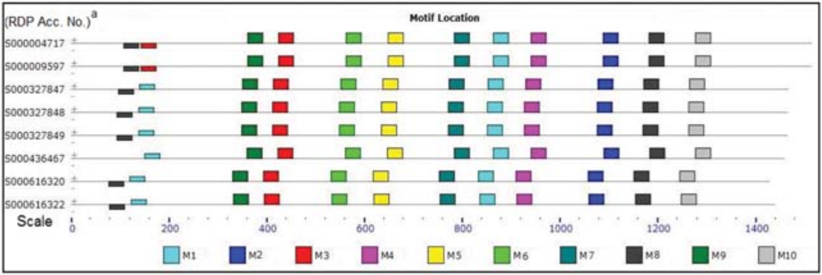

Motifs:

M1: ACGGTCGCAAGATTAAAACTCAAAGGAATT
M2: TATTGTTAGTTGCTACCATTTAGTTGAGCA
M3: TTGTAAAGCTCTGTCTTTGGGGACGATAAT
M4: AACGCGAAGAACCTTACCTAGACTTGACAT
M5: AATTCCTAGTGTAGCGGTGAAATGCGTAGA
M6: ATTTTTAAGTGGGATGTGAAATACCCGGGC
M7: CGTAAACGATGAATACTAGGTGTAGGGGTT
M8: TTATGTCTAGGGCTACACACGTGCTACAAT
M9: AATATTGCACAATGGGGGAAACCCTGATGC
M10: ATTGTAGGCTGAAACTCGCCTACATGAAGC

Figure S33 Regular expression diagram of signatures (nucleotides) of 8 16S rDNA sequences of *Clostridium baratii*. [a]http://rdp.cme.msu.edu/.

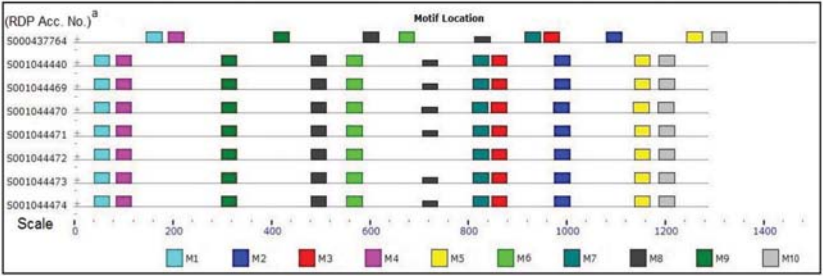

Motifs:

M1: AAAGGAAGATTAATACCGCATAATATTGCA
M2: CTTATTGTTAGTTGCTACCATTTAGTTGAG
M3: AGACTTGACATCTCCTGCATTACTCTTAAT
M4: AGTAATTAAAGGAGCAATCCGCTACAAGAT
M5: TAAACTATAATACTTGTCTCAGTTCGGATT
M6: AAATGCGTAGAGATTAGGAAGAACACCAGT
M7: ATGTGGTTTAATTCGAAGCAACGCGAAGAA
M8: AACTTGGGTGCTGCATTTCAAACTGGAAGT
M9: AAGGTTTTCGGATCGTAAAGCTCTGTCTTC
M10: ATGAAGCTGGAGTTGCTAGTAATCGCGAAT

Figure S34 Regular expression diagram of signatures (nucleotides) of 8 16S rDNA sequences of *Clostridium chauvoei*. [a]http://rdp.cme.msu.edu/.

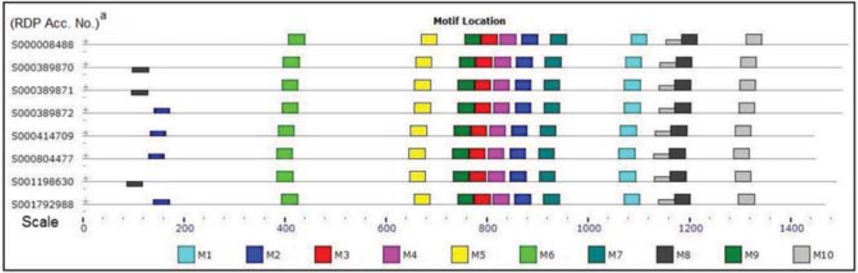

Motifs:
M1: AACCCTTATCATTAGTTGCTACCATTAAGT
M2: ACGATCGCAAGATTAAAACTCAAAGGAATT
M3: TAAACGATGAATACTAGGTGTAGGAGGTAT
M4: TTCTGTGCCGCAGTTAACACAATAAGTATT
M5: AAATGCGTAGAGATTAGGAAGAACACCAGT
M6: AATGAAGAAGGCCTTAGGGTTGTAAAGTTC
M7: ATGTGGTTTAATTCGAAGCAACGCGAAGAA
M8: TTATGTCTAGGGCTACACACGTGCTACAAT
M9: AAACAGGATTAGATACCCTGGTAGTCCACG
M10: AGTTGCTAGTAATCGCGAATCAGCATGTCG

Figure S35 Regular expression diagram of signatures (nucleotides) of 8 16S rDNA sequences of *Clostridium subterminale.* [a]http://rdp.cme.msu.edu/.

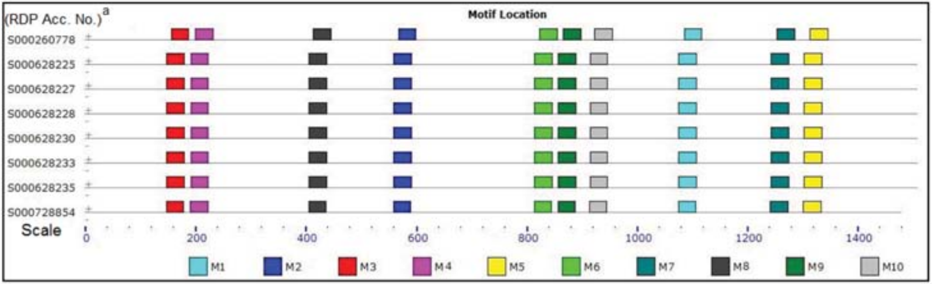

Motifs:

M1: AACCCTTATTATTAGTTGCTACCATTAAGT
M2: TTAAGTGAGATGTGAAATACCTAAGCTTAA
M3: AAAGGAGGATTAATACCGCATAAAGTTAAG
M4: TTTAACCAAAGGAGTAATCTGCTTTGAGAT
M5: AGTTGCTAGTAATCGCAAATCAGAATGTTG
M6: TTCTGTGCCGCAGTTAACACATTAAGTATT
M7: AAATCTCAAAAACCGATCCCAGTTCGGATT
M8: AAGGTTTTCGGATCGTAAAACCCTGTTTTC
M9: ACGGTCGCAAGACTAAAACTCAAAGGAATT
M10: ATGTGGTTTAATTCGAAGCAACGCGAAGAA

Figure S36 Regular expression diagram of signatures (nucleotides) of 8 16S rDNA sequences of *Clostridium tetani*. [a]http://rdp.cme.msu.edu/.
