# Supplementary material for: Analysis of the unexplored features of rrs (16S rDNA) of the Genus Clostridium
Source: BMC Genomics. 2011 Jan 11;12:18. doi: 10.1186/1471-2164-12-18 (PMC3024285; doi:10.1186/1471-2164-12-18)
Supplement: Additional file 9 — Table S5-S21 Motifs for 15 Clostridium spp. File represents motifs obtained for Clostridium botulinum (128 rrs sequences) through MEME suite and the frequency of their occurrence across other Clostridium spp. using BioEdit. [file 1471-2164-12-18-S9.DOC]

| **Table S5: Representation of motifs obtained for *Clostridium botulinum* (128 16S rDNA sequences) through stand alone MEME (version 4.3.0) software and the frequency of their occurrence across other *Clostridium* spp. using BioEdit** (<http://www.mbio.ncsu.edu/BioEdit/page2.html>). | | | | | | | | | | | |
| --- | --- | --- | --- | --- | --- | --- | --- | --- | --- | --- | --- |
| ***Clostridium* sp.** | **No. of organisms** | **Signatures (Nucleotides)** | | | | | | | | | |
|  |  | **M1** | **M2** | **M3** | **M4** | **M5** | **M6** | **M7** | **M8** | **M9** | **M10** |
| ***C. botulinum*** | **128** | **128 (+32)^a^** | **128** | **128 (+1)** | **128** | **128** | **128 (+127)** | **128**  **(+9)** | **128 (+102)** | **128** | **128**  **(+2)** |
| *C. perfringens* | 92 | 87 | 89 | 87 | 0 | 90 | 0 | 88 | 83 | 91 | 0 |
| *C. butyricum* | 32 | 29 | 32 | 32 | 32 | 31 | 31 | 32 | 31 | 32 | 30 |
| *C. acetobutylicum* | 24 | 24 | 24 | 24 | 22 | 24 | 7 | 5 | 5 | 24 | 23 |
| *C. beijerinckii* | 23 | 23 | 22 | 23 | 23 | 23 | 23 | 23 | 23 | 23 | 20 |
| *C. novyi* | 17 | 17 | 17 | 17 | 17 | 17 | 0 | 17 | 17 | 17 | 0 |
| *C. kluyveri* | 14 | 14 | 14 | 14 | 14 | 14 | 0 | 0 | 0 | 14 | 0 |
| *C. pasteurianum* | 13 | 13 | 13 | 11 | 13 | 12 | 0 | 11 | 0 | 13 | 13 |
| *C. sporogenes* | 11 | 0 | 11 | 9 | 10 | 11 | 10 | 11 | 10 | 11 | 10 |
| *C. sardiniense* | 9 | 8 | 9 | 9 | 9 | 9 | 0 | 9 | 9 | 9 | 9 |
| *C. colicanis* | 9 | 8 | 9 | 0 | 9 | 9 | 0 | 9 | 9 | 9 | 9 |
| *C. tetani* | 8 | 0 | 8 | 8 | 7 | 8 | 0 | 8 | 7 | 8 | 8 |
| *C. subterminale* | 8 | 8 | 8 | 8 | 7 | 8 | 0 | 8 | 7 | 7 | 8 |
| *C. chauvoei* | 8 | 6 | 8 | 8 | 1 | 8 | 8 | 6 | 0 | 8 | 5 |
| *C. baratii* | 8 | 8 | 8 | 7 | 7 | 7 | 1 | 7 | 8 | 7 | 8 |
| ^a^The values in parentheses represent duplication of signature. | | | | | | | | | | | |

| **Table S6:** **Representation of motifs obtained for *Clostridium botulinum* (in groups of 83 and 45 16S rDNA sequences) (See Materials and Methods for this segregation) through stand alone MEME** **(Version 4.3.0)** **software and the frequency of their occurrence across other *Clostridium* spp. using BioEdit** (<http://www.mbio.ncsu.edu/BioEdit/page2.html>). | | | | | | | | | | | | | | |
| --- | --- | --- | --- | --- | --- | --- | --- | --- | --- | --- | --- | --- | --- | --- |
| ***Clostridium* sp.** | **No. of organisms** | **Signatures (Nucleotides)** | | | | | | | | | | | | |
|  |  | **M1** | **M2** | | **M3** | **M4** | **M5** | **M6** | | | **M7** | **M8** | **M9** | **M10** |
|  | **Group CBoI of *C. botulinum*** | | | | | | | | | | | | | |
| ***C. botulinum*** | **83** | **83** | **83** | | **83** | **83** | **83** | **83** | | | **83** | **83** | **83** | **83** |
| *C. perfringens* | 92 | 0 | 0 | | 84 | 87 | 0 | 0 | | | 0 | 0 | 0 | 0 |
| *C. butyricum* | 32 | 0 | 0 | | 30 | 0 | 0 | 31 | | | 0 | 0 | 0 | 0 |
| *C. acetobutylicum* | 24 | 0 | 0 | | 24 | 24 | 0 | 6 | | | 0 | 0 | 0 | 0 |
| *C. beijerinckii* | 23 | 0 | 0 | | 9 | 0 | 0 | 22 | | | 0 | 0 | 0 | 0 |
| *C. novyi* | 17 | 0 | 0 | | 17 | 17 | 0 | 0 | | | 0 | 0 | 0 | 0 |
| *C. kluyveri* | 14 | 0 | 0 | | 0 | 14 | 0 | 0 | | | 0 | 0 | 0 | 14 |
| *C. pasteurianum* | 13 | 0 | 0 | | 13 | 0 | 13 | 0 | | | 0 | 0 | 0 | 0 |
| *C. sporogenes* | 11 | 8 | 10 | | 9 | 11 | 10 | 11 | | | 11 | 11 | 11 | 10 |
| *C. sardiniense* | 9 | 0 | 0 | | 8 | 9 | 0 | 0 | | | 0 | 0 | 0 | 0 |
| *C. colicanis* | 9 | 0 | 0 | | 8 | 9 | 0 | 0 | | | 0 | 0 | 0 | 0 |
| *C. tetani* | 8 | 0 | 0 | | 0 | 7 | 0 | 0 | | | 0 | 0 | 0 | 0 |
| *C. subterminale* | 8 | 0 | 0 | | 0 | 8 | 0 | 4 | | | 0 | 0 | 0 | 0 |
| *C. chauvoei* | 8 | 0 | 0 | | 7 | 8 | 0 | 0 | | | 0 | 0 | 0 | 0 |
| *C. baratii* | 8 | 0 | 0 | | 8 | 6 | 0 | 0 | | | 0 | 0 | 0 | 0 |
|  | **Group CBoII, III and IV of *C. botulinum*** | | | | | | | | | | | | | |
| ***C. botulinum*** | **45** | **45** | **45** | **45** | | **45** | **45** | | **45** | **45** | | **45** | **45** | **45** |
| *C. perfringens* | 92 | 87 | 87 | 89 | | 91 | 90 | | 91 | 0 | | 91 | 0 | 88 |
| *C. butyricum* | 32 | 29 | 32 | 32 | | 32 | 31 | | 31 | 31 | | 30 | 30 | 32 |
| *C. acetobutylicum* | 24 | 24 | 24 | 24 | | 24 | 24 | | 24 | 21 | | 23 | 23 | 5 |
| *C. beijerinckii* | 23 | 23 | 23 | 22 | | 22 | 23 | | 23 | 23 | | 21 | 20 | 23 |
| *C. novyi* | 17 | 17 | 17 | 17 | | 17 | 17 | | 17 | 17 | | 17 | 17 | 17 |
| *C. kluyveri* | 14 | 14 | 14 | 14 | | 14 | 14 | | 0 | 0 | | 14 | 14 | 0 |
| *C. pasteurianum* | 13 | 13 | 11 | 13 | | 0 | 12 | | 0 | 11 | | 11 | 12 | 11 |
| *C. sporogenes* | 11 | 9 | 9 | 11 | | 10 | 11 | | 0 | 0 | | 11 | 0 | 11 |
| *C. sardiniense* | 9 | 8 | 9 | 9 | | 9 | 9 | | 9 | 9 | | 8 | 9 | 9 |
| *C. colicanis* | 9 | 8 | 0 | 9 | | 9 | 9 | | 9 | 9 | | 9 | 9 | 9 |
| *C. tetani* | 8 | 0 | 8 | 8 | | 8 | 8 | | 0 | 7 | | 8 | 8 | 0 |
| *C. subterminale* | 8 | 8 | 8 | 8 | | 7 | 8 | | 4 | 8 | | 8 | 8 | 8 |
| *C. chauvoei* | 8 | 6 | 8 | 8 | | 1 | 8 | | 8 | 7 | | 8 | 0 | 6 |
| *C. baratii* | 8 | 8 | 7 | 8 | | 7 | 7 | | 8 | 8 | | 8 | 5 | 7 |

| **Table S7:** **Representation of motifs obtained for *Clostridium perfringens* (92 16S rDNA sequences) through stand alone MEME (Version 4.3.0)** **software and the frequency of their occurrence across other *Clostridium* spp. using BioEdit**  (<http://www.mbio.ncsu.edu/BioEdit/page2.html>). | | | | | | | | | | | |
| --- | --- | --- | --- | --- | --- | --- | --- | --- | --- | --- | --- |
| ***Clostridium* sp.** | **No. of organisms** | **Signatures (Nucleotides)** | | | | | | | | | |
|  |  | **M1** | **M2** | **M3** | **M4** | **M5** | **M6** | **M7** | **M8** | **M9** | **M10** |
| ***C. perfringens*** | **92** | **92(+1)^a^** | **92** | **92(+1)** | **92** | **92** | **92** | **92** | **92** | **92** | **92(+1)** |
| *C. botulinum* | 128 | 0 | 0 | 0 | 0 | 0 | 0 | 32 | 125 | 2 | 127 |
| *C. butyricum* | 32 | 0 | 0 | 0 | 0 | 0 | 0 | 0 | 30 | 32 | 31 |
| *C. acetobutylicum* | 24 | 0 | 0 | 0 | 0 | 0 | 0 | 3 | 24 | 24 | 23 |
| *C. beijerinckii* | 23 | 0 | 0 | 0 | 0 | 0 | 0 | 22 | 23 | 21 | 23 |
| *C. novyi* | 17 | 0 | 0 | 0 | 0 | 0 | 0 | 0 | 4 | 0 | 17 |
| *C. kluyveri* | 14 | 0 | 0 | 0 | 0 | 0 | 0 | 0 | 0 | 0 | 14 |
| *C. pasteurianum* | 13 | 0 | 0 | 0 | 0 | 0 | 0 | 0 | 13 | 13 | 11 |
| *C. sporogenes* | 11 | 0 | 0 | 0 | 0 | 0 | 0 | 0 | 10 | 0 | 9 |
| *C. sardiniense* | 9 | 0 | 0 | 0 | 0 | 0 | 9 | 0 | 8 | 9 | 8 |
| *C. colicanis* | 9 | 0 | 0 | 0 | 0 | 0 | 9 | 0 | 8 | 9 | 0 |
| *C. tetani* | 8 | 0 | 0 | 0 | 0 | 0 | 0 | 0 | 0 | 0 | 8 |
| *C. subterminale* | 8 | 0 | 0 | 0 | 0 | 0 | 0 | 0 | 0 | 8 | 8 |
| *C. chauvoei* | 8 | 0 | 0 | 0 | 0 | 0 | 8 | 0 | 7 | 7 | 8 |
| *C. baratii* | 8 | 0 | 0 | 0 | 0 | 0 | 7 | 0 | 8 | 5 | 7 |
| ^a^The values in parentheses represent duplication of signature. | | | | | | | | | | | |

| **Table** **S8:** **Representation of motifs obtained for *Clostridium butyricum* (32 16S rDNA sequences) through MEME** (<http://meme.sdsc.edu/meme4_4_0/cgi-bin/meme.cgi>) **software and the frequency of their occurrence across other *Clostridium* spp. using BioEdit**  (<http://www.mbio.ncsu.edu/BioEdit/page2.html>). | | | | | | | | | | | |
| --- | --- | --- | --- | --- | --- | --- | --- | --- | --- | --- | --- |
| ***Clostridium* sp.** | **No. of organisms** | **Signatures (Nucleotides)** | | | | | | | | | |
|  |  | **M1** | **M2** | **M3** | **M4** | **M5** | **M6** | **M7** | **M8** | **M9** | **M10** |
| ***C. butyricum*** | **32** | **32** | **32** | **32** | **32** | **32** | **32** | **32** | **32** | **32** | **32** |
| *C. botulinum* | 128 | 0 | 0 | 0 | 32 | 126 | 0 | 32 | 42 | 0 | 0 |
| *C. acetobutylicum* | 24 | 23 | 21 | 0 | 5 | 24 | 5 | 6 | 21 | 0 | 1 |
| *C. beijerinckii* | 23 | 23 | 17 | 0 | 21 | 23 | 21 | 23 | 22 | 0 | 23 |
| *C. novyi* | 17 | 0 | 0 | 0 | 0 | 17 | 0 | 0 | 17 | 0 | 0 |
| *C. kluyveri* | 14 | 0 | 0 | 0 | 0 | 14 | 0 | 0 | 0 | 0 | 0 |
| *C. pasteurianum* | 13 | 0 | 0 | 0 | 0 | 11 | 0 | 0 | 13 | 0 | 0 |
| *C. sporogenes* | 11 | 0 | 0 | 0 | 0 | 8 | 0 | 0 | 0 | 0 | 0 |
| *C. sardiniense* | 9 | 0 | 0 | 0 | 0 | 9 | 0 | 0 | 9 | 0 | 0 |
| *C. colicanis* | 9 | 0 | 0 | 0 | 9 | 0 | 0 | 0 | 9 | 0 | 0 |
| *C. tetani* | 8 | 0 | 0 | 0 | 0 | 8 | 0 | 0 | 7 | 0 | 0 |
| *C. subterminale* | 8 | 0 | 0 | 0 | 0 | 8 | 0 | 0 | 7 | 0 | 0 |
| *C. chauvoei* | 8 | 0 | 0 | 0 | 7 | 8 | 0 | 0 | 7 | 0 | 0 |
| *C. baratii* | 8 | 0 | 0 | 0 | 1 | 7 | 0 | 0 | 7 | 0 | 1 |

| **Table S9: Representation of motifs obtained for *Clostridium acetobutylicum* (24 16S rDNA sequences) through MEME** (<http://meme.sdsc.edu/meme4_4_0/cgi-bin/meme.cgi>) **software and the frequency of their occurrence across other *Clostridium* spp. using BioEdit** (<http://www.mbio.ncsu.edu/BioEdit/page2.html>). | | | | | | | | | | | |
| --- | --- | --- | --- | --- | --- | --- | --- | --- | --- | --- | --- |
| ***Clostridium* sp.** | **No. of organisms** | **Signatures (Nucleotides)** | | | | | | | | | |
|  |  | **M1** | **M2** | **M3** | **M4** | **M5** | **M6** | **M7** | **M8** | **M9** | **M10** |
| ***C. acetobutylicum*** | **24** | **24(+1)^a^** | **24(+1)** | **24(+5)** | **24** | **24** | **24(+28)** | **24** | **24** | **24** | **24** |
| *C. botulinum* | 128 | 125 | 126 | 96 | 116 | 91 | 128(+69) | 34 | 40 | 28 | 126 |
| *C. perfringens* | 92 | 84 | 87 | 89 | 0 | 91 | 91 | 91 | 0 | 63 | 91 |
| *C. butyricum* | 32 | 30 | 32 | 32 | 30 | 32 | 32 | 31 | 31 | 31 | 32 |
| *C. beijerinckii* | 23 | 23 | 23 | 22 | 23 | 22 | 22 | 23 | 23 | 23 | 23 |
| *C. novyi* | 17 | 17 | 17 | 17 | 0 | 17 | 17 | 0 | 17 | 17 | 17 |
| *C. kluyveri* | 14 | 0 | 14 | 14 | 0 | 14 | 14 | 0 | 0 | 0 | 14 |
| *C. pasteurianum* | 13 | 13 | 11 | 13 | 12 | 13 | 0 | 0 | 0 | 12 | 13 |
| *C. sporogenes* | 11 | 9 | 9 | 11 | 9 | 11 | 10 | 0 | 0 | 0 | 11 |
| *C. sardiniense* | 9 | 8 | 9 | 9 | 0 | 9 | 9 | 9 | 0 | 9 | 9 |
| *C. colicanis* | 9 | 8 | 0 | 9 | 0 | 9 | 9 | 9 | 0 | 9 | 9 |
| *C. tetani* | 8 | 0 | 8 | 7 | 0 | 8 | 8 | 8 | 0 | 8 | 8 |
| *C. subterminale* | 8 | 0 | 8 | 8 | 0 | 8 | 7 | 4 | 4 | 8 | 8 |
| *C. chauvoei* | 8 | 7 | 8 | 8 | 0 | 8 | 1 | 1 | 8 | 8 | 8 |
| *C. baratii* | 8 | 8 | 7 | 8 | 0 | 5 | 7 | 8 | 1 | 7 | 7 |
| ^a^The values in parentheses represent duplication of signature. | | | | | | | | | | | |

| **Table S10:** **Representation of motifs obtained for *Clostridium beijerinckii* (23 16S rDNA sequences) through MEME** (<http://meme.sdsc.edu/meme4_4_0/cgi-bin/meme.cgi>) **software and the frequency of their occurrence across other *Clostridium* spp. using BioEdit** (<http://www.mbio.ncsu.edu/BioEdit/page2.html>). | | | | | | | | | | | |
| --- | --- | --- | --- | --- | --- | --- | --- | --- | --- | --- | --- |
| ***Clostridium* sp.** | **No. of organisms** | **Signatures (Nucleotides)** | | | | | | | | | |
|  |  | **M1** | **M2** | **M3** | **M4** | **M5** | **M6** | **M7** | **M8** | **M9** | **M10** |
| ***C. beijerinckii*** | **23** | **23** | **23** | **23** | **23** | **23** | **23** | **23** | **23** | **23** | **23** |
| *C. botulinum* | 128 | 32 | 32 | 0 | 125 | 41 | 0 | 42 | 120 | 112 | 0 |
| *C. perfringens* | 92 | 0 | 0 | 0 | 84 | 0 | 0 | 0 | 87 | 0 | 0 |
| *C. butyricum* | 32 | 31 | 30 | 32 | 30 | 31 | 31 | 31 | 32 | 30 | 0 |
| *C. acetobutylicum* | 24 | 4 | 6 | 23 | 24 | 23 | 6 | 21 | 21 | 24 | 0 |
| *C. novyi* | 17 | 0 | 0 | 0 | 17 | 17 | 0 | 17 | 17 | 0 | 0 |
| *C. kluyveri* | 14 | 0 | 0 | 0 | 0 | 0 | 0 | 0 | 14 | 0 | 0 |
| *C. pasteurianum* | 13 | 0 | 0 | 0 | 13 | 11 | 0 | 13 | 13 | 12 | 0 |
| *C. sporogenes* | 11 | 0 | 0 | 0 | 9 | 0 | 0 | 0 | 9 | 9 | 0 |
| *C. sardiniense* | 9 | 0 | 0 | 0 | 8 | 0 | 0 | 9 | 8 | 0 | 0 |
| *C. colicanis* | 9 | 9 | 0 | 0 | 8 | 0 | 0 | 9 | 0 | 0 | 0 |
| *C. tetani* | 8 | 0 | 0 | 0 | 0 | 0 | 0 | 7 | 8 | 0 | 0 |
| *C. subterminale* | 8 | 0 | 0 | 0 | 0 | 4 | 0 | 7 | 8 | 0 | 0 |
| *C. chauvoei* | 8 | 8 | 0 | 0 | 7 | 0 | 0 | 7 | 8 | 0 | 0 |
| *C. baratii* | 8 | 1 | 0 | 0 | 8 | 1 | 1 | 7 | 7 | 0 | 0 |

| **Table S11:** **Representation** **of motifs obtained for *Clostridium novyi* (17 16S rDNA sequences) through MEME** (<http://meme.sdsc.edu/meme4_4_0/cgi-bin/meme.cgi>) **software and the frequency of their occurrence across other *Clostridium* spp. using BioEdit** (<http://www.mbio.ncsu.edu/BioEdit/page2.html>). | | | | | | | | | | | |
| --- | --- | --- | --- | --- | --- | --- | --- | --- | --- | --- | --- |
| ***Clostridium* sp.** | **No. of organisms** | **Signatures (Nucleotides)** | | | | | | | | | |
|  |  | **M1** | **M2** | **M3** | **M4** | **M5** | **M6** | **M7** | **M8** | **M9** | **M10** |
| ***C. novyi*** | **17** | **17** | **17** | **17** | **17** | **17** | **17** | **17** | **17** | **17** | **17** |
| *C. botulinum* | 128 | 9 | 8 | 9 | 40 | 41 | 8 | 9 | 8 | 126 | 3 |
| *C. perfringens* | 92 | 0 | 86 | 0 | 0 | 0 | 0 | 1 | 0 | 87 | 0 |
| *C. butyricum* | 32 | 0 | 0 | 0 | 31 | 0 | 0 | 0 | 0 | 32 | 0 |
| *C. acetobutylicum* | 24 | 0 | 17 | 0 | 1 | 0 | 0 | 0 | 0 | 24 | 0 |
| *C. beijerinckii* | 23 | 0 | 0 | 0 | 2 | 0 | 0 | 0 | 0 | 23 | 0 |
| *C. kluyveri* | 14 | 0 | 14 | 0 | 0 | 0 | 0 | 0 | 14 | 14 | 0 |
| *C. pasteurianum* | 13 | 0 | 0 | 0 | 0 | 0 | 0 | 0 | 12 | 11 | 0 |
| *C. sporogenes* | 11 | 0 | 0 | 0 | 0 | 0 | 0 | 0 | 0 | 9 | 0 |
| *C. sardiniense* | 9 | 0 | 0 | 0 | 0 | 0 | 0 | 0 | 0 | 9 | 0 |
| *C. colicanis* | 9 | 0 | 0 | 0 | 0 | 0 | 0 | 0 | 0 | 0 | 0 |
| *C. tetani* | 8 | 0 | 0 | 0 | 0 | 0 | 0 | 0 | 0 | 8 | 0 |
| *C. subterminale* | 8 | 0 | 0 | 0 | 0 | 0 | 0 | 0 | 0 | 8 | 0 |
| *C. chauvoei* | 8 | 0 | 0 | 0 | 0 | 0 | 0 | 0 | 0 | 9 | 0 |
| *C. baratii* | 8 | 0 | 0 | 0 | 0 | 1 | 0 | 0 | 0 | 7 | 0 |

| **Table S12:** **Representation of motifs obtained for *Clostridium kluyveri* (14 16S rDNA sequences) through MEME** (<http://meme.sdsc.edu/meme4_4_0/cgi-bin/meme.cgi>) **software and the frequency of their occurrence across other *Clostridium* spp. using BioEdit** (<http://www.mbio.ncsu.edu/BioEdit/page2.html>). | | | | | | | | | | | |
| --- | --- | --- | --- | --- | --- | --- | --- | --- | --- | --- | --- |
| ***Clostridium* sp.** | **No. of organisms** | **Signatures (Nucleotides)** | | | | | | | | | |
|  |  | **M1** | **M2** | **M3** | **M4** | **M5** | **M6** | **M7** | **M8** | **M9** | **M10** |
| ***C. kluyveri*** | **14** | **14** | **14** | **14** | **14** | **14** | **14** | **14** | **14** | **14** | **14** |
| *C. botulinum* | 128 | 2 | 0 | 96 | 0 | 0 | 8 | 126 | 90 | 0 | 0 |
| *C. perfringens* | 92 | 0 | 0 | 73 | 0 | 0 | 0 | 86 | 0 | 0 | 0 |
| *C. butyricum* | 32 | 0 | 0 | 0 | 0 | 0 | 0 | 32 | 0 | 0 | 0 |
| *C. acetobutylicum* | 24 | 0 | 0 | 0 | 0 | 0 | 0 | 24 | 0 | 0 | 0 |
| *C. beijerinckii* | 23 | 0 | 0 | 0 | 0 | 0 | 0 | 9 | 0 | 0 | 0 |
| *C. novyi* | 17 | 0 | 0 | 17 | 0 | 0 | 17 | 17 | 17 | 0 | 0 |
| *C. pasteurianum* | 13 | 0 | 0 | 0 | 0 | 0 | 12 | 11 | 0 | 0 | 0 |
| *C. sporogenes* | 11 | 0 | 0 | 11 | 0 | 0 | 0 | 9 | 11 | 0 | 0 |
| *C. sardiniense* | 9 | 0 | 0 | 9 | 0 | 0 | 0 | 9 | 0 | 0 | 0 |
| *C. colicanis* | 9 | 0 | 0 | 9 | 0 | 0 | 0 | 0 | 0 | 0 | 0 |
| *C. tetani* | 8 | 0 | 0 | 7 | 0 | 0 | 0 | 8 | 8 | 0 | 0 |
| *C. subterminale* | 8 | 8 | 0 | 8 | 0 | 0 | 0 | 8 | 0 | 0 | 0 |
| *C. chauvoei* | 8 | 0 | 0 | 8 | 0 | 0 | 0 | 8 | 0 | 0 | 0 |
| *C. baratii* | 8 | 0 | 0 | 6 | 0 | 0 | 0 | 7 | 0 | 0 | 0 |

| **Table S13:** **Representation of motifs obtained for *Clostridium pasteurianum* (13 16S rDNA sequences) through MEME** (<http://meme.sdsc.edu/meme4_4_0/cgi-bin/meme.cgi>) **software and the frequency of their occurrence across other *Clostridium* spp. using BioEdit** (<http://www.mbio.ncsu.edu/BioEdit/page2.html>). | | | | | | | | | | | |
| --- | --- | --- | --- | --- | --- | --- | --- | --- | --- | --- | --- |
| ***Clostridium* sp.** | **No. of organisms** | **Signatures (Nucleotides)** | | | | | | | | | |
|  |  | **M1** | **M2** | **M3** | **M4** | **M5** | **M6** | **M7** | **M8** | **M9** | **M10** |
| ***C. pasteurianum*** | **13** | **13** | **13** | **13** | **13(+1)^a^** | **13** | **13** | **13** | **13** | **13** | **13** |
| *C. botulinum* | 128 | 2 | 0 | 0 | 125 | 0 | 0 | 0 | 0 | 42 | 126 |
| *C. perfringens* | 92 | 0 | 0 | 0 | 84 | 0 | 0 | 0 | 0 | 0 | 87 |
| *C. butyricum* | 32 | 0 | 0 | 0 | 32 | 0 | 0 | 0 | 0 | 31 | 32 |
| *C. acetobutylicum* | 24 | 0 | 0 | 0 | 24 | 0 | 0 | 0 | 0 | 21 | 21 |
| *C. beijerinckii* | 23 | 0 | 0 | 0 | 23 | 0 | 0 | 0 | 0 | 23 | 23 |
| *C. novyi* | 17 | 0 | 0 | 0 | 17 | 0 | 0 | 0 | 0 | 17 | 17 |
| *C. kluyveri* | 14 | 0 | 0 | 0 | 0 | 0 | 0 | 0 | 0 | 0 | 14 |
| *C. sporogenes* | 11 | 0 | 0 | 0 | 9 | 0 | 0 | 0 | 0 | 0 | 9 |
| *C. sardiniense* | 9 | 0 | 0 | 0 | 8 | 0 | 0 | 0 | 0 | 9 | 8 |
| *C. colicanis* | 9 | 0 | 0 | 0 | 9 | 0 | 0 | 0 | 0 | 9 | 0 |
| *C. tetani* | 8 | 0 | 0 | 0 | 0 | 0 | 0 | 0 | 0 | 7 | 0 |
| *C. subterminale* | 8 | 8 | 0 | 0 | 0 | 0 | 0 | 0 | 0 | 7 | 8 |
| *C. chauvoei* | 8 | 0 | 0 | 0 | 7 | 0 | 0 | 0 | 0 | 8 | 8 |
| *C. baratii* | 8 | 0 | 0 | 0 | 8 | 0 | 0 | 0 | 0 | 7 | 7 |
| ^a^The values in parentheses represent duplication of signature. | | | | | | | | | | | |

| **Table S14:** **Representation of motifs obtained for *Clostridium sporogenes* (11 16S rDNA sequences) through MEME** (<http://meme.sdsc.edu/meme4_4_0/cgi-bin/meme.cgi>) **software and the frequency of their occurrence across other *Clostridium* spp. using BioEdit**  (<http://www.mbio.ncsu.edu/BioEdit/page2.html>). | | | | | | | | | | | |
| --- | --- | --- | --- | --- | --- | --- | --- | --- | --- | --- | --- |
| ***Clostridium* sp.** | **No. of organisms** | **Signatures (Nucleotides)** | | | | | | | | | |
|  |  | **M1** | **M2** | **M3** | **M4** | **M5** | **M6** | **M7** | **M8** | **M9** | **M10** |
| ***C. sporogenes*** | **11** | **11** | **11** | **11** | **11(+1)^a^** | **11** | **11** | **11** | **11** | **11** | **11** |
| *C. botulinum* | 128 | 0 | 96 | 28 | 116 | 90 | 82 | 85 | 85 | 96 | 85 |
| *C. perfringens* | 92 | 0 | 87 | 0 | 0 | 0 | 0 | 0 | 0 | 0 | 0 |
| *C. butyricum* | 32 | 0 | 0 | 0 | 0 | 0 | 0 | 0 | 0 | 0 | 0 |
| *C. acetobutylicum* | 24 | 0 | 0 | 0 | 0 | 0 | 0 | 0 | 0 | 18 | 0 |
| *C. beijerinckii* | 23 | 0 | 0 | 0 | 22 | 22 | 0 | 0 | 0 | 0 | 0 |
| *C. novyi* | 17 | 0 | 17 | 0 | 0 | 17 | 0 | 0 | 0 | 17 | 0 |
| *C. kluyveri* | 14 | 0 | 14 | 0 | 0 | 14 | 0 | 0 | 0 | 0 | 14 |
| *C. pasteurianum* | 13 | 0 | 0 | 0 | 0 | 0 | 0 | 0 | 0 | 11 | 0 |
| *C. sardiniense* | 9 | 0 | 9 | 0 | 0 | 0 | 0 | 0 | 0 | 0 | 0 |
| *C. colicanis* | 9 | 0 | 9 | 0 | 0 | 0 | 0 | 0 | 0 | 0 | 0 |
| *C. tetani* | 8 | 0 | 7 | 0 | 0 | 8 | 0 | 0 | 0 | 8 | 0 |
| *C. subterminale* | 8 | 0 | 8 | 0 | 4 | 0 | 0 | 0 | 0 | 8 | 0 |
| *C. chauvoei* | 8 | 0 | 8 | 0 | 0 | 0 | 0 | 0 | 0 | 0 | 0 |
| *C. baratii* | 8 | 0 | 6 | 0 | 0 | 0 | 0 | 0 | 0 | 0 | 0 |
| ^a^The values in parentheses represent duplication of signature. | | | | | | | | | | | |

| **Table S15:** **Representation of motifs obtained for *Clostridium colicanis* (9 16S rDNA sequences) through MEME** (<http://meme.sdsc.edu/meme4_4_0/cgi-bin/meme.cgi>) **software and the frequency of their occurrence across other *Clostridium* spp. using BioEdit** (<http://www.mbio.ncsu.edu/BioEdit/page2.html>). | | | | | | | | | | | |
| --- | --- | --- | --- | --- | --- | --- | --- | --- | --- | --- | --- |
| ***Clostridium* sp.** | **No. of organisms** | **Signatures (Nucleotides)** | | | | | | | | | |
|  |  | **M1** | **M2** | **M3** | **M4** | **M5** | **M6** | **M7** | **M8** | **M9** | **M10** |
| ***C. colicanis*** | **9** | **9** | **9** | **9** | **9** | **9** | **9** | **9** | **9** | **9** | **9** |
| *C. botulinum* | 128 | 0 | 32 | 0 | 0 | 0 | 0 | 96 | 0 | 0 | 42 |
| *C. perfringens* | 92 | 0 | 0 | 0 | 0 | 0 | 0 | 87 | 91 | 0 | 0 |
| *C. butyricum* | 32 | 0 | 32 | 0 | 0 | 0 | 17 | 0 | 0 | 0 | 32 |
| *C. acetobutylicum* | 24 | 0 | 5 | 0 | 0 | 0 | 1 | 0 | 0 | 0 | 21 |
| *C. beijerinckii* | 23 | 0 | 21 | 0 | 0 | 0 | 0 | 0 | 0 | 0 | 23 |
| *C. novyi* | 17 | 0 | 0 | 0 | 0 | 0 | 0 | 0 | 0 | 0 | 17 |
| *C. kluyveri* | 14 | 0 | 0 | 0 | 0 | 0 | 0 | 14 | 0 | 0 | 0 |
| *C. pasteurianum* | 13 | 0 | 0 | 0 | 0 | 0 | 0 | 0 | 0 | 0 | 13 |
| *C. sporogenes* | 11 | 0 | 0 | 0 | 0 | 0 | 0 | 11 | 0 | 0 | 0 |
| *C. sardiniense* | 9 | 0 | 0 | 0 | 9 | 0 | 0 | 9 | 9 | 0 | 9 |
| *C. tetani* | 8 | 0 | 0 | 0 | 0 | 0 | 0 | 7 | 0 | 0 | 7 |
| *C. subterminale* | 8 | 0 | 0 | 0 | 0 | 0 | 0 | 8 | 0 | 0 | 7 |
| *C. chauvoei* | 8 | 0 | 7 | 0 | 0 | 0 | 0 | 8 | 0 | 0 | 7 |
| *C. baratii* | 8 | 0 | 1 | 0 | 4 | 0 | 0 | 6 | 7 | 0 | 7 |

| **Table S16:** **Representation of motifs obtained for *Clostridium sardiniense* (9 16S rDNA sequences) through MEME** (<http://meme.sdsc.edu/meme4_4_0/cgi-bin/meme.cgi>) **software and the frequency of their occurrence across other *Clostridium* spp. using BioEdit** (<http://www.mbio.ncsu.edu/BioEdit/page2.html>). | | | | | | | | | | | |
| --- | --- | --- | --- | --- | --- | --- | --- | --- | --- | --- | --- |
| ***Clostridium* sp.** | **No. of organisms** | **Signatures (Nucleotides)** | | | | | | | | | |
|  |  | **M1** | **M2** | **M3** | **M4** | **M5** | **M6** | **M7** | **M8** | **M9** | **M10** |
| ***C. sardiniense*** | **9** | **9** | **9** | **9** | **9** | **9** | **9** | **9** | **9** | **9** | **9** |
| *C. botulinum* | 128 | 0 | 0 | 0 | 0 | 96 | 0 | 126 | 34 | 0 | 0 |
| *C. perfringens* | 92 | 0 | 0 | 0 | 0 | 87 | 91 | 87 | 0 | 0 | 0 |
| *C. butyricum* | 32 | 0 | 0 | 0 | 0 | 0 | 0 | 32 | 0 | 0 | 0 |
| *C. acetobutylicum* | 24 | 0 | 0 | 1 | 0 | 0 | 0 | 24 | 0 | 0 | 0 |
| *C. beijerinckii* | 23 | 0 | 0 | 0 | 0 | 0 | 0 | 23 | 0 | 0 | 0 |
| *C. novyi* | 17 | 0 | 0 | 0 | 0 | 17 | 0 | 17 | 0 | 0 | 0 |
| *C. kluyveri* | 14 | 0 | 0 | 0 | 0 | 14 | 0 | 14 | 0 | 0 | 0 |
| *C. pasteurianum* | 13 | 0 | 0 | 0 | 0 | 0 | 0 | 11 | 0 | 0 | 0 |
| *C. sporogenes* | 11 | 0 | 0 | 0 | 0 | 11 | 0 | 9 | 1 | 0 | 0 |
| *C. colicanis* | 9 | 0 | 0 | 0 | 0 | 9 | 9 | 0 | 0 | 0 | 0 |
| *C. tetani* | 8 | 0 | 0 | 0 | 0 | 7 | 0 | 8 | 7 | 0 | 0 |
| *C. subterminale* | 8 | 0 | 0 | 0 | 0 | 8 | 0 | 8 | 0 | 0 | 0 |
| *C. chauvoei* | 8 | 0 | 0 | 0 | 0 | 8 | 0 | 8 | 0 | 0 | 0 |
| *C. baratii* | 8 | 5 | 6 | 7 | 6 | 6 | 7 | 7 | 7 | 3 | 7 |

| **Table S17:** **Representation of motifs obtained for *Clostridium baratii* (8 16S rDNA sequences) through MEME** (<http://meme.sdsc.edu/meme4_4_0/cgi-bin/meme.cgi>) **software and the frequency of their occurrence across other** ***Clostridium* spp. using BioEdit** (<http://www.mbio.ncsu.edu/BioEdit/page2.html>). | | | | | | | | | | | |
| --- | --- | --- | --- | --- | --- | --- | --- | --- | --- | --- | --- |
| ***Clostridium* sp.** | **No. of organisms** | **Signatures (Nucleotides)** | | | | | | | | | |
|  |  | **M1** | **M2** | **M3** | **M4** | **M5** | **M6** | **M7** | **M8** | **M9** | **M10** |
| ***C. baratii*** | **8** | **8(+6)^a^** | **8** | **8** | **8** | **8** | **8** | **8** | **8** | **8** | **8** |
| *C. botulinum* | 128 | 125 | 32 | 0 | 43 | 128 | 0 | 0 | 42 | 32 | 34 |
| *C. perfringens* | 92 | 84 | 0 | 0 | 0 | 89 | 0 | 0 | 0 | 54 | 91 |
| *C. butyricum* | 32 | 30 | 31 | 0 | 31 | 32 | 0 | 30 | 31 | 0 | 31 |
| *C. acetobutylicum* | 24 | 24 | 5 | 0 | 23 | 24 | 0 | 6 | 23 | 18 | 24 |
| *C. beijerinckii* | 23 | 23 | 23 | 0 | 23 | 22 | 0 | 23 | 23 | 22 | 23 |
| *C. novyi* | 17 | 17 | 0 | 0 | 17 | 17 | 0 | 0 | 17 | 0 | 0 |
| *C. kluyveri* | 14 | 0 | 0 | 0 | 0 | 14 | 0 | 0 | 0 | 14 | 0 |
| *C. pasteurianum* | 13 | 13 | 0 | 0 | 12 | 13 | 0 | 0 | 11 | 0 | 0 |
| *C. sporogenes* | 11 | 8 | 0 | 0 | 0 | 10 | 0 | 0 | 0 | 0 | 0 |
| *C. sardiniense* | 9 | 8 | 0 | 8 | 8 | 9 | 9 | 9 | 9 | 9 | 9 |
| *C. colicanis* | 9 | 8 | 9 | 0 | 1 | 9 | 0 | 0 | 9 | 9 | 9 |
| *C. tetani* | 8 | 0 | 0 | 0 | 7 | 8 | 0 | 0 | 7 | 0 | 0 |
| *C. subterminale* | 8 | 0 | 0 | 0 | 8 | 0 | 0 | 8 | 8 | 0 | 4 |
| *C. chauvoei* | 8 | 7 | 8 | 0 | 8 | 8 | 0 | 0 | 7 | 8 | 8 |
| ^a^The values in parentheses represent duplication of signature. | | | | | | | | | | | |

| **Table S18:** **Representation of motifs obtained for *Clostridium chauvoei* (8 16S rDNA sequences) through MEME** (<http://meme.sdsc.edu/meme4_4_0/cgi-bin/meme.cgi>) **software and the frequency of their occurrence across other *Clostridium* spp. using BioEdit**  (<http://www.mbio.ncsu.edu/BioEdit/page2.html>). | | | | | | | | | | | |
| --- | --- | --- | --- | --- | --- | --- | --- | --- | --- | --- | --- |
| ***Clostridium* sp.** | **No. of organisms** | **Signatures (Nucleotides)** | | | | | | | | | |
|  |  | **M1** | **M2** | **M3** | **M4** | **M5** | **M6** | **M7** | **M8** | **M9** | **M10** |
| ***C. chauvoei*** | **8** | **8** | **8** | **8** | **8** | **8** | **8** | **8** | **8(+9)^a^** | **8** | **8** |
| *C. botulinum* | 128 | 0 | 32 | 0 | 0 | 0 | 96 | 126 | 0 | 0 | 116 |
| *C. perfringens* | 92 | 0 | 0 | 0 | 0 | 0 | 87 | 87 | 0 | 0 | 0 |
| *C. butyricum* | 32 | 0 | 31 | 0 | 0 | 0 | 0 | 32 | 0 | 0 | 31 |
| *C. acetobutylicum* | 24 | 0 | 5 | 0 | 0 | 0 | 0 | 24 | 0 | 0 | 7 |
| *C. beijerinckii* | 23 | 0 | 22 | 0 | 0 | 0 | 0 | 23 | 0 | 0 | 23 |
| *C. novyi* | 17 | 0 | 0 | 0 | 0 | 0 | 17 | 17 | 0 | 0 | 0 |
| *C. kluyveri* | 14 | 0 | 0 | 0 | 0 | 0 | 14 | 14 | 0 | 0 | 0 |
| *C. pasteurianum* | 13 | 0 | 0 | 0 | 0 | 0 | 0 | 11 | 0 | 0 | 0 |
| *C. sporogenes* | 11 | 0 | 0 | 0 | 0 | 0 | 11 | 9 | 0 | 0 | 10 |
| *C. sardiniense* | 9 | 0 | 0 | 0 | 0 | 0 | 9 | 9 | 0 | 0 | 0 |
| *C. colicanis* | 9 | 0 | 9 | 0 | 0 | 0 | 9 | 0 | 0 | 0 | 0 |
| *C. tetani* | 8 | 0 | 0 | 0 | 0 | 0 | 7 | 8 | 0 | 0 | 0 |
| *C. subterminale* | 8 | 0 | 0 | 0 | 0 | 0 | 8 | 8 | 0 | 0 | 0 |
| *C. baratii* | 8 | 0 | 1 | 0 | 0 | 0 | 6 | 7 | 0 | 0 | 1 |
| ^a^The values in parentheses represent duplication of signature. | | | | | | | | | | | |

| **Table S19:** **Representation of motifs obtained for *Clostridium subterminale* (8 16S rDNA sequences) through MEME** (<http://meme.sdsc.edu/meme4_4_0/cgi-bin/meme.cgi>) **software and the frequency of their occurrence across other *Clostridium* spp. using BioEdit** (<http://www.mbio.ncsu.edu/BioEdit/page2.html>). | | | | | | | | | | | |
| --- | --- | --- | --- | --- | --- | --- | --- | --- | --- | --- | --- |
| ***Clostridium* sp.** | **No. of organisms** | **Signatures (Nucleotides)** | | | | | | | | | |
|  |  | **M1** | **M2** | **M3** | **M4** | **M5** | **M6** | **M7** | **M8** | **M9** | **M10** |
| ***C. subterminale*** | **8** | **8** | **12** | **8** | **8** | **8** | **8** | **8** | **8** | **8** | **16** |
| *C. botulinum* | 128 | 2 | 2 | 2 | 2 | 96 | 2 | 126 | 42 | 91 | 9 |
| *C. perfringens* | 92 | 0 | 0 | 0 | 0 | 87 | 0 | 87 | 0 | 91 | 0 |
| *C. butyricum* | 32 | 0 | 0 | 0 | 0 | 0 | 0 | 32 | 31 | 32 | 0 |
| *C. acetobutylicum* | 24 | 0 | 0 | 18 | 18 | 0 | 0 | 24 | 21 | 24 | 0 |
| *C. beijerinckii* | 23 | 0 | 0 | 0 | 0 | 0 | 0 | 23 | 23 | 22 | 0 |
| *C. novyi* | 17 | 0 | 0 | 0 | 0 | 17 | 0 | 17 | 17 | 17 | 17 |
| *C. kluyveri* | 14 | 0 | 14 | 0 | 0 | 14 | 0 | 14 | 0 | 14 | 14 |
| *C. pasteurianum* | 13 | 13 | 0 | 0 | 0 | 0 | 0 | 11 | 11 | 13 | 0 |
| *C. sporogenes* | 11 | 0 | 0 | 0 | 0 | 11 | 0 | 9 | 0 | 11 | 0 |
| *C. sardiniense* | 9 | 0 | 0 | 0 | 0 | 9 | 0 | 9 | 9 | 9 | 0 |
| *C. colicanis* | 9 | 0 | 0 | 0 | 0 | 9 | 0 | 0 | 9 | 9 | 0 |
| *C. tetani* | 8 | 0 | 0 | 0 | 0 | 7 | 0 | 8 | 7 | 8 | 0 |
| *C. chauvoei* | 8 | 0 | 0 | 0 | 0 | 8 | 0 | 8 | 7 | 8 | 8 |
| *C. baratii* | 8 | 0 | 0 | 0 | 0 | 0 | 0 | 7 | 8 | 5 | 1 |

| **Table S20:** **Representation of motifs obtained for *Clostridium tetani* (8 16S rDNA sequences) through MEME** (<http://meme.sdsc.edu/meme4_4_0/cgi-bin/meme.cgi>) **software and the frequency of their occurrence across other *Clostridium* spp. using BioEdit** (<http://www.mbio.ncsu.edu/BioEdit/page2.html>). | | | | | | | | | | | |
| --- | --- | --- | --- | --- | --- | --- | --- | --- | --- | --- | --- |
| ***Clostridium* sp.** | **No. of organisms** | **Signatures (Nucleotides)** | | | | | | | | | |
|  |  | **M1** | **M2** | **M3** | **M4** | **M5** | **M6** | **M7** | **M8** | **M9** | **M10** |
| ***C. tetani*** | **8** | **8** | **8** | **8** | **8** | **8** | **8** | **8** | **8** | **8** | **8** |
| *C. botulinum* | 128 | 0 | 0 | 0 | 0 | 0 | 0 | 0 | 0 | 0 | 126 |
| *C. perfringens* | 92 | 0 | 0 | 0 | 0 | 0 | 0 | 0 | 0 | 0 | 92 |
| *C. butyricum* | 32 | 0 | 0 | 0 | 0 | 0 | 0 | 0 | 0 | 0 | 32 |
| *C. acetobutylicum* | 24 | 0 | 0 | 0 | 0 | 0 | 0 | 0 | 0 | 0 | 24 |
| *C. beijerinckii* | 23 | 0 | 0 | 0 | 0 | 0 | 0 | 0 | 0 | 0 | 23 |
| *C. novyi* | 17 | 0 | 0 | 0 | 0 | 0 | 0 | 0 | 0 | 0 | 17 |
| *C. kluyveri* | 14 | 0 | 0 | 0 | 0 | 0 | 0 | 0 | 0 | 0 | 1 |
| *C. pasteurianum* | 13 | 0 | 0 | 0 | 0 | 0 | 0 | 0 | 0 | 0 | 11 |
| *C. sporogenes* | 11 | 0 | 0 | 0 | 0 | 0 | 0 | 0 | 0 | 0 | 9 |
| *C. sardiniense* | 9 | 0 | 0 | 0 | 0 | 0 | 0 | 0 | 0 | 0 | 9 |
| *C. colicanis* | 9 | 0 | 0 | 0 | 0 | 0 | 0 | 0 | 0 | 0 | 9 |
| *C. subterminale* | 8 | 0 | 0 | 0 | 0 | 0 | 0 | 0 | 0 | 0 | 8 |
| *C. chauvoei* | 8 | 0 | 0 | 0 | 0 | 0 | 0 | 0 | 0 | 0 | 8 |
| *C. baratii* | 8 | 0 | 0 | 0 | 0 | 0 | 0 | 0 | 0 | 0 | 7 |

| **Table S21:** **Representation of motifs obtained for *Clostridium* species (unsegregated) through MEME** (<http://meme.sdsc.edu/meme4_4_0/cgi-bin/meme.cgi>) **software and their occurrence across other *Clostridium* sp. using** **BioEdit** (<http://www.mbio.ncsu.edu/BioEdit/page2.html>). | | | | | | | | | | |
| --- | --- | --- | --- | --- | --- | --- | --- | --- | --- | --- |
| ***Clostridium* sp.^a^** | **Nucleotide signatures obtained through MEME** | | | | | | | | | |
|  | **M1** | **M2** | **M3** | **M4** | **M5** | **M6** | **M7** | **M8** | **M9** | **M10** |
| ***Clostridium* sp. strains close to *C. subterminale*** | | | | | | | | | | |
| Frame work sequences | + | + | + | + | + | U^b^ | + | + | + | + |
| S000129667 | + | + | + | + | + | + | + | + | + | + |
| S000388864 | + | + | + | + | + | + | + | + | + | + |
| S000388865 | + | + | + | + | + | + | + | + | + | + |
| S000388866 | + | + | + | − | + | + | + | + | + | + |
| S000416645 | + | + | + | + | + | − | + | + | + | + |
| S000435104 | + | + | + | + | + | + | + | + | + | + |
| S000485866 | + | + | + | + | + | − | + | + | + | + |
| S000503799 | + | + | + | − | + | − | + | + | + | + |
| S000690901 | + | − | + | + | + | − | + | + | + | + |
| S000735015 | + | + | + | + | + | + | + | − | + | + |
| S000735016 | + | + | + | + | + | + | + | + | + | − |
| S000749591 | + | + | + | + | + | + | + | + | + | − |
| S000749594 | + | + | + | + | + | + | + | + | + | + |
| S000749595 | + | + | + | + | + | + | + | + | + | + |
| S001152397 | + | + | + | + | + | + | + | + | + | + |
| S001170637 | + | + | + | + | + | + | + | + | + | + |
| S001170638 | + | − | + | + | + | − | + | + | + | + |
| S001199642 | + | + | + | + | + | + | + | + | + | + |
| S001199651 | + | + | + | + | + | + | + | + | + | + |
| S000001962 | + | + | + | + | + | + | + | + | + | + |
| ***Clostridium* sp. strains close to *C. beijerinckii*** | | | | | | | | | | |
| Frame work sequences | + | + | + | + | + | + | + | + | + | U |
| S000334985 | − | + | − | − | − | − | + | + | − | − |
| S000334987 | + | + | + | + | + | + | + | + | + | − |
| S000334988 | − | + | + | + | + | + | + | + | + | − |
| S000334990 | + | + | + | + | + | − | + | + | + | − |
| S000400860 | − | + | + | + | + | − | + | + | + | − |
| S000400864 | − | − | + | + | + | + | + | + | + | − |
| S000478687 | + | − | + | + | + | + | + | + | − | − |
| S000626915 | + | − | + | + | + | + | + | + | + | − |
| S000722515 | + | − | + | + | + | + | + | + | + | − |
| S000728324 | + | − | − | + | + | + | + | + | + | − |
| S000980699 | + | − | + | + | + | + | + | + | + | − |
| S001152399 | − | + | − | + | + | − | + | + | + | − |
| S001152400 | + | + | + | + | + | − | + | + | + | − |
| ***Clostridium* sp. strains close to *C. kluyveri*** | | | | | | | | | | |
| Frame work sequences | + | U | + | U | U | + | + | + | U | U |
| S000005987 | + | − | + | − | − | − | − | + | − | − |
| S000127639 | + | − | + | − | − | − | − | + | − | − |
| S000511412 | + | − | + | − | − | − | − | + | − | − |
| ***Clostridium* sp. strains close to *C. tetani*** | | | | | | | | | | |
| Frame work sequences | U | U | U | U | U | U | U | U | U | + |
| S000263019 | + | + | + | + | + | + | + | + | + | + |
| S000386952 | + | − | − | + | + | − | − | − | − | − |
| S000485868 | + | − | − | − | − | − | − | − | + | + |
| S000530910 | + | + | + | + | + | + | + | + | + | + |
| S001152394 | − | − | − | − | − | − | − | − | − | + |
| S001199640 | + | − | − | − | + | + | − | + | + | + |
| S001244389 | + | − | − | + | + | − | − | + | + | + |
| ***Clostridium* sp. strains close to *C. chauvoei*** | | | | | | | | | | |
| Frame work sequences | U | + | U | U | U | + | + | U | U | + |
| S000012343 | − | − | − | − | − | − | − | − | + | − |
| S000389535 | − | + | + | + | − | + | + | + | − | − |
| S000485865 | − | − | − | − | − | + | + | + | − | + |
| S001155550 | − | + | + | − | − | + | + | − | − | + |
| ***Clostridium* sp. strains close to *C. perfringens*** | | | | | | | | | | |
| Frame work sequences | U | U | U | U | U | + | + | + | + | + |
| S000392900 | + | + | + | + | + | − | + | + | + | + |
| ***Clostridium* sp. strains close to *C. baratii* and *C. sardiniense*** | | | | | | | | | | |
| Frame work sequences | + | + | U | + | + | U | + | + | + | + |
| S000626914 | + | − | + | + | + | + | + | + | + | + |
| S001244388 | + | − | + | + | + | + | + | + | + | + |
| S001187265 | + | − | + | + | + | + | + | + | + | + |
| S001187266 | + | − | + | + | + | + | + | + | + | + |
| S001187267 | + | − | + | + | + | + | + | + | + | + |
| ***Clostridium* sp. strains close to *C. pasteurianum*** | | | | | | | | | | |
| Frame work sequences | + | U | U | + | U | + | U | U | + | + |
| S000334962 | − | − | + | − | + | − | − | + | + | + |
| S000511411 | + | − | + | + | + | + | + | + | + | + |
| S001153886 | + | − | + | + | + | + | + | + | + | + |
| S001170673 | + | + | + | − | + | + | + | − | + | + |
| ***Clostridium* sp. strains close to *C. novyi*** | | | | | | | | | | |
| Frame work sequences | U | + | U | + | + | U | + | + | + | U |
| S000016396 | − | + | − | + | + | − | − | + | + | + |
| S001415996 | − | + | − | − | + | − | − | + | + | − |
| ***Clostridium* sp. strains close to *C. sporogenes*** | | | | | | | | | | |
| Frame work sequences | U | + | + | + | + | + | + | + | + | + |
| S000357670 | − | − | + | + | + | + | + | − | + | + |
| S000375671 | − | + | + | + | + | + | + | + | + | + |
| S000408659 | − | + | − | − | + | − | − | − | − | − |
| ***Clostridium* sp. strains close to *C. acetobutylicum*^c^** | | | | | | | | | | |
| Frame work sequences | + | + | + | + | + | + | + | + | + | + |
| S000400861 | + | + | + | + | + | + | − | + | + | + |
| S000400862 | + | + | − | + | + | − | − | + | + | + |
| S000400863 | + | + | + | + | + | + | + | + | + | + |
| S000435107 | + | + | + | + | + | + | + | − | + | + |
| S000478679 | + | + | + | − | − | + | + | − | + | + |
| S000478680 | + | + | + | − | − | + | + | − | + | + |
| S000478681 | + | + | + | − | + | + | + | − | + | + |
| S000478682 | + | + | + | − | + | + | + | − | + | + |
| S000478683 | + | + | + | − | − | + | + | − | + | + |
| S000478684 | + | + | + | + | + | + | − | − | + | + |
| S000478685 | + | + | − | − | + | + | + | − | + | + |
| S000478686 | + | + | + | − | + | + | − | − | + | + |
| S000943243 | + | + | + | − | + | + | + | − | + | + |
| S000966301 | + | + | + | − | + | + | + | − | + | + |
| S000980425 | + | + | + | − | + | + | + | − | + | + |
| S000995877 | + | + | + | + | + | + | + | − | + | + |
| S001020065 | + | + | + | + | − | + | − | − | + | + |
| S001152396 | + | + | + | + | + | + | + | + | + | + |
| S001242068 | + | + | + | − | + | + | + | − | + | + |
| S001745227 | + | + | − | + | + | + | + | + | + | + |
| S001745233 | + | − | + | + | + | + | + | + | + | + |
| S001794681 | − | + | − | − | + | + | + | − | + | + |
| S001794682 | + | + | + | − | + | + | + | − | + | + |
| S001794683 | + | + | + | − | + | + | + | − | + | + |
| S001794684 | + | + | + | − | + | + | + | − | + | + |
| S001794685 | + | + | + | − | + | + | + | − | + | + |
| S001794686 | + | − | + | − | + | + | + | − | + | + |
| S001794687 | + | + | + | − | + | + | + | − | + | + |
| S001794688 | + | + | + | − | + | + | + | − | + | + |
| ***Clostridium* sp. strains close to *C. botulinum*^c^** | | | | | | | | | | |
| Frame work sequences | + | + | + | + | + | + | + | + | + | + |
| S001152392 | + | + | − | − | + | − | + | + | + | + |
| S000728313 | − | − | − | − | − | − | − | − | − | − |
| S000357670 | + | + | − | − | − | − | + | + | + |  |
| S000375671 | + | + | − | − | − | − | + | + | + | + |
| S001199641 |  | + | − | − | + | − | + | + | + | + |
| ^a^Ribosomal Database Accession Number of those *Clostridium* sp. which were segregated on the basis of phylogenetic frame work sequences. | | | | | | | | | | |
| ^b^Signatures found to be unique to the respective *Clostridium* species (Table 8) | | | | | | | | | | |
| ^c^No unique signature were detected for *C. acetobutylicum* and *C. botulinum* (Table 8) | | | | | | | | | | |
