# Supplementary material for: Analysis of the unexplored features of rrs (16S rDNA) of the Genus Clostridium
Source: BMC Genomics. 2011 Jan 11;12:18. doi: 10.1186/1471-2164-12-18 (PMC3024285; doi:10.1186/1471-2164-12-18)
Supplement: Additional file 10 — Table S22 Nucleotide signatures. File contains nucleotide signatures of rrs sequences common to Clostridium sp. used for developing framework sequences. [file 1471-2164-12-18-S10.DOC]

| **Table S22: Nucleotide signatures of 16S rDNA sequences common to *Clostridium* sp. used for developing framework sequences.** | | | |
| --- | --- | --- | --- |
| Nucleotide signature | | | |
| Sequence | Initial position^a^ | Reference | |
|  |  | Organism | Motif |
| 5’AACTACGTGCCAGCAGCCGCGGTAATACGT3’ | 394-474(m) | *Clostridium botulinum* | M9 |
| 5’AACTACGTGCCAGCAGCCGCGGTAATACGT3’ | 440-474(m) | *C. acetobutylicum* | M10 |
| 5’AAACAGGATTAGATACCCTGGTAGTCCACG3’ | 559-745(m) | *C. acetobutylicum* | M5 |
| 5’AATTCCTAGTGTAGCGGTGAAATGCGTAGA3’ | 560-640(m) | *C. botulinum* | M2 |
| 5’AAATGCGTAGAGATTAGGAAGAACACCAGT3’ | 591-666 | *C. sporogenes* | M2 |
| 5’AATTCCTAGTGTAGCGGTGAAATGCGTAGA3’ | 618-649 | *C. baratii* | M5 |
| 5’AAAGCGTGGGGAGCAAACAGGATTAGATAC3’ | 667-733(m) | *C. perfringens* | M9 |
| 5’AGTACGGTCGCAAGATTAAAACTCAAAGGA3’ | 747-864(m) | *C. perfringens* | M8 |
| 5’TCGCAAGATTAAAACTCAAAGGAATTGACG3’ | 782-861(m)^a^ | *C. botulinum* | M1 |
| 5’ATGTGGTTTAATTCGAAGCAACGCGAAGAA3’ | 828-921, 876(m) | *C. butyricum* | M5 |
| 5’GTTTAATTCGAAGCAACGCGAAGAACCTTA3’ | 840-919(m) | *C. botulinum* | M3 |
| 5’ACGGTCGCAAGATTAAAACTCAAAGGAATT3’ | 856(m)-864 | *C. beijerinckii* | M4 |
| 5’TAATTCGAAGCAACGCGAAGAACCTTACCT3’ | 894-932(m) | *C. perfringens* | M10 |
| 5’AGCATGTGGTTTAATTCGAAGCAACGCGAA3’ | 910(m)-918 | *C. beijerinckii* | M8 |
| 5’ATGGTTGTCGTCAGCTCGTGTCGTGAGATG3’ | 940-1020(m) | *C. botulinum* | M5 |
| 5’AGGAAGGTGGGGATGACGTCAAATCATCAT3’ | 952-1142(m) | *C. acetobutylicum* | M6 |
| 5’ATGACGTCAAATCATCATGCCCCTTATGTC 3’ | 1070-1170,1150(m) | *C. botulinum* | M4 |
| 5’AAATCATCATGCCCCTTATGTCTAGGGCTA3’ | 1156(m)-1164 | *C. beijerinckii* | M7 |
| ^a^The signatures in different organisms were located at different positions and (m).denotes the starting position with maximum frequency | | | |
