# Supplementary material for: Analysis of the unexplored features of rrs (16S rDNA) of the Genus Clostridium
Source: BMC Genomics. 2011 Jan 11;12:18. doi: 10.1186/1471-2164-12-18 (PMC3024285; doi:10.1186/1471-2164-12-18)
Supplement: Additional file 11 — Tables S23-S26 Representative motifs for Clostridium botulinum and C. perfringens. File contains details of rrs sequences of Clostridium botulinum and C. perfringens used for drawing Regular Expression Diagram of signatures presented in Additional file 8: Figures S20-S23. [file 1471-2164-12-18-S11.DOC]

| **Table S23:**  **Details of 128 16S rDNA sequences of *Clostridium botulinum* used for drawing Regular Expression Diagram of signatures presented in Figure S20.** | | |
| --- | --- | --- |
| **Representative Sequence** | **Other members** | **Total** |
| S000260169 |  | 1 |
| S000016030 |  | 1 |
| S001094755 | S001094712 | 2 |
| S000805089 |  | 1 |
| S000414703 |  | 1 |
| S000858493 | S000858488 S000858491 S000858484 S000858486 S000858480 S000858482 S000858476 S000858478 | 9 |
| S000414702 |  | 1 |
| S000414707 | S000414699 S000414701 | 3 |
| S000414706 |  | 1 |
| S000015318 | S000001308 S000006955 S001094770 | 4 |
| S000414700 |  | 1 |
| S000260209 |  | 1 |
| S000260538 |  | 1 |
| S001094707 | S001094714 S001094705 S001094718 S001094703 S001094724 S001094720 S001094722 S001094716 S001094772 S001094766 S001094768 S001094762 S001094764 S001094758 S001094760 S001094753 S001094751 | 19 |
| S000428963 |  | 1 |
| S000260030 |  | 1 |
| S000406355 | S000805083 S000805086 S000805087 S000805088 | 5 |
| S001416097 | S001416092 S001416095 S001416088 S001416090 S001416084 S001416086 S001416080 S001416082 S001350522 S001350523 S001350519 S001350521 S001350517 S001350518 S001350515 S001350516 S001350513 S001014501 S001014496 S001014499 S001014492 S001014494 S001014488 S001014490 S001014484 S001014486 S001014407 S001014409 S001014402 S001014404 S001014398 S001014400 S001014394 S001014396 S000891623 S001014392 S000891618 S000891614 S000891616 S000891610 S000891612 S000891606 S000891608 S000891603 S000891605 S000891598 S000891600 S000891594 S000891596 S000891590 S000891592 S000891587 S000891589 S000891580 S000891582 S000891584 S000891576 S000891578 S000891574 S000260418 S000260307 | 62 |
| S001199656 |  | 1 |
| S000805091 | S000805092 S000805090 | 3 |
| S000805084 |  | 1 |
| S001611496 |  | 1 |
| S000805085 |  | 1 |
| S000260029 |  | 1 |
| S000414704 | S000414705 | 2 |
| S001611495 |  | 1 |
| S001611494 |  | 1 |
| Total | | 128 |

| **Table S24:**  **Details of 83 16S rDNA sequences of *Clostridium botulinum* (CBoI) used for drawing Regular Expression diagram of signatures presented in Figure S21.** | | |
| --- | --- | --- |
| **Representative Sequence** | **Other members** | **Total** |
| S000805090 | S000805091, S000805092 | 3 |
| S000260538 |  | 1 |
| S000414700 |  | 1 |
| S000414703 |  | 1 |
| S000858476 | S000858478, S000858480, S000858484, S000858486, S000858491, S000858493, S000414699, S000414701, S000414707 | 10 |
| S000428963 |  | 1 |
| S000260209 |  | 1 |
| S000891574 | S000891576, S000891578, S000891582, S000891587, S000891589, S000891590, S000891592, S000891594, S000891603, S000891605, S000891606, S000891608, S000891610, S000891612, S000891614, S000891616, S000891618, S000891621, S000891623, S001014392, S001014394, S001014396, S001014398, S001014400, S001014402, S001014404, S001014407, S001014409, S001014484, S001014486, S001014488, S001014490, S001014492, S001014494, S001014496, S001014499, S001014501, S001350513, S001350515, S001350516, S001350517, S001350518, S001350519, S001350521, S001350522, S001350523, S001416080, S001416082, S001416084, S001416086, S001416088, S001416090, S001416092, S001416095, S001416097, S000016030, S000260307, S000260418, S000891598 | 60 |
| S000858482 |  | 1 |
| S000891580 | S000891596 | 2 |
| S000891584 | S000891600 | 2 |
| Total | | 83 |

| **Table S25:**  **Details of 45 16S rDNA sequences of *Clostridium botulinum* (CBoII-IV) used for drawing Regular Expression diagram of signatures presented in Figure S22.** | | |
| --- | --- | --- |
| **Representative Sequence** | **Other members** | **Total** |
| S000406355 |  | 1 |
| S000805084 |  | 1 |
| S000805085 |  | 1 |
| S000805083 | S000805087, S000805088 | 3 |
| S000805089 |  | 1 |
| S000805086 |  | 1 |
| S001094770 |  | 1 |
| S001611494 | S001611495 | 2 |
| S001611496 |  | 1 |
| S001199656 |  | 1 |
| S000858488 | S000414702 | 2 |
| S000414704 | S000414705 | 2 |
| S000414706 |  | 1 |
| S001094722 | S001094720, S001094712, S001094705, S001094724, S001094710, S001094718, S001094714, S001094703, S001094716, S001094707, S001094758, S001094755, S001094760, S001094753, S001094762, S001094751, S001094764, S001094768, S001094772, S001094766, S000015318, S000001308, S000006955 | 24 |
| S000260030 |  | 1 |
| S000260169 |  | 1 |
| S000260029 |  | 1 |
| Total |  | 45 |

| **Table S26: Details of 92 16S rDNA sequences of *Clostridium perfringens* used for drawing Regular Expression diagram of signatures presented in Figure S23*.*** | | |
| --- | --- | --- |
| **Representative Sequence** | **Other members** | **Total** |
| S000721521 | S000721523, S000721516, S000721518, S000721512, S000721514, S000721508, S000721510, S000004722. S000383292, S000383294, S000383296, S000383298, S000383300, S000383302, S000383304, S000383308, S000383310, S000436641, S000528323, S000528325, S000528327, S000528329, S000528331, S000528333, S000528335, S000528338, S000528340, S000528342 | 29 |
| S001199663 | S001199645, S001199648, S001199650, S001199653, S001155553, S001155552, S000605751, S000605752, S000605748, S000605749, S000605750, S000605747, S000605742, S000605743, S000605744, S000605745, S000605746 | 18 |
| S001588467 | S001588465, S001588466 | 3 |
| S001588457 | S001588455 | 2 |
| S001588449 | S001588470 | 2 |
| S001588460 |  | 1 |
| S000941936 |  | 1 |
| S000941937 |  | 1 |
| S000941935 |  | 1 |
| S000383306 |  | 1 |
| S000701619 |  | 1 |
| S001169268 |  | 1 |
| S001169266 |  | 1 |
| S000941941 |  | 1 |
| S001588471 |  | 1 |
| S001588453 |  | 1 |
| S001588474 |  | 1 |
| S001588451 |  | 1 |
| S001588452 |  | 1 |
| S000941940 |  | 1 |
| S000941938 |  | 1 |
| S000701621 |  | 1 |
| S001588468 |  | 1 |
| S000941939 |  | 1 |
| S000436468 |  | 1 |
| S001588456 |  | 1 |
| S000436640 |  | 1 |
| S001588461 |  | 1 |
| S001549690 |  | 1 |
| S001588469 |  | 1 |
| S001045994 |  | 1 |
| S000941933 |  | 1 |
| S000616319 |  | 1 |
| S001588475 |  | 1 |
| S001588473 |  | 1 |
| S000941934 |  | 1 |
| S000004508 |  | 1 |
| S001588458 |  | 1 |
| S001588459 |  | 1 |
| S001588454 |  | 1 |
| S001588450 |  | 1 |
| S001588448 |  | 1 |
| S000941932 |  | 1 |
| Total |  | 92 |
